# Effects of perioperative benzodiazepine administration on postoperative patient-reported outcomes: a systematic review and meta-analysis of randomised controlled trials

**DOI:** 10.1016/j.bja.2025.09.013

**Published:** 2025-09-30

**Authors:** Fang Zhou Ge, Karen Zhao, Emily Au, Behnam Sadeghirad, Renée Fournier, Emilie Belley-Côté, Jack Young, Eugene Wang, Chris Beaver, Shelley Kloppenburg, David Mazer, Eric Jacobsohn, Michael Verret, Jessica Spence

**Affiliations:** 1 Department of Anesthesiology and Pain Medicine, University of Toronto, Toronto, ON, Canada; 2 Department of Family Medicine, University of Toronto, Toronto, ON, Canada; 3 Department of Anesthesiology, Pharmacology, and Therapeutics, University of British Columbia, Vancouver, BC, Canada; 4 Department of Anesthesia, McMaster University, Hamilton, ON, Canada; 5 Department of Health Research Methods, Evaluation, and Impact, McMaster University, Hamilton, ON, Canada; 6 Faculty of Science, McMaster University, Hamilton, ON, Canada; 7 Department of Medicine (Cardiology and Critical Care), McMaster University, Hamilton, ON, Canada; 8 Perioperative Research Division, Population Health Research Institute, Hamilton, ON, Canada; 9 Health Sciences Library, McMaster University, Hamilton, ON, Canada; 10 Patient/family partner; 11 Department of Anesthesia and Li Ka Shing Knowledge Institute, St. Michael’s Hospital, Toronto, ON, Canada; 12 Department of Anesthesia and Perioperative Medicine, University of Manitoba, Winnipeg, MB, Canada; 13 Département d’anesthésiologie et de soins intensifs, Faculté de médecine, Université Laval, Quebec, QC, Canada; 14 Population Health and Optimal Health Practices Research Unit (Trauma–Emergency–Critical Care Medicine), CHU de Québec–Université Laval Research Centre, Quebec, QC, Canada; 15 Department of Critical Care, McMaster University, Hamilton, ON, Canada

**Keywords:** benzodiazepines, meta-analysis, patient-reported outcomes, perioperative medicine, systematic review

## Abstract

**Background:**

Benzodiazepines are administered during the perioperative period because they are believed to improve the patient experience. We conducted a systematic review and meta-analysis evaluating the effect of perioperative benzodiazepines on postoperative patient-reported outcomes.

**Methods:**

We included randomised controlled trials of all languages comparing benzodiazepines with non-benzodiazepine comparators in adults undergoing inpatient surgery. Our outcomes were postoperative pain, quality of recovery, patient satisfaction, and anxiety. We assessed risk of bias using a modified Cochrane tool. We pooled data using a random-effects model and assessed the quality of evidence for each outcome using the Grading of Recommendations, Assessment, Development, and Evaluation approach.

**Results:**

We screened 33 220 abstracts, 1156 full texts, and 123 RCTs, of which 93 were included in the quantitative synthesis. We identified moderate certainty evidence that benzodiazepines have no effect on patient-reported postoperative pain or quality of recovery and very low certainty evidence that benzodiazepines have no effect on patient satisfaction. We identified low certainty evidence that benzodiazepines result in higher levels of postoperative anxiety (22 trials, *n*=2165; mean difference, 2.18 points on the 20–80 State-Trait Anxiety Inventory Scale; 95% confidence interval, 1.05–3.30; *I*^2^=90%).

**Conclusions:**

Benzodiazepine administration in the perioperative period probably has no effect on postoperative pain or quality of recovery and has an uncertain effect on patient satisfaction (very low certainty evidence). When compared with placebo or another agent, benzodiazepines might result in higher levels of postoperative anxiety. Definitive research is required to determine whether the balance of risks and benefits justifies using benzodiazepines during the perioperative period to improve patient-reported outcomes.

**Systematic review protocol:**

PROSPERO (CRD42023440326).

**Editor’s key points**

• Benzodiazepines are frequently administered in the perioperative period, often in an attempt to enhance the patient’s experience. However, their effects on pain, quality of recovery, satisfaction, and anxiety are uncertain.
• This systematic review and meta-analysis of 123 RCTs shows that benzodiazepines do not improve pain or quality of recovery, have uncertain effects on satisfaction, and might increase postoperative anxiety.
• Benzodiazepines should not be used with the intention to improve the patient experience. Future trials should evaluate alternatives for optimising patient-centred outcomes.

Benzodiazepines are commonly used by anaesthetists for perioperative anxiolysis, sedation, and amnesia. They are frequently administered while obtaining vascular access, performing regional nerve blocks, and for procedural sedation, or as a premedication or a component of anaesthetic induction. Given their anxiolytic and anti-nauseant properties, perioperative benzodiazepines are given to enhance postoperative patient satisfaction, which has been recognised as an indicator of the quality of perioperative care.^1^^,^^2^ In current practice, 16–89% of patients undergoing cardiac and noncardiac surgery are administered a perioperative benzodiazepine, depending on patient age and surgical population.^3^, ^4^, ^5^

Despite their frequent use, recent reports have alluded to the potential harms of perioperative benzodiazepines. Benzodiazepine administration during the perioperative period to patients who are benzodiazepine naive has been associated with postoperative benzodiazepine dependence.^6^ Because of the association between benzodiazepine exposure and delirium, the American Geriatric Society and the Society of Critical Care Medicine have recommended that perioperative benzodiazepines not be given to older and critically ill patients.^7^^,^^8^ As a result, the most recent Perioperative Brain Health Guidelines have recommended that perioperative benzodiazepine use be avoided in patients at risk of postoperative delirium.^9^

In light of potential benefits and harms, we conducted a systematic review and meta-analysis evaluating the effects of perioperative benzodiazepine administration.^10^ We have previously reported the outcomes of perioperative delirium,^11^ intraoperative awareness,^11^ and postoperative nausea and vomiting (PONV).^12^ Our focus for this systematic review is on four patient-reported outcomes: postoperative pain, anxiety, satisfaction, and quality of recovery. In contrast to clinician-measured outcomes, patient-reported outcomes are measures that provide important patient perspectives of treatment benefits and harms that may not otherwise be captured using traditional metrics of morbidity and mortality.

## Methods

We published a protocol for the overarching review (*BMJ Open,* PMID 31831540)^10^ and registered the protocol describing this work with PROSPERO (CRD42023440326). We followed the Preferred Reporting Items for Systematic Reviews and Meta-Analyses (PRISMA) reporting guidelines.^12^

### Data sources

We used a search strategy developed by a medical librarian as part of a broader review on perioperative benzodiazepine administration.^10^ Database specific subject headings and keywords were used to search for key concepts related to benzodiazepines and surgery (Supplementary Appendix 1). We searched EMBASE, MEDLINE, PsychINFO, CINAHL, Web of Science, and Cochrane CENTRAL from inception to September 13, 2024, without language restrictions. Conference proceedings indexed in Embase were included in the quantitative synthesis if sufficient detail was reported or qualitative synthesis if it was not. Clinical trial registrations are indexed in CENTRAL; when potentially relevant studies were identified, we searched for associated publications and, if none were identified, contacted investigators to ascertain study status. We did not search Google Scholar or other sources of grey literature as they frequently index publications from predatory journals not typically indexed in mainstream databases, which can lead to bias in the results of systematic reviews and meta-analyses.^13^^,^^14^ We reviewed reference lists from included trials and related reviews for additional eligible trials.

### Study selection

Two reviewers independently reviewed titles, abstracts, and full texts to determine if they met inclusion criteria. We included randomised controlled trials of adult patients undergoing cardiac or noncardiac surgery where the intervention group received perioperative benzodiazepines via any route and the control group received a non-benzodiazepine active comparator, placebo, or nothing. For this report, we included only trials reporting the following postoperative patient-reported outcomes: pain, anxiety, quality of recovery, and satisfaction. Disagreements between reviewers were resolved by consensus or involvement of a third reviewer.

### Data extraction and risk of bias assessments

A pair of reviewers independently extracted data from included studies using a standardised, pilot-tested form. We extracted data pertaining to study characteristics (authors, publication year, country of origin, study design), study population (age, sex), type of surgery (cardiac, general, obstetric, gynaecologic, orthopaedic, otolaryngologic, urologic, mixed noncardiac), intervention (benzodiazepine, dose, route and timing of administration), comparator (agent, dose, route, and timing of administration), and outcome data for pain, anxiety, quality of recovery, and patient satisfaction. The same pair of reviewers used a modified version of the Cochrane tool to assess risk of bias.^15^^,^^16^ To avoid using the ‘unclear’ risk of bias designation, the modified tool uses four response options: ‘low risk’, ‘probably low risk’, ‘probably high risk’, and ‘high risk.’^15^ We evaluated the domains of random sequence generation, allocation concealment, blinding of study participants, blinding of personnel, blinding of data collectors, blinding of outcome assessors, blinding of data analysts, incomplete outcome data, and selective outcome reporting. Concerns not otherwise captured, including those related to inappropriate sponsor influence, were categorised as ‘Other risk of bias’. We deemed trials to have an overall high risk of bias if patients were not blinded to study intervention (patient-reported outcomes), they contained at least one domain assessed to have a high risk of bias, or they contained two or more domains assessed to be probably high risk of bias.^15^ Reviewers resolved disagreements in data extraction and risk of bias assessment through discussion and, if needed, adjudication by a third reviewer.

### Data analysis

We evaluated the effects of perioperative benzodiazepines on each outcome in the short-term (<24 h postoperative) and longer-term (≥24 h postoperative). To pool studies which measured an outcome/construct using different instruments, we used linear transformation to convert measures of pain intensity to a 0- (no pain) to 10-cm (worst imaginable pain) format visual analogue scale (VAS), measures of quality of recovery to a 0–150 score Quality of Recovery 15 (QoR-15) scale,^17^ measures of patient satisfaction to a 0- (very low satisfaction) to 100-mm (very high satisfaction) VAS,^18^ and measures of anxiety to a 20–80 score State-Trait Anxiety Inventory Scale (STAI-S).^19^ We considered the minimally important clinical differences for each measure to be a pooled mean difference (MD) of 1 cm for visual analogue pain scales,^20^ a pooled MD of 6 for the QoR-15,^21^ a pooled MD of 10 mm for visual analogue satisfaction scales,^20^ and a pooled MD of 10 for the STAI-S.^22^

For postoperative pain, quality of recovery, and patient satisfaction, we used end of follow-up mean score for analysis. For anxiety, we used change scores from baseline rather than end-of-study scores to account for inter-patient variability. If the authors did not report change scores, we calculated them using the baseline and end-of-study score and a correlation coefficient of 0.5.^23^ We pooled effect estimates for all continuous outcomes reported by more than one study as the weighted mean difference (WMD) and the associated 95% confidence interval (CI) using DerSimonian–Laird random-effects model.^24^^,^^25^ *Post hoc*, we undertook a sensitivity analysis for the outcomes of patient-reported pain within and beyond 24 h after surgery including only trials where benzodiazepines were compared with placebo or nothing. Data analysis was performed in Stata (StataCorp, Release 18.5, College Station, TX, USA). Comparisons were two tailed using a threshold of *P*≤0.05.

### Assessment of heterogeneity

We assessed heterogeneity by visual inspection of forest plots, and the *I*^2^, a statistical measure of heterogeneity. Regardless of the observed statistical heterogeneity, we conducted the following subgroup analyses: (1) studies including females only compared with mixed-sex studies; (2) studies of older (≥65 yr) compared with younger (<65 yr) patients; (3) studies comparing benzodiazepines with an active comparator *vs* studies comparing benzodiazepines with placebo or nothing; (4) studies using remimazolam compared with studies using other benzodiazepines; and (5) high compared with low risk of bias studies (see Supplementary Appendix 2 for prespecified subgroup hypotheses). For all subgroups with at least two trials in each group, we tested for interaction using meta-regression with a modification to the variance of the estimated coefficients suggested by Knapp and Hartung.^26^^,^^27^ To determine the credibility of each subgroup, two independent reviewers assessed them using the Instrument for assessing the Credibility of Effect Modification Analyses (ICEMAN) criteria.^28^ We prespecified that we would consider subgroups assessed to have moderate or high credibility as possibly significant.

### Certainty of evidence assessments

We used the Grading of Recommendations, Assessment, Development, and Evaluation (GRADE) approach to assess the certainty of evidence for each outcome. In this approach, the certainty in any effect estimate is categorised as high, moderate, low, or very low considering limitations in risk of bias, consistency, directness, precision, and publication bias.^29^ For all outcomes with ≥10 studies contributing to meta-analysis, we assessed small-study effects using contour-enhanced funnel plots and performed Egger’s test.^30^ We used GRADE informative statements to communicate our findings.^31^^,^^32^

## Results

### Description of included studies

**Fig 1:**
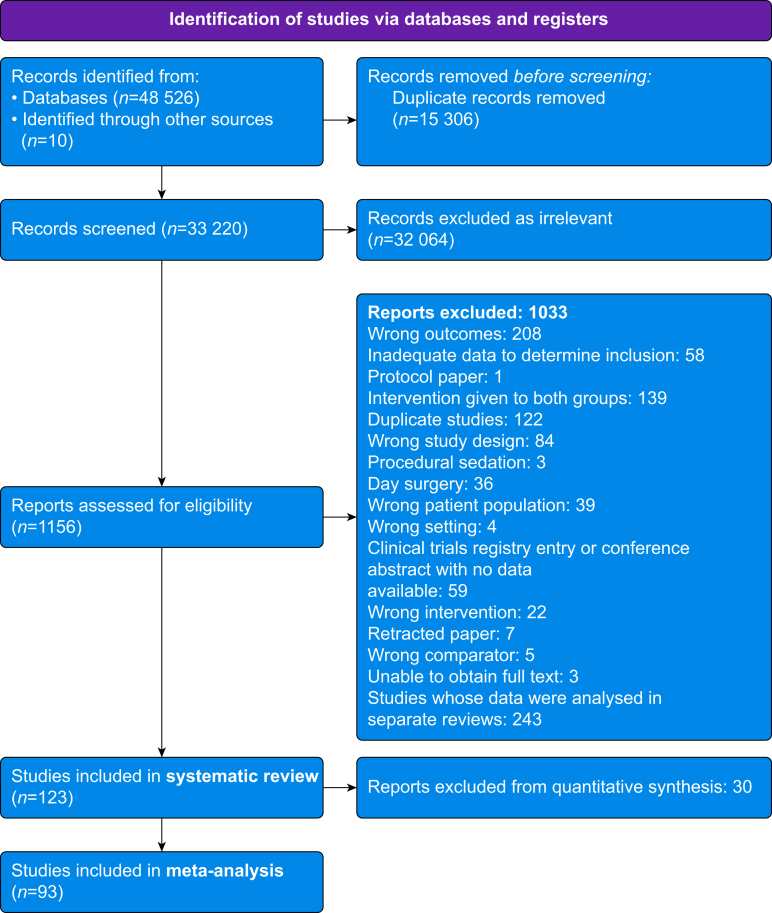
Preferred Reporting Items for Systematic reviews and Meta-Analyses (PRISMA) flow diagram.

We identified 33 220 unique records through our searches. After reviewing the titles and abstracts, we retrieved 1156 full-text articles to screen for eligibility. We included 123 RCTs (*n*=12 921 participants), of which 85 (*n*=10 458) were included in the quantitative synthesis (Fig. 1, Supplementary Appendix 3). The reasons for exclusion from quantitative synthesis are described within each outcome.

A detailed summary of included studies can be found in Supplementary Appendices 3, 4, and 5. Of the 123 included trials, the largest proportion of studies included patients undergoing abdominal surgery (*n*=32), followed by gynaecologic surgery (*n*=22), orthopaedic surgery (*n*=21), obstetrical surgery (*n*=8), otolaryngologic surgery (*n*=7), urologic surgery (*n*=6), and cardiac surgery (*n*=2). In 13 trials, type of surgery was not reported; 12 trials included a mix of noncardiac surgeries. Most trials (*n*=69) evaluated intraoperative benzodiazepine administration, 44 evaluated preoperative administration, seven evaluated postoperative administration, and three evaluated benzodiazepine administration at multiple perioperative timepoints. A variety of benzodiazepines were studied: 81 trials examined midazolam, 15 examined remimazolam, 12 examined diazepam, seven examined lorazepam, five examined alprazolam, and two examined triazolam. One trial examined both remimazolam and estazolam. Benzodiazepines were primarily administered intravenously (*n*=74), whereas 21 trials evaluated the administration of oral benzodiazepines. The remaining 27 trials evaluated intrathecal (*n*=13), intramuscular (*n*=8), sublingual (*n*=2), perineural (*n*=3), and rectal (*n*=1) administration. One trial did not report method of administration. Overall, 62 trials reported postoperative pain, 27 reported postoperative anxiety, 34 reported patient satisfaction, and 14 reported quality of recovery. Most (*n*=74) trials assessed outcomes within the first 24 h after surgery; 19 assessed outcomes after the first 24 h and 24 assessed outcomes both within and after the 24 h after surgery. Two trials assessed outcomes at the time of patient discharge without further specification, and four did not report when outcomes were assessed.

### Risk of bias

**Fig 2:**
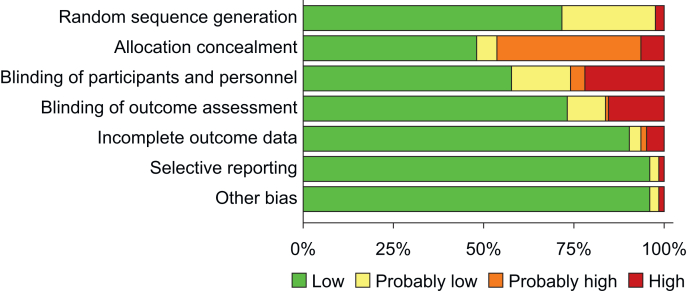
Summary of risk of bias of all included trials.

We judged the overall risk of bias to be low in 86 trials and high in 37 trials (Fig. 2, Supplementary Appendix 6). The most common domain with a high risk of bias was blinding of participants (*n*=27), followed by blinding of outcome assessment (*n*=19). Across included trials, allocation concealment was poorly described, with 57 trials assessed to have either high or probably high risk of bias because of inadequate reporting.

### Outcomes

#### Pain intensity

A total of 66 trials reported the effect of perioperative benzodiazepines on postoperative pain across all timepoints; 56 were included in the quantitative synthesis (*n*=6425). Ten studies could not be included in meta-analysis (Supplementary Appendix 5): six did not report numeric VAS values, two reported outcomes using a categorical scale, and two measured postoperative pain using a VAS but did not report any data. Among the studies not included in the quantitative synthesis, six found no difference between groups,^31^, ^32^, ^33^, ^34^, ^35^, ^36^ one favoured benzodiazepine for pain during the immediate postoperative period,^37^ and one favoured comparator over benzodiazepine.^38^

Perioperative benzodiazepines likely result in little to no difference in postoperative pain within 24 h compared with non-benzodiazepines when compared with placebo, nothing, or an active comparator (56 trials, *n*=6563; MD −0.05 cm on a 10-cm VAS; 95% CI, −0.25 to 0.15; *I*^2^=96%; moderate certainty) (Supplementary Appendices 7a and 8). Beyond 24 h after surgery, perioperative benzodiazepines probably have little to no effect on postoperative pain compared with non-benzodiazepines when compared with placebo, nothing, or an active comparator (13 trials, *n*=987; MD −0.10 cm on a 10-cm VAS; 95% CI, −0.38 to 0.19; *I*^2^=95%; moderate certainty) (Supplementary Appendices 8 and 9a). We assessed the evidence for both outcomes to be of moderate certainty as the optimal information size had been exceeded. Subgroup analysis comparing older (>65 yr) with younger patients identified a significant interaction effect, with benzodiazepines having no significant effect in younger patients (MD, −0.22 cm on a 10-cm VAS; 95% CI, −0.50 to 0.06; *I*^2^=95%) and increased pain in older patients (MD, 1.71 cm on a 10-cm VAS; 95% CI, 0.50–2.92; *I*^2^=24%) (*P* for interaction=0.004, Supplementary Appendix 10). However, as this was a between-study comparison of a small number of studies with an arbitrary cut-point for age, we assessed this to be a low-credibility subgroup (Supplementary Appendix 11). No other subgroups for both immediate postoperative and in-hospital pain were suggestive of potential effect modifiers. Our *post hoc* sensitivity analysis including only trials where benzodiazepines were compared with placebo or nothing (Supplementary Appendices 7b and 9b) was not statistically significant for either immediate postoperative or in-hospital pain. However, because each timepoint included only a small number of trials, the width of the CI for each did not exclude a possible benefit of perioperative benzodiazepines on postoperative pain.

#### Quality of recovery

Fourteen trials reported the effect of perioperative benzodiazepines on postoperative patient-reported quality of recovery across all timepoints; 11 were included in the quantitative synthesis. Three trials could not be meta-analysed (Supplementary Appendix 5). Ellingson and colleagues (1977)^39^ and Haram and colleagues (1981)^40^ studied benzodiazepines in the context of forceps and C-section delivery, respectively; both noted improved satisfaction when patients were given a benzodiazepine. Song and colleagues (2022)^41^ compared the difference between preoperative and postoperative QoR-40 scores and found that the decrement in global QoR-40 score was significantly smaller when patients undergoing laparoscopic cholecystectomy or robotic gynaecologic surgery received remimazolam compared with desflurane for maintenance of anaesthesia (MD, −7.03; 95% CI, 0.35–13.72).

**Fig 3:**
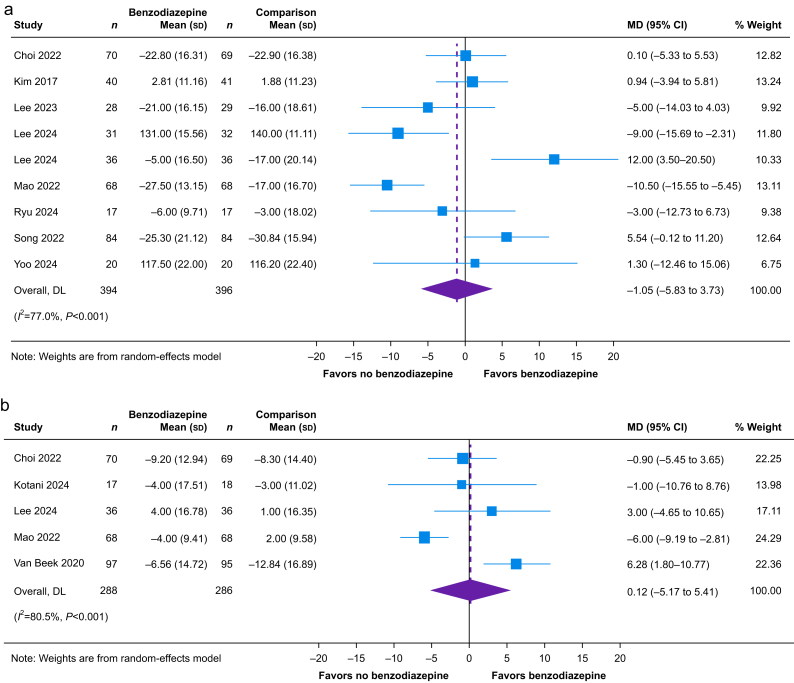
Forest plot depicting the effect of perioperative benzodiazepines on quality of recovery as quantified by change in the Quality of Recovery-15 scale (a) within and (b) beyond 24 h of surgery. 95% CI, 95% confidence interval; MD, mean difference; DL, DerSimonian-Laird.

Within 24 h of surgery, we found that benzodiazepines probably have little or no effect on quality of recovery (nine studies, *n*=790; MD −1.05 on a 0–150 QoR-15 scale; 95% CI, −5.83 to 3.73; *I*^2^=77%; moderate certainty) (Fig. 3a, Supplementary Appendix 8). Similarly, we found that, beyond 24 h after surgery, perioperative benzodiazepines likely have little or no effect on quality of recovery (five studies, *n*=574; MD, 0.12 on a 0–150 QoR-15 scale; 95% CI, −5.17 to 5.41; *I*^2^=81%; moderate certainty) (Fig. 3b, Supplementary Appendix 8). We assessed the evidence for both outcomes to be of moderate certainty as the optimal information size had been exceeded. We did not identify potential effect modifiers within prespecified subgroup analyses (Supplementary Appendices 10 and 11).

#### Patient satisfaction

**Fig 4:**
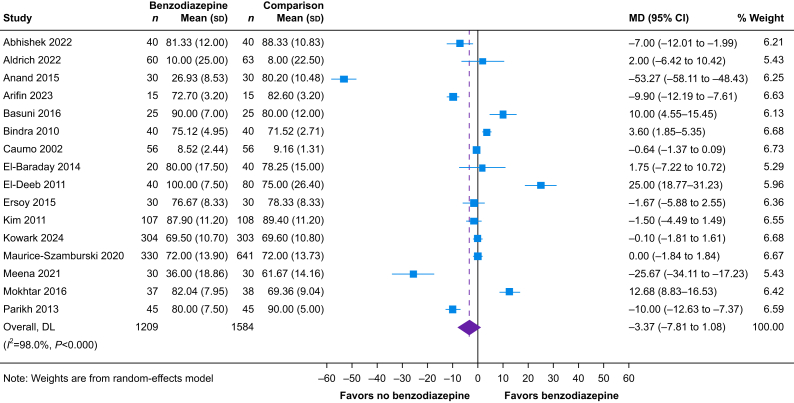
Forest plot depicting the effect of perioperative benzodiazepines on postoperative patient satisfaction as quantified by change in a 0–100-mm visual analogue scale (VAS) within 24 h of surgery. 95% CI, 95% confidence interval; MD, mean difference; DL, DerSimonian-Laird.

A total of 34 trials reported the effect of perioperative benzodiazepines on patient-reported satisfaction across all timepoints; 16 were included in the quantitative synthesis. Eighteen studies could not be pooled because their results could not be converted to a 10-cm VAS (Supplementary Appendix 5). Among these 18 studies, four reported better scores in the benzodiazepine group,^41^, ^42^, ^43^, ^44^ six reported better scores in the comparator group,^32^^,^^45^, ^46^, ^47^, ^48^, ^49^ and eight reported no significant difference between benzodiazepine and non-benzodiazepine groups.^50^, ^51^, ^52^, ^53^, ^54^, ^55^, ^56^, ^57^ Within 24 h of surgery, the evidence is very uncertain about the effect of perioperative benzodiazepines on patient satisfaction (16 studies, *n*=2793; MD, −3.37 mm on a 0–100-mm VAS; 95% CI, −7.81 to 1.08; *I*^2^=98%; very low certainty) (Fig. 4). We rated the evidence as very low certainty as the 95% CI of the pooled effect estimate did not exclude a clinically important change in postoperative patient-reported satisfaction and there were important statistical heterogeneity and variability in how the intervention was delivered. We were unable to evaluate patient satisfaction beyond 24 h after surgery owing to there being only two trials that could not be converted to a common scale. A prespecified subgroup analysis examining studies that included only female participants compared with studies including mixed sex or male patients identified a significant interaction effect, with benzodiazepines associated with increased satisfaction in studies of female patients (MD on 100-mm VAS in studies of females, 8.49 mm; 95% CI, 2.99–13.98; *I*^2^=96%; MD on 100-mm VAS in studies of mixed sex and male patients, −10.60 mm; 95% CI, −17.74 to −3.46; *I*^2^=96%) (*P* for interaction=0.023, Supplementary Appendix 10). However, owing to this being a between-study comparison of a small number of studies, we assessed this to be a low-credibility subgroup (Supplementary Appendix 11). We did not identify any other prespecified subgroups that were statistically significant.

#### Postoperative anxiety

**Fig 5:**
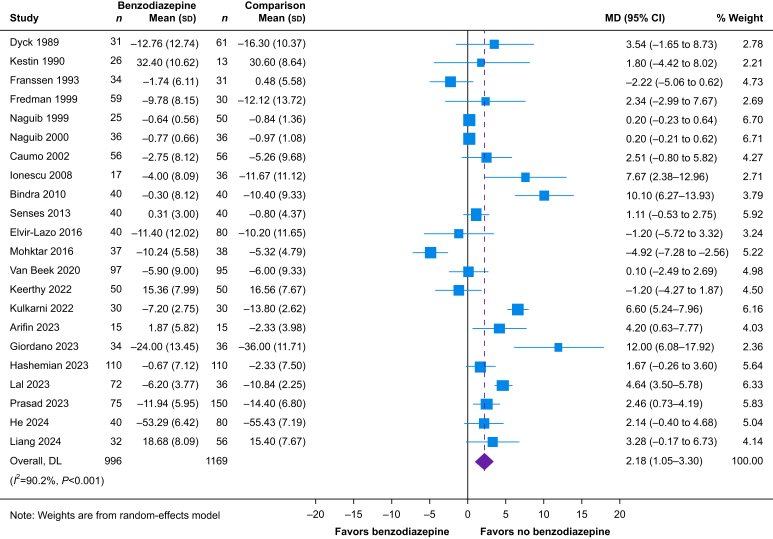
Forest plot depicting the effect of perioperative benzodiazepines on postoperative anxiety as quantified by change in State Trait Anxiety Inventory Scale within 24 h of surgery. 95% CI, 95% confidence interval; MD, mean difference; DL, DerSimonian-Laird.

We included 27 trials that reported the effect of perioperative benzodiazepines on postoperative anxiety across all timepoints; 22 were included in the quantitative synthesis. Five trials could not be pooled (Supplementary Appendix 5); one reported reduced anxiety in the benzodiazepine group^58^ and four trials reported no significant difference between the benzodiazepine and non-benzodiazepine groups.^54^^,^^59^, ^60^, ^61^ We found that, when compared with non-benzodiazepine comparators, perioperative benzodiazepines may increase anxiety during the immediate postoperative period (22 trials, *n*=2165; MD, 2.18 points on the 20–80 STAI-S; 95% CI, 1.05–3.30, *I*^2^=90.2%; low certainty) (Fig. 5, Supplementary Appendix 8). We assessed the evidence to be of low certainty as there were important statistical heterogeneity and variability in how the intervention was delivered. We were unable to evaluate anxiety beyond 24 h after surgery owing to there being only three trials that could not be converted to a common scale. We did not identify any prespecified subgroups that were statistically significant (Supplementary Appendices 10 and 11).

### Certainty of evidence assessments

Details regarding our GRADE assessment of pooled effect estimates can be found in Table 1 and Supplementary Appendix 8. We assessed for publication bias for the outcomes of short-term anxiety, short- and long-term pain, and short-term satisfaction and did not detect publication bias (Supplementary Appendix 12). The number of included trials was insufficient to assess for publication bias for the other outcomes.

**Table 1:** Grading of Recommendations Assessment, Development, and Evaluation (GRADE) summary of findings. 95% CI, 95% confidence interval; MD, mean difference. ∗Even though we identified concerns about risk of bias, the subgroup analysis did not support the presence of an interaction effect. ^†^Important statistical heterogeneity but centred around the line of no effect. ^‡^The 95% CI crossed the line of no effect but was narrower than the MCID for the outcome. ^¶^Only low risk of bias studies were included. ^§^Important statistical heterogeneity and variability in how the intervention was delivered. ^||^The 95% CI did not exclude a clinically important different in postoperative patient satisfaction.

No. of studies	No. of patients		Effect	Certainty
	Benzodiazepines	Other medications or placebos	Absolute (95% CI)	
Postoperative pain (immediately postoperative)				
56	3114	3449	MD 0.05 lower (0.24 lower to 0.15 higher)	
Postoperative pain (in-hospital)				
13	536	451	MD 0.1 lower (0.38 lower to 0.19 higher)	
Quality of recovery (immediately postoperative)				
9	394	396	MD 1.05 lower (5.83 lower to 3.73 higher)	
Quality of recovery (in hospital)				
5	288	2286	MD 0.12 higher (5.17 lower to 5.41 higher)	
Satisfaction (immediately postoperative)				
16	1209	1584	MD 3.37 lower (7.81 lower to 1.08 higher)	
Postoperative anxiety (immediately postoperative)				
22	996	1169	MD 2.18 higher (1.05 higher to 3.3 higher)	

## Discussion

In this systematic review and meta-analysis of 123 trials, we identified moderate certainty evidence that benzodiazepines have no effect on postoperative pain or quality of recovery and very low certainty evidence that benzodiazepines have no effect on patient satisfaction. Even though benzodiazepines are often given to treat preoperative anxiety, we identified low certainty evidence that, when compared with placebo or another agent, benzodiazepines result in higher levels of postoperative anxiety up to 24 h after surgery.

Although many trials have evaluated the effects of perioperative benzodiazepines on patient-reported outcomes, most have been small and performed in single centres; many are at high risk of bias. Two of the trials included in our meta-analyses stand out as being large, multicentre, and at low risk of bias. First, Kowark and colleagues (2024)^62^ evaluated the effect of 3.75 mg oral midazolam given before surgery compared with placebo on self-reported satisfaction on postoperative day 1 in 607 patients aged 65 yr and older undergoing elective, inpatient, noncardiac surgery at nine centres in Germany. Similar to the findings of our meta-analysis, they did not identify a difference in global patient satisfaction comparing patients who had received midazolam with those who had received placebo (MD, −0.2; 95% CI, −1.9 to 1.6; *P*=0.85). Second, Maurice-Szamburski and colleagues (2015)^63^ evaluated the effect of lorazepam 2.5 mg before surgery compared with no medication or placebo on self-reported satisfaction on postoperative day 1 in 1062 patients less than 70 yr of age undergoing elective noncardiac surgery under general anaesthesia at five teaching hospitals in France. Consistent with our findings, they found that premedication with lorazepam had no effect on patient satisfaction when compared with no medication or placebo (*P*=0.38), even when considering only the subgroup of patients with heightened preoperative anxiety.^63^ Further, when compared with patients given no medication or placebo, patients who had received preoperative lorazepam had longer times to extubation (*P*<0.001) and lower rates of early cognitive recovery (*P*<0.001).^63^

The harms of perioperative benzodiazepines have been previously suggested by observational studies. Poeran and colleagues^64^ conducted a retrospective cohort study of 505 152 patients undergoing hip fracture repair between 2006 and 2016 and recorded in the Premier Healthcare Database. After adjustment, they found that the use of both long-acting (odds ratio [OR], 1.82; 95% CI, 1.75–1.89) and combined short- and long-acting (OR, 1.56; 95% CI, 1.48–1.63) benzodiazepines was associated with postoperative delirium.^64^ Using the Optum Clinformatics Datamart, Gaulton and colleagues^75^ conducted a retrospective cohort study of 785 346 patients undergoing general or orthopaedic surgery. They found that benzodiazepine and non-benzodiazepine receptor agonist (‘Z-drug’) exposure in the 90 days before surgery was associated with an increase in the odds of an adverse postoperative event in the 30 days after surgery (OR, 1.13; 95% CI, 1.08–1.18). There is also evidence suggesting an association between perioperative benzodiazepine exposure in benzodiazepine-naive patients and persistent benzodiazepine use. Wright and colleagues^6^ conducted a retrospective cohort study of 2 509 599 adults undergoing both inpatient and outpatient noncardiac procedures between 2009 and 2017 that were included in the MarketScan database. They found that, among benzodiazepine-naive patients who received a perioperative benzodiazepine, the rate of persistent benzodiazepine use during the 90–180-day period after surgery was 19.5% (95%, CI 19.2–19.8%) and that, of these, 43.8% received two or more prescriptions.

There are few randomised controlled trials evaluating the effect of perioperative benzodiazepines compared with placebo or an alternative agent on adverse postoperative outcomes; the majority are small, from single centres, and at high risk of bias.^7^, ^8^, ^9^^,^^61^, ^62^, ^63^, ^64^ A recent systematic review and meta-analysis of trials found very low quality evidence that perioperative benzodiazepines did not increase the risk of postoperative delirium (eight trials, *n*=1352; relative risk [RR], 1.43; 95%, CI 0.90–2.27), but when considering only those trials comparing benzodiazepines with dexmedetomidine, benzodiazepines were more deliriogenic (five trials, *n*=429; RR, 1.83; 95%, CI 1.24–2.72). After this systematic review was published, the B-Free cluster-randomised crossover trial evaluated whether an institutional policy of benzodiazepine restriction would decrease the incidence of postoperative delirium in 19 768 patients undergoing cardiac surgery at 20 North American centres. Within the intention-to-treat analysis, restricting benzodiazepines during cardiac surgery did not reduce delirium incidence (adjusted odds ratio [aOR], 0.92; 95% CI, 0.84–1.01; *P*=0.07), but was also not associated with an increase in the incidence of patient-reported intraoperative awareness, a common reason for using benzodiazepines in the cardiac surgery population.^65^ Further, a per protocol analysis suggested a small statistically significant benefit when considering benzodiazepine restriction at the level of the individual patient, rather than the institution (aOR, 0.90; 95% CI, 0.82–0.99; *P*=0.02).^65^ Given that smaller effect sizes could not be ruled out, the authors concluded that restriction of benzodiazepines during cardiac surgery may be considered.

Within this report, we focus on the effects of perioperative benzodiazepines on the postoperative patient-reported outcomes of pain, quality of recovery, satisfaction, and anxiety, and found no evidence of benefit. Within our overarching review,^7^, ^8^, ^9^^,^^66^, ^67^, ^68^, ^69^ we have previously found that perioperative benzodiazepines probably decrease the incidence of PONV (52 trials, *n*=5086; RR, 0.77; 95% CI, 0.66–0.89; number needed to treat [NNT], 16; moderate certainty), postoperative nausea (55 studies, *n*=5916; RR, 0.72; 95% CI, 0.62–0.83; NNT, 21; moderate certainty), and postoperative vomiting (52 studies, *n*=5909; RR, 0.74; 95% CI, 0.60–0.91; NNT, 55; moderate certainty).^66^ However, despite this evidence, we found that any benefit that benzodiazepines may have for preventing PONV and related outcomes is not translated into improvements in more global indicators of the patient experience, such as quality of recovery and patient satisfaction.

In light of evidence of benefit for PONV, an outcome that is often thought to inform but not define the patient experience, and possible evidence of harm for delirium,^11^^,^^65^ an outcome prioritised by patients and other stakeholders,^67^^,^^68^ multiple guidelines and professional bodies have recommended that benzodiazepines be avoided in patients at risk of postoperative delirium.^7^, ^8^, ^9^^,^^69^, ^70^, ^71^, ^72^ Despite these recommendations, benzodiazepine administration during the perioperative period remains common.^4^^,^^5^^,^^73^^,^^74^ The reasons for the persistent use of perioperative benzodiazepines are unclear; it may be that in the absence of clear-cut evidence of harm from benzodiazepines, clinicians will tend to favour their default practice. Definitive studies are required to better understand whether avoiding perioperative benzodiazepines has concrete benefits for patients when compared with alternative agents or nothing.

Our systematic review and meta-analysis has several strengths. To our knowledge, it is the first systematic review examining the effect that perioperative benzodiazepines have on patient-reported outcomes. We conducted our review according to a pre-published protocol and undertook a comprehensive search strategy which generated more than 33 000 references. We assessed the risk of bias of included studies, conducted prespecified subgroup analyses using the ICEMAN criteria, and evaluated the certainty of the pooled body of evidence for each outcome using the GRADE approach. Our systematic review is limited by the available evidence; most included trials were small and from single centres and did not evaluate outcomes after hospital discharge. We used meta-regression to evaluate for differences in effect that could be explained by differences in population or intervention delivery. Although we did not identify subgroup effects that were of moderate or high credibility, some analyses suggested possible differences in effect among some prespecified subgroups. Although interesting, because of the small number of studies available for meta-regression and the possibility of aggregation bias comparing effect estimates across studies, these results need to interpreted with caution. Finally, because our research question was so broad, many trials evaluated the effect of benzodiazepines in relation to active comparators, some of which could affect our outcomes of interest. Despite these limitations, we did not identify a statistically significant interaction when examining trials comparing benzodiazepines with placebo or nothing as opposed to an active comparator.

### Conclusions

Benzodiazepines in the perioperative period probably have no effect on postoperative pain or quality of recovery (moderate certainty evidence) and have an uncertain effect on patient satisfaction (very low certainty evidence). When compared with placebo or another agent, benzodiazepines might result in higher levels of postoperative anxiety (low certainty evidence). Definitive research is required to determine whether the balance of risks and benefits justifies using benzodiazepines during the perioperative period.

## Authors’ contributions

Acquisition of data: FZG, KZ, EA, RF, EBC, JY, EW, JS

Analysis and interpretation of data: FZG, KZ, EA, BS, EBC, CDM, MV, JS

Drafting of final manuscript: FZG, EW, JS

Editing of final manuscript: KZ, EA, BS, RF, EBC, JY, CB, SK, CDM, EJ, MV, JS

Study conception and design: BS, EBC, JY, EW, CB, SK, CDM, EJ, JS

## Data availability statement

Data available upon request through contact with the corresponding author.

## Funding

McMaster University, Department of Medicine (Clive Kearon Career Award to EBC); Fonds de la Recherche du Québec–Santé (Clinician-Scientist Award to MV); Heart and Stroke Foundation (National New Investigator Award to JS).

## Declarations of interest

EBC has received research grants from Bayer, BMS-Pfizer, Roche Diagnostics, and Abbott Laboratories, and consultant fees from Trimedic, all outside of the submitted work. JS has received research support from AOP Pharma and consultant fees from Trimedic and VarmX outside of the submitted work.
