# Supplementary material for: Effects of perioperative benzodiazepine administration on postoperative patient-reported outcomes: a systematic review and meta-analysis of randomised controlled trials
Source: Br J Anaesth. 2025 Sep 30;135(6):1741–52. doi: 10.1016/j.bja.2025.09.013 (PMC12799406; doi:10.1016/j.bja.2025.09.013)
Supplement: Multimedia component 1 [file mmc1.pdf]

EBSCOhost

#	Query	Limiters/Expanders	Last Run Via	Results
S40	S26 AND S39	Search modes - Proximity	Interface - EBSCOhost Research Databases<br>Search Screen - Advanced Search<br>Database - CINAHL	Display
S39	S27 OR S28 OR S29 OR S30 OR S31 OR S32 OR S33 OR S34 OR S35 OR S36 OR S37 OR S38	Search modes - Proximity	Interface - EBSCOhost Research Databases<br>Search Screen - Advanced Search<br>Database - CINAHL	Display
S38	AB surg*	Search modes - Proximity	Interface - EBSCOhost Research Databases<br>Search Screen - Advanced Search<br>Database - CINAHL	Display
S37	TI surg*	Search modes - Proximity	Interface - EBSCOhost Research Databases<br>Search Screen - Advanced Search<br>Database - CINAHL	Display
S36	TX postsurg*	Search modes - Proximity	Interface - EBSCOhost Research Databases<br>Search Screen - Advanced Search<br>Database - CINAHL	Display
S35	TX presurg*	Search modes - Proximity	Interface - EBSCOhost Research Databases<br>Search Screen - Advanced Search<br>Database - CINAHL	Display
S34	TX "intra operative"	Search modes - Proximity	Interface - EBSCOhost Research Databases<br>Search Screen - Advanced	Display

			Search<br>Database - CINAHL	
S33	TX intraoperative	Search modes - Proximity	Interface - EBSCOhost Research Databases<br>Search Screen - Advanced Search<br>Database - CINAHL	Display
S32	TX "peri operative"	Search modes - Proximity	Interface - EBSCOhost Research Databases<br>Search Screen - Advanced Search<br>Database - CINAHL	Display
S31	TX perioperative	Search modes - Proximity	Interface - EBSCOhost Research Databases<br>Search Screen - Advanced Search<br>Database - CINAHL	Display
S30	TX "post operative"	Search modes - Proximity	Interface - EBSCOhost Research Databases<br>Search Screen - Advanced Search<br>Database - CINAHL	Display
S29	TX postoperative	Search modes - Proximity	Interface - EBSCOhost Research Databases<br>Search Screen - Advanced Search<br>Database - CINAHL	Display
S28	(MH "Postoperative Complications+")	Search modes - Proximity	Interface - EBSCOhost Research Databases<br>Search Screen - Advanced Search<br>Database - CINAHL	Display
S27	(MH "Surgery, Operative+")	Search modes - Proximity	Interface - EBSCOhost Research Databases<br>Search Screen - Advanced Search<br>Database - CINAHL	Display
S26	S1 OR S2 OR S3 OR S4 OR S5 OR S6 OR S7 OR	Search modes - Proximity	Interface - EBSCOhost Research Databases	Display

	S8 OR S9 OR S10 OR S11 OR S12 OR S13 OR S14 OR S15 OR S16 OR S17 OR S18 OR S19 OR S20 OR S21 OR S22 OR S23 OR S24 OR S25		Search Screen - Advanced Search<br>Database - CINAHL	
S25	TX Triazolam*	Search modes - Proximity	Interface - EBSCOhost Research Databases<br>Search Screen - Advanced Search<br>Database - CINAHL	Display
S24	TX Midazolam*	Search modes - Proximity	Interface - EBSCOhost Research Databases<br>Search Screen - Advanced Search<br>Database - CINAHL	Display
S23	TX Medazepam*	Search modes - Proximity	Interface - EBSCOhost Research Databases<br>Search Screen - Advanced Search<br>Database - CINAHL	Display
S22	TX Estazolam*	Search modes - Proximity	Interface - EBSCOhost Research Databases<br>Search Screen - Advanced Search<br>Database - CINAHL	Display
S21	TX Clorazepate Dipotassium*	Search modes - Proximity	Interface - EBSCOhost Research Databases<br>Search Screen - Advanced Search<br>Database - CINAHL	Display
S20	TX Clobazam*	Search modes - Proximity	Interface - EBSCOhost Research Databases<br>Search Screen - Advanced Search<br>Database - CINAHL	Display
S19	TX Chlordiazepoxide*	Search modes - Proximity	Interface - EBSCOhost Research Databases<br>Search Screen - Advanced	Display

			Search<br>Database - CINAHL	
S18	TX Temazepam*	Search modes - Proximity	Interface - EBSCOhost Research Databases<br>Search Screen - Advanced Search<br>Database - CINAHL	Display
S17	TX Prazepam*	Search modes - Proximity	Interface - EBSCOhost Research Databases<br>Search Screen - Advanced Search<br>Database - CINAHL	Display
S16	TX Pirenzepine*	Search modes - Proximity	Interface - EBSCOhost Research Databases<br>Search Screen - Advanced Search<br>Database - CINAHL	Display
S15	TX Oxazepam*	Search modes - Proximity	Interface - EBSCOhost Research Databases<br>Search Screen - Advanced Search<br>Database - CINAHL	Display
S14	TX Nitrazepam*	Search modes - Proximity	Interface - EBSCOhost Research Databases<br>Search Screen - Advanced Search<br>Database - CINAHL	Display
S13	TX Lorazepam*	Search modes - Proximity	Interface - EBSCOhost Research Databases<br>Search Screen - Advanced Search<br>Database - CINAHL	Display
S12	TX Flurazepam*	Search modes - Proximity	Interface - EBSCOhost Research Databases<br>Search Screen - Advanced Search<br>Database - CINAHL	Display
S11	TX Flunitrazepam*	Search modes - Proximity	Interface - EBSCOhost Research Databases	Display

			Search Screen - Advanced Search<br>Database - CINAHL	
S10	TX Flumazenil*	Search modes - Proximity	Interface - EBSCOhost Research Databases<br>Search Screen - Advanced Search<br>Database - CINAHL	Display
S9	TX Nordazepam*	Search modes - Proximity	Interface - EBSCOhost Research Databases<br>Search Screen - Advanced Search<br>Database - CINAHL	Display
S8	TX Diazepam*	Search modes - Proximity	Interface - EBSCOhost Research Databases<br>Search Screen - Advanced Search<br>Database - CINAHL	Display
S7	TX Devazepide*	Search modes - Proximity	Interface - EBSCOhost Research Databases<br>Search Screen - Advanced Search<br>Database - CINAHL	Display
S6	TX Clonazepam*	Search modes - Proximity	Interface - EBSCOhost Research Databases<br>Search Screen - Advanced Search<br>Database - CINAHL	Display
S5	TX Bromazepam*	Search modes - Proximity	Interface - EBSCOhost Research Databases<br>Search Screen - Advanced Search<br>Database - CINAHL	Display
S4	TX Anthramycin*	Search modes - Proximity	Interface - EBSCOhost Research Databases<br>Search Screen - Advanced Search<br>Database - CINAHL	Display

S3	TX Alprazolam*	Search modes - Proximity	Interface - EBSCOhost Research Databases<br>Search Screen - Advanced Search<br>Database - CINAHL	Display
S2	TX benzodiazepine*	Search modes - Proximity	Interface - EBSCOhost Research Databases<br>Search Screen - Advanced Search<br>Database - CINAHL	Display
S1	(MH "Antianxiety Agents, Benzodiazepine+")	Search modes - Proximity	Interface - EBSCOhost Research Databases<br>Search Screen - Advanced Search<br>Database - CINAHL	Display

Web of Science Search Strategy (v0.1)

Database: Web of Science Core Collection

Entitlements:

- WOS.IC: 1993 to 2025
- WOS.CCR: 1985 to 2025
- WOS.SCI: 1900 to 2025
- WOS.AHCI: 1975 to 2025
- WOS.BHCI: 2005 to 2025
- WOS.BSCI: 2005 to 2025
- WOS.ESCI: 2005 to 2025
- WOS.ISTP: 1990 to 2025
- WOS.SSCI: 1900 to 2025
- WOS.ISSHP: 1990 to 2025

Searches:

1: TS=((Benzodiazepine* OR Alprazolam* OR Benzodiazepinone* OR Anthramycin* OR Bromazepam* OR Clonazepam* OR Devazepide* OR Diazepam* OR Nordazepam* OR Flunitrazepam* OR Flumazenil* OR Flurazepam* OR Lorazepam* OR Nitrazepam* OR Oxazepam* OR Pirenzepine* OR Prazepam* OR Temazepam* OR Chlordiazepoxide* OR Clobazam* OR "Clorazepate Dipotassium*" OR Estazolam* OR Medazepam* OR Midazolam* OR Triazolam*)) AND TS=((surger* OR surgical* OR surgeon* OR intraoperative OR "intra-operative" OR postoperative OR "post-operative" OR perioperative OR "peri-operative" OR presurg* OR postsurg*)) NOT TS=((child* OR infant* OR neonat*) NOT adult*) Editions: WOS.SCI,WOS.SSCI,WOS.AHCI,WOS.ISTP,WOS.ISSHP,WOS.ESCI Timespan: All years (Publication Date) Results: 7151

Database: EBM Reviews - Cochrane Central Register of Controlled Trials <February 2025>

Search Strategy:

1 exp Benzodiazepines/ (11622)

2 benzodiazepine*.mp. (7063)

3 alprazolam*.mp. (1189)

4 benzodiazepinone*.mp. (358)

5 Anthramycin*.mp. (0)

6 Bromazepam*.mp. (249)

7 Clonazepam*.mp. (567)

8 Devazepide*.mp. (5)

9 Diazepam*.mp. (4185)

10 Nordazepam*.mp. (87)

11 Flunitrazepam*.mp. (535)

12 Flumazenil*.mp. (674)

13 Flurazepam*.mp. (271)

14 Lorazepam*.mp. (2084)

15 Nitrazepam*.mp. (329)

16 Oxazepam*.mp. (383)

17 Pirenzepine*.mp. (679)

18 Prazepam*.mp. (68)

19 Temazepam*.mp. (442)

20 Chlordiazepoxide*.mp. (463)

21 Clobazam*.mp. (357)

22 Clorazepate Dipotassium*.mp. (112)

23 Estazolam*.mp. (182)

24 Medazepam*.mp. (59)

25 Midazolam*.mp. (10657)

26 Triazolam*.mp. (647)

27 or/1-26 [Benzodiazepines] (25446)

28 exp Surgical Procedures, Operative/ (169871)

29 exp Monitoring, Intraoperative/ (1814)

30 exp Postoperative Complications/ (55011)

31 intraoperative.mp. (35833)

32 postoperative.mp. (156944)

33 post operative.mp. (30690)

34 perioperative.mp. (27017)

35 peri operative.mp. (2754)

36 intra operative.mp. (4799)

37 surg*.tw,kf. (284792)

38 surgery.fs. (87116)

39 presurg*.mp. (1089)

40 postsurg*.mp. (3593)

41 or/28-40 [Surgery] (423582)

42 27 and 41 (8539)

43 exp animals/ not humans.mp. [mp=title, original title, abstract, floating sub-heading word, mesh headings, heading words, keyword] (3640)

44 (exp Child/ or exp Adolescent/ or exp Infant/) not exp Adult/ (86024)

45 42 not (43 or 44) (7765)

Database: Embase <1974 to 2025 April 02>

Search Strategy:

1 benzodiazepine*.tw,kw. (59369)
2 alprazolam*.tw,kw. (4088)
3 benzodiazepinone*.tw,kw. (139)
4 Anthramycin*.tw,kw. (173)
5 Bromazepam*.tw,kw. (921)
6 Clonazepam*.tw,kw. (6921)
7 Devazepide*.tw,kw. (407)
8 Diazepam*.tw,kw. (27957)
9 Nordazepam*.tw,kw. (108)
10 Flunitrazepam*.tw,kw. (3763)
11 Flumazenil*.tw,kw. (4649)
12 Flurazepam*.tw,kw. (1181)
13 Lorazepam*.tw,kw. (7911)
14 Nitrazepam*.tw,kw. (1301)
15 Oxazepam*.tw,kw. (1964)
16 Pirenzepine*.tw,kw. (3531)
17 Prazepam*.tw,kw. (308)
18 Temazepam*.tw,kw. (1320)
19 Chlordiazepoxide*.tw,kw. (3114)
20 Clobazam*.tw,kw. (2317)
21 Clorazepate Dipotassium*.tw,kw. (101)
22 Estazolam*.tw,kw. (432)
23 Medazepam*.tw,kw. (250)
24 Midazolam*.tw,kw. (25203)
25 Triazolam*.tw,kw. (2062)
26 or/1-25 [Benzodiazepines] (122778)
27 exp surgery/ (6440184)
28 surgery.fs. (2489325)
29 surg*.tw,kw. (3451385)
30 intraoperative.tw,kw. (242459)
31 intra operative.tw,kw. (33054)
32 postoperative.tw,kw. (865508)
33 post operative.tw,kw. (171519)
34 perioperative.tw,kw. (200575)

35 peri operative.tw,kw. (20967)

36 presurg*.tw,kw. (18198)

37 postsurg*.tw,kw. (33942)

38 or/27-37 [Surgery] (7632719)

39 26 and 38 (29144)

40 exp Juvenile/ not exp Adult/ (2737310)

41 exp Animal/ not exp Human/ (5741379)

42 39 not (40 or 41) (22604)

43 (Randomized controlled trial/ or Controlled clinical study/ or random$.ti,ab. or randomization/ or intermethod comparison/ or placebo.ti,ab. or (compare or compared or comparison).ti. or ((evaluated or evaluate or evaluating or assessed or assess) and (compare or compared or comparing or comparison)).ab. or (open adj label).ti,ab. or ((double or single or doubly or singly) adj (blind or blinded or blindly)).ti,ab. or double blind procedure/ or parallel group$1.ti,ab. or (crossover or cross over).ti,ab. or ((assign$ or match or matched or allocation) adj5 (alternate or group$1 or intervention$1 or patient$1 or subject$1 or participant$1)).ti,ab. or (assigned or allocated).ti,ab. or (controlled adj7 (study or design or trial)).ti,ab. or (volunteer or volunteers).ti,ab. or human experiment/ or trial.ti.) not (((random$ adj sampl$ adj7 ("cross section$" or questionnaire$1 or survey$ or database$1)).ti,ab. not (comparative study/ or controlled study/ or randomi?ed controlled.ti,ab. or randomly assigned.ti,ab.)) or (Cross-sectional study/ not (randomized controlled trial/ or controlled clinical study/ or controlled study/ or randomi?ed controlled.ti,ab. or control group$1.ti,ab.)) or (((case adj control$) and random$) not randomi?ed controlled).ti,ab. or (Systematic review not (trial or study)).ti. or (nonrandom$ not random$).ti,ab. or "Random field$".ti,ab. or (random cluster adj3 sampl$).ti,ab. or ((review.ab. and review.pt.) not trial.ti.) or ("we searched".ab. and (review.ti. or review.pt.)) or "update review".ab. or (databases adj4 searched).ab. or ((rat or rats or mouse or mice or swine or porcine or murine or sheep or lambs or pigs or piglets or rabbit or rabbits or cat or cats or dog or dogs or cattle or bovine or monkey or monkeys or trout or marmoset$1).ti. and animal experiment/) or (Animal experiment/ not (human experiment/ or human/))) (6089542)

44 42 and 43 (8196)

Database: APA PsycInfo <1806 to March 2025 Week 4>

Search Strategy:

1 exp Benzodiazepines/ (11759)

2 benzodiazepine*.mp. (18114)

3 alprazolam*.mp. (1485)

4 Bromazepam*.mp. (160)

5 Clonazepam*.mp. (1537)

6 Devazepide*.mp. (123)

7 Diazepam*.mp. (5618)

8 Nordazepam*.mp. (39)

9 Flunitrazepam*.mp. (518)

10 Flumazenil*.mp. (1169)

11 Flurazepam*.mp. (307)

12 Lorazepam*.mp. (2108)

13 Nitrazepam*.mp. (227)

14 Oxazepam*.mp. (409)

15 Pirenzepine*.mp. (1163)

16 Prazepam*.mp. (40)

17 Temazepam*.mp. (242)

18 Chlordiazepoxide*.mp. (1902)

19 Clobazam*.mp. (284)

20 Clorazepate Dipotassium*.mp. (71)

21 Estazolam*.mp. (38)

22 Medazepam*.mp. (52)

23 Midazolam*.mp. (1349)

24 Triazolam*.mp. (582)

25 or/1-24 [Benzodiazepines] (27445)

26 exp Surgery/ (59186)

27 exp Postsurgical Complications/ (1490)

28 intraoperative.mp. (1584)

29 postoperative.mp. (12535)

30 post operative.mp. (2118)

31 perioperative.mp. (1900)

32 peri operative.mp. (125)

33 intra operative.mp. (200)

34 [surg*.tw,kf.] (0)

35 presurg*.mp. (1906)

36 postsurg*.mp. (3407)

37 exp surgery/ (59186)

38 postsurgical complications/ (1490)

39 or/26-38 [Surgery] (67196)

40 25 and 39 (368)
