# Supplementary material for: Effects of perioperative benzodiazepine administration on postoperative patient-reported outcomes: a systematic review and meta-analysis of randomised controlled trials
Source: Br J Anaesth. 2025 Sep 30;135(6):1741–52. doi: 10.1016/j.bja.2025.09.013 (PMC12799406; doi:10.1016/j.bja.2025.09.013)
Supplement: Multimedia component 2 [file mmc2.docx]

**Appendix 2: Prespecified subgroups and associated hypotheses to explain heterogeneity**

| Subgroup | Hypothesized effect on patient-reported outcomes |
| --- | --- |
| Studies including females only compared to mixed-sex studies | Studies including females only will report more favourable effects on patient-reported outcomes |
| Studies of younger (< 65 years) patients compared to elderly (≥65 years) patients | Studies of younger (< 65 years) patients compared to elderly (≥65 years) patients will report more favourable effects on patient-reported outcomes |
| Studies comparing benzodiazepines to an active comparator versus those comparing benzodiazepines to placebo or nothing | Studies comparing benzodiazepines to an active comparator versus those comparing benzodiazepines to placebo or nothing will report less favourable effects on patient-reported outcomes |
| Studies using remimazolam compared to studies using other benzodiazepines | Studies using remimazolam compared to studies using other benzodiazepines will report less favourable effects on patient-reported outcomes |
| High compared to low risk of bias studies | Low risk of bias studies will report more favourable effects on patient-reported outcomes |
