# Supplementary material for: Effects of perioperative benzodiazepine administration on postoperative patient-reported outcomes: a systematic review and meta-analysis of randomised controlled trials
Source: Br J Anaesth. 2025 Sep 30;135(6):1741–52. doi: 10.1016/j.bja.2025.09.013 (PMC12799406; doi:10.1016/j.bja.2025.09.013)
Supplement: Multimedia component 3 [file mmc3.docx]

**Appendix 3: Details of included studies**

| **Author** | **Year** | **Study Size** | **Benzodiazepine Arm** | **Comparator Arm** | **Surgery** | **Age (years)** | **Outcomes reported** | **Data synthesis** |
| --- | --- | --- | --- | --- | --- | --- | --- | --- |
| Aanta^1^ | 1991 | 107; 36//36/35 | Midazolam 0.08 mg/kg IM preoperatively | Saline preoperatively; Dexmedetomidine 1 mg/kg IM preoperatively | D&C | Mean 46 (SD 6) | Pain | Qualitative synthesis.  VAS data not reported. |
| Abdelemam^2^ | 2022 | 84; 28/28//28 | Midazolam 5 mg + bupivacaine 0.25% intrathecal intraoperatively; Morphine 5 mg + midazolam 5 mg + bupivacaine 0.25% intrathecal intraoperatively | Morphine 5 mg + bupivacaine 0.25% intrathecal intraoperatively | Abdominal cancer surgery | Median 58  (IQR 43-67) | Satisfaction | Quantitative synthesis. |
| Abdellatif^3^ | 2012 | 54; 27//27 | Midazolam 0.01–0.03 mg/kg IV bolus, followed by infusion at 0.04 mg/kg/h intraoperatively | Dexmedetomidine 1 µg/kg IV bolus, followed by infusion at 0.4 µg/kg/h (range: 0.1–1 µg/kg/h) intraoperatively | Simple middle ear surgeries | Mean 32 (SD 11) | Satisfaction | Qualitative synthesis. Ordinal scale used. |
| Abdelrady^4^ | 2024 | 85; 42//43 | Midazolam 2 mg + 0.5% isobaric levobupivacaine 10 mg intrathecal intraoperatively | Fentanyl 25 µg + and 0.5% levobupivacaine 10 mg intrathecal intraoperatively | Elective C-section | Mean 28 (SD 6) | Pain; Satisfaction | Qualitative synthesis. No standard deviation reported. |
| Abhishek^5^ | 2022 | 80; 40//40 | Midazolam 0.06 mg/kg + fentanyl 1 μg/kg IV infusion intraoperatively | Dexmedetomidine 1 µg/kg IV infusion intraoperatively | Various middle ear surgeries | Mean 32 (SD 10) | Satisfaction | Quantitative synthesis. |
| Aldrich^6^ | 2022 | 123; 60//63 | Diazepam 10 mg intrarectal postoperatively | Placebo intrarectal postoperatively | Vaginal hysterectomy | Median 65 (IQR 27-80) | Pain; Satisfaction | Quantitative synthesis. |
| Aleniewski^7^ | 1977 | 40; 40//40 | Lorazepam 4 mg IM preoperative | Pentobarbital 100 mg IM preoperative | Not reported | Mean 45 (SD 14) | Satisfaction | Quantitative synthesis. |
| Amin^8^ | 2022 | 120; 40/40//40 | Midazolam 2 mg IV preoperatively; Midazolam 2 mg IV intraoperatively | Normal saline IV pre- and intraoperatively | Laparoscopic gynecologic surgery | Mean 37 (SD 14) | Pain | Quantitative synthesis. |
| Amin^9^ | 2019 | 90; 30//30/30 | Midazolam 2.5 mg + hyperbaric bupivacaine 12.5 mg intrathecal intraoperatively | Hyperbaric bupivacaine intrathecal 12.5 mg + 0.5 mL saline intraoperatively; Hyperbaric bupivacaine intrathecal 12.5 mg + nalbuphine 1 mg intrathecal intraoperatively | C-section | Mean 26 (SD 4) | Pain | Quantitative synthesis. |
| Ammar^10^ | 2018 | 60; 30//30 | 20 mL of bupivacaine hydrochloride 0.25% + midazolam 50 µg/kg in 2 ml saline 0.9% intraoperative block | Placebo 2 mL saline, intraoperative block | Umbilical or epigastric hernia repair | Mean 32 (SD 8) | Pain | Quantitative synthesis. |
| Anand^11^ | 2017 | 60; 30//30 | Alprazolam 0.5 mg PO 2h preoperatively | Pregabalin 150 mg PO 2h preoperatively | Laparoscopic cholecystectomy | Mean 35 (SD 8) | Pain; Satisfaction | Quantitative synthesis. |
| Arifin^12^ | 2023 | 30; 15//15 | Midazolam 0.02 mg/kg IV preoperatively | VR (oculus quest) intraoperatively | Lower abdominal or lower extremity | Mean 33 (SD 9) | Anxiety; Satisfaction | Quantitative synthesis. |
| Ashok^13^ | 2024 | 80; 40//40 | Midazolam IV 0.02-0.04 mg/kg in 10 ml of  normal saline slowly, followed by maintenance  infusion of 0.02-0.04 mg/kg/h intraoperatively | Dexmedetomidine IV loading dose of 0.5 µg/kg over 10 min, followed  by maintenance infusion of 0.2-0.6 µg/kg/h intraoperatively | Upper limb surgeries | Mean 47 (SD) | Pain | Quantitative synthesis. |
| Bagchi^14^ | 2014 | 103; 51//51 | Midazolam bolus 0.05 mg/kg, followed by infusion at 0.06 mg/kg/h intraoperatively | Propofol bolus 1 mg/kg, followed by infusion at 3 mg/kg/h IV intraoperatively | Elective infraumbilical surgeries | Mean 37 (SD 11) | Satisfaction | Qualitative synthesis. Satisfaction assessed using 7-point likert scale. |
| Basuni^15^ | 2016 | 50; 25//25 | Ketamine 10 mg, midazolam 2 mg and 0.5% hyperbaric bupivacaine 8 mg intrathecal intraoperatively | Fentanyl 25 μg and 0.5% hyperbaric bupivacaine 8 mg intrathecal intraoperatively | C-section | Mean 29 (SD 4) | Satisfaction | Quantitative synthesis. |
| Bindra^16^ | 2010 | 80; 40//40 | Alprazolam 0.5 mg PO preoperatively | Placebo PO preoperatively | Hysterectomy | Mean 44 (SD 6) | Pain; Anxiety; Satisfaction | Quantitative synthesis. |
| Bishnoi^17^ | 1998 | 52; 26//26 | Fentanyl 0.5 mg/kg and midazolam 0.03 mg/kg over 10 minutes followed by continuous infusion of 0.5-1.16 mg/kg/h fentanyl and 0.03-0.07 mg/kg/h midazolam IV intraoperatively | Dexmedetomidine 1 mg/kg over 10 minutes followed by continuous infusion 0.03-0.07 mg/kg/h IV intraoperatively | Arthroscopic meniscectomy | Mean 58 (SD 13) | Satisfaction | Quantitative synthesis. |
| Bond^18^ | 1976 | 60; 30//30 | Droperidol 0.1 mg/kg and fentanyl 0.005 mg/kg IV intraoperatively | Diazepam 0.2 mg/kg and pentazocine 0.5 mg/kg IV intraoperatively | Mix of mainly general, gynecologic, and orthopedic procedures | Mean 29 (SD not reported) | Pain | Quantitative synthesis. |
| Borracci^19^ | 2013 | 60; 28//32 | Midazolam 0.05 mg/kg PO preoperatively | Morphine 0.5 mg/kg PO preoperatively | Major abdominal surgery | Mean 67 (SD 10) | Pain | Quantitative synthesis. |
| Caumo^20^ | 2002 | 112; 56//56 | Diazepam 10 mg PO 1h preoperatively | Placebo PO 1h preoperatively | Abdominal hysterectomy | Mean 44 (SD 7) | Pain; Anxiety; Satisfaction | Quantitative synthesis. |
| Chandra^21^ | 2024 | 60; 30//30 | Diazepam 10 mg PO preoperatively | Pregabalin 150 mg PO preoperatively | Infraumbilical surgeries | Mean 31 (SD 7) | Pain | Quantitative synthesis. |
| Chen^22^ | 2024 | 80; 40//40 | Remimazolam initial bolus of 0.03 mg/kg/min, then repeated doses of 0.2–0.5 mg/kg/h IV intraoperatively | Dexmedetomidine initial bolus of 0.3 µg/kg, then additional doses of 0.2–1.0 µg/kg/h IV intraoperatively | Orthopedic surgery | Mean 59 (SD 11) | Satisfaction | Qualitative synthesis. Ordinal scale used. |
| Choi^23^ | 2021 | 88; 44//44 | Midazolam 0.05 mg/kg IV intraoperatively | Saline IV intraoperatively | Laparoscopic cholecystectomy | Mean 49 (SD 11) | Pain | Quantitative synthesis. |
| Choi^24^ | 2022 | 139; 70//69 | Remimazolam infusion at 6 mg/kg/h for induction and 1–2 mg/kg/h for maintenance IV intraoperatively | Propofol target-controlled infusion with an effect-site concentration of 5 μg/mL for induction and 2–6 μg/mL IV intraoperatively | Thyroidectomy | Median 40 (IQR 35-48) | QoR | Quantitative synthesis. |
| Dash^25^ | 2024 | 300; 150//150 | Midazolam 0.05 mg/kg PO preoperatively | Placebo (vitamin C) PO preoperatively | Elective surgeries | Mean 45 (SD 13) | Pain; Anxiety | Quantitative synthesis. |
| Dyck^26^ | 1991 | 92; 31//31/30 | Diazepam 10 mg PO preoperatively | Propranolol 80 mg PO preoperatively; Placebo | D&C | Mean 24 (SD 5) | Anxiety | Quantitative synthesis. |
| El-Baradey^27^ | 2014 | 60; 20//20/20 | 30 mL bupivacaine 0.5% + midazolam 50 μg/kg intraoperative block | 30 mL bupivacaine 0.5% perineural intraoperatively; 30 mL bupivacaine 0.5% + dexamethasone 8 mg intraoperative block | Elective upper limb surgery | Mean 27 (SD 8) | Pain; Satisfaction | Quantitative synthesis. |
| El-Deeb^28^ | 2011 | 120; 40//40/40 | Midazolam 2 mg + ondansetron 4 mg IV intraoperatively | Saline placebo; Dexamethasone 8 mg + ondansetron 4 mg intraoperatively | Elective thyroid | Mean 30.4 (SD 3) | Pain; Satisfaction | Quantitative synthesis. |
| Ellingson^29^ | 1977 | 26; 13//13 | 30 mg diazepam IV intraoperatively | Ketamine 2 mg/kg IV intraoperatively | Obstetric | Mean 25 (SD not reported) | QoR | Qualitative synthesis. Dichotomous data reported. |
| Elvir Lazo^30^ | 2016 | 120; 40//40/40 | Midazolam 2 mg IV preoperatively | Saline; Propofol 20 mg IV preoperatively | Orthopedic surgery | Mean 51 (SD 14) | Anxiety | Quantitative synthesis. |
| Ersoy^31^ | 2015 | 60; 30//30 | Midazolam 0.05 mg/kg bolus followed by 0.02-0.1 mg/kg/h infusion IV intraoperatively | Propofol 1 mg/kg bolus followed by 1-3 mg/kg/h infusion IV intraoperatively | Hip surgery | Mean 80 (SD 7) | Satisfaction | Quantitative synthesis. |
| Feng^32^ | 2019 | 248; 124//124 | 0.5% levobupivacaine preoperatively + midazolam 1.5 mg in normal saline IV intraoperatively | Propofol 1 mg/kg bolus + 0.5 mg/kg/min IV intraoperatively | Minimally invasive parathyroidectomy | Mean 60 (SD 4) | Pain | Quantitative synthesis. |
| Fragen^33^ | 1976 | 95; 32/32//31 | Lorazepam 4 mg IM preoperatively; Diazepam 10 mg IM preoperatively | Placebo | Not reported | Mean 43 (SD not reported) | Satisfaction | Qualitative synthesis. Ordinal data reported. |
| Franssen^34^ | 1993 | 68; 34//34 | Alprazolam 1 mg PO preoperatively | Hydroxyzine 75 mg PO preoperatively | Plastic or orthopaedic surgery | Mean 39 (SD 39) | Anxiety, Satisfaction | Qualitative synthesis. Results described narratively. |
| Fredman^35^ | 1999 | 89; 29/30//30 | Midazolam 0.5 mg IV preoperatively; Midazolam 2 mg IV preoperatively | Placebo | Transurethral procedures | Mean 73 (SD 6) | Anxiety; Satisfaction | Qualitative synthesis. Results described narratively. |
| Gale^36^ | 1976 | 66; 33//33 | Lorazepam 'weight-related' dose IM preoperatively | Pantopon 'weight-related' dose IM preoperatively | D&C | Mean 28 (SD 10) | Anxiety | Quantitative synthesis. |
| Ganguly^37^ | 2017 | 64; 32//32 | Midazolam 0.05 mg/kg loading dose followed by maintenance with 0.05 mg/kg/h IV intraoperatively | Dexmedetomidine 0.5 μg/kg loading dose followed by maintenance with 0.5 μg/kg/h IV intraoperatively | Infraumbilical surgeries | Mean 37 (SD 12) | Satisfaction | Qualitative synthesis. Satisfaction assessed using 7-point likert scale. |
| Gao^38^ | 2022 | 50; 25//25 | Midazolam 0.1 mg/kg bolus preoperatively + dexmedetomidine 1 µg/kg over a 10 min and 0.5 µg/kg/h IV intraoperatively | Dexmedetomidine 1 µg/kg over 15 min preoperatively + dexmedetomidine 0.2-1 µg/kg/h IV + propofol 250 μg/kg/min for 15 min then a basal infusion dose of 50 μg/kg/min intraoperatively | Major abdominal surgery | Mean 47 (7.4) | Pain | Qualitative synthesis. Dichotomous data reported. |
| Gilliland^39^ | 1996 | 45; 23//22 | Induction dose of midazolam 5 mg/70 kg followed after 60 minutes by an IV loading dose infusion of midazolam at 1 mg/70 kg/h over the next 48h | Placebo | Elective abdominal hysterectomy | Mean 42 (SD 10) | Pain | Qualitative synthesis. Ordinal data reported. |
| Giordano^40^ | 2023 | 70; 35//36 | Midazolam 0.02 mg/kg IV preoperatively | Music | Stomatology surgery | Mean 61 (SD 12) | Anxiety; Satisfaction | Quantitative synthesis. |
| Ha^41^ | 2007 | 119; 41//39/39 | Midazolam 0.075 mg/kg IV intraoperatively | Ondansetron 4 mg IV; normal saline 10 ml IV | Thyroidectomy under general anesthesia | Mean 44 (SD 12) | Pain | Quantitative synthesis. |
| Haram^42^ | 1981 | 82; 39//43 | Diazepam 0.3 mg/kg IV intraoperatively | Thiopentone 3 mg/kg IV intraoperatively | C-section | Mean 31 (SD 6) | QoR | Qualitative synthesis. Ordinal data reported. |
| Hashemian^43^ | 2023 | 220; 110//110 | Midazolam 1-2 mg IV intraoperatively | Propofol 25–75 g/kg/min IV intraoperatively | Traumatic lower limb surgery | Mean 26 (SD 6) | Anxiety | Quantitative synthesis. |
| He^44^ | 2024 | 120; 40//40/40 | Alprazolam 0.4 mg PO preoperatively | Dexmedetomidine 1.5 ug/kg intranasal  drip; Placebo (saline nasal drops) | Laparoscopic elective gynecologic surgery | Mean 69 (SD 3) | Anxiety | Quantitative synthesis. |
| Honarmand^45^ | 2012 | 90; 20/30//20/20 | Midazolam 2 mg IV intraoperatively; Midazolam 2 mg + haloperidol 2 mg IV intraoperatively | Haloperidol 2 mg IV intraoperatively; Saline IV intraoperatively | Mastoidectomy or tympanoplasty or both | Mean 35 (SD 14) | Pain | Quantitative synthesis. |
| Hong^46^ | 2021 | 104; 52//52 | Midazolam 4 mg + palonosetron 0.075 mg + fentanyl 20 μg/kg IV intraoperatively | Palonosetron 0.075 mg + fentanyl 20 μg/kg IV intraoperatively | Breast cancer surgery | Mean 47 (SD 7) | Pain | Quantitative synthesis. |
| Hong^47^ | 2024 | 70; 35//35 | Remimazolam 6 mg/kg/h for 10 min + 1 mg/kg/h IV intraoperatively | Dexmedetomidine 6 µg/kg/h for 10 min + 1 µg/kg/h intraoperatively | Orthopaedic surgery | Mean 39 (SD 14) | Pain | Quantitative synthesis. |
| Hu^48^ | 2020 | 105; 35//35/35 | Midazolam 0.02 mg/kg IV intraoperatively | Dexmedetomidine 1 µg/kg IV intraoperatively; Saline IV intraoperatively | C-section | Mean 33 (SD 4) | Satisfaction | Qualitative synthesis. Ordinal data reported. |
| Ibrahim^49^ | 2001 | 141; 48//93 | Midazolam infusion, titrated to 'the desired effect' | Sevoflurane titrated to a MAC of 1.0 | Not reported | Mean 48 (SD 15) | Satisfaction; QoR | Qualitative synthesis. Satisfaction reported as dichotomous. QoR reported as ordinal. |
| Ionescu^50^ | 2008 | 53; 17//18/18 | Midazolam 2.75 mg PO preoperatively | Melatonin 3 mg PO preoperatively | Laparoscopic cholecystectomy | Mean 47 (SD 11) | Anxiety | Quantitative synthesis. |
| Jabalameli^51^ | 2012 | 132; 44/44//44 | Midazolam 30 µg/kg IV postoperatively; Midazolam 30 µg/kg + ondansetron 8 mg IV postoperatively | Ondansetron 8 mg IV postoperatively | C-section | Mean 30 (SD 7) | Pain | Quantitative synthesis. |
| Kain^52^ | 2001 | 65; 31//34 | Lorazepam 1mg PO 1 night prior + midazolam 5 mg IM 30 min preoperatively | Placebo PO 1 night prior + placebo IM IM 30 min preoperatively | Hysterectomy | Mean 45 (SD 7) | Pain; Anxiety; QoR | Quantitative synthesis. |
| Kang^53^ | 2023 | 60; 30//30 | Midazolam 0.03 mg/kg after delivery | Dexmedetomidine 0.7 μg/kg after delivery | C-section | Mean 35 (SD 4) | Pain | Quantitative synthesis. |
| Kapdi^54^ | 2021 | 100; 50//50 | Midazolam 1 mg + 0.5% bupivacaine 10 mg intrathecal intraoperatively | Nalbuphine 0.75 mg + 0.5% bupivacaine 10 mg intrathecal intraoperatively | C-section | Mean 27 (SD 4) | Pain | Qualitative synthesis. No data reported. |
| Karbasfrushan^55^ | 2012 | 124; 62//62 | Midazolam 2 mg/mL + bupivacaine 12.5 mg/2.5 ml intrathecal intraoperatively | Bupivacaine 12.5 mg/2.5 ml + normal saline 1 mL intraoperatively | C-section | Mean 32 (SD not reported) | Pain | Quantitative synthesis. |
| Keerthy^56^ | 2022 | 100; 50//50 | Alprazolam 0.5 mg/kg PO preoperatively | Melatonin 3 mg oral preoperatively | Elective surgeries under general anaesthesia | Not reported | Anxiety | Quantitative synthesis. |
| Kestin^57^ | 1990 | 39; 13/13//13 | Midazolam IV bolus in 1 mg increments until patient reached sedation level 3, and further midazolam was given to maintain sedation intraoperatively; Midazolam (as above) followed by antagonism with flumazenil intraoperatively | Propofol IV  loading dose of 0.5 mg/kg over 20s, followed immediately by an infusion of 5 mg/kg/h for 10 min, 4 mg/kg/h for 10 min and 3 mg/kg/h intraoperatively | TURP | Mean 71 (SD 6) | Anxiety | Quantitative synthesis. |
| Kim^58^ | 2001 | 45; 15/15//15 | Bupivacaine 0.5% + midazolam 1 mg intrathecal intraoperatively; Bupivacaine 0.5% + midazolam 1 mg intrathecal intraoperatively | Bupivacaine 0.5% + saline intrathecal intraoperatively | Hemorrhoidectomy | Mean 43 (SD 11) | Pain | Quantitative synthesis. |
| Kim^59^ | 2010 | 60; 30//30 | Midazolam 0.2 mg/mL + fentanyl 4 µg/mL + 0.2% ropivacaine IV infusion postoperatively | Fentanyl 4 µg/mL + 0.2% ropivacaine IV postoperatively | Subtotal gastrectomy | Mean 58 (SD 2) | Pain | Quantitative synthesis. |
| Kim^60^ | 2011 | 215; 107//108 | Midazolam 1 mg/kg/h then 0.5 mg/kg/h IV intraoperatively | Propofol 10 mg/kg/h then 5 mg/kg/h IV intraoperatively | Not reported | Mean 39 (SD 15) | Satisfaction | Quantitative synthesis. |
| Kim^61^ | 2013 | 94; 32/32//30 | Midazolam 75 µg/kg IV bolus intraoperatively; Midazolam 75 µg/kg + ramosetron 0.3 mg IV bolus intraoperatively | Ramosetron 0.3 mg IV bolus intraoperatively | Thyroidectomy | Mean 48 (SD not reported) | Pain | Qualitative synthesis. Outcomes described narratively. |
| Kim^62^ | 2017 | 81; 40//41 | Midazolam 0.02 mg/kg IV preoperatively | Normal saline IV preoperatively | Partial, total mastectomy, modified radical mastectomy | Mean 48 (SD not reported) | QoR | Quantitative synthesis. |
| Kim^63^ | 2024 | 103; 52//51 | Remimazolam 0.075 mg/kg IV infused over 1 min, followed by maintenance infusion 0.5 mg/kg/h intraoperatively | Dexmedetomidine 1µg/kg IV infused over 10 min, followed by maintenance infusion 0.5µg/kg/h intraoperatively | Lower extremity surgery | Mean 93 (SD 15) | Satisfaction | Quantitative synthesis. |
| Kotani^64^ | 2024 | 35; 17//18 | Remimazolam 12 mg/kg/min IV intraoperatively | Propofol 2.5 µg/mL IV intraoperatively | Transcatheter aortic valve  replacement | Mean 84 (SD 4) | QoR | Quantitative synthesis. |
| Kowark^65^ | 2024 | 607; 303//303 | Midazolam 3.75 mg PO preoperatively | Placebo preoperatively | Elective inpatient surgery | Mean 72 (SD 4) | Satisfaction | Quantitative synthesis. |
| Kulkarni^66^ | 2022 | 120; 60//60 | Midazolam 0.04 mg/kg IV preoperatively | Dexmedetomidine 1 µg/kg IV preoperatively | Minor obstetric and gynecological procedures | Mean 29 | Anxiety | Quantitative synthesis. |
| Kumar^67^ | 2020 | 100; 50//50 | Midazolam 0.05 mg/kg IV preoperatively | Dexmedetomidine 0.5 μg/kg IV preoperatively | Infra-umbilical surgery | Mean 40 (SD 14) | Pain | Quantitative synthesis. |
| Kwak^68^ | 2008 | 42; 21//21 | Midazolam 0.2mg/mL + fentanyl 4 μg/mL + ropivacaine infusion (PCEA) postoperatively | Fentanyl 4 μg/mL + ropivacaine infusion (PCEA) postoperatively | Subtotal gastrectomy | Mean 59 (SD 7) | Pain | Quantitative synthesis. |
| Lal^69^ | 2023 | 108; 36/36//36 | Midazolam 0.05 mg/kg intraoperatively; Midazolam 0.05 mg/kg + hand holding intraoperatively | Hand holding and conversation with 5 mL normal saline IV intraoperatively | Lumbar spine surgery | Mean 33 (SD 9) | Anxiety | Quantitative synthesis. |
| Lee^70^ | 2007 | 90; 45//45 | Midazolam 2 mg IV intraoperatively | Ondansetron 4 mg IV intraoperatively | Hysteroscopy | Mean 48 (SD 10) | Pain | Quantitative synthesis. |
| Lee^71^ | 2023 | 57; 28//29 | Remimazolam 6 mg/kg/h induction + 1–2 mg/kg/h maintenance IV intraoperatively | Propofol induced at effect-site concentration 3.0 ng/mL IV intraoperatively | Thyroidectomy | Mean 49 (SD 13) | QoR | Quantitative synthesis. |
| Lee, Cheol^72^ | 2024 | 108; 36//36/36 | Remimazolam 6 mg/kg/h loading dose  followed by 1 mg/kg/h and remifentanil 0.3 µg /kg/min IV intraoperatively | Desflurane 1 MAC  and remifentanil 0.3 µg /kg/min; Desflurane 1 MAC  and remifentanil 0.05 µg/kg/min IV intraoperatively | Laparoscopically assisted urological surgery | Mean 50 (SD not reported) | Pain | Quantitative synthesis. |
| Lee, Jaemoon^73^ | 2024 | 63; 32//31 | Remimazolam 6 mg/kg/h + remifentanil (Minto model, effect-site concentration ≤4 ng/mL) IV intraoperatively | Propofol (Modified Marsh model, effect-site concentration ≤4 µg/mL + remifentanil (Minto model, effect-site concentration ≤4.0 ng/mL) IV intraoperatively | Breast cancer  surgery | Mean 54 (SD 9) | Pain, QoR | Quantitative synthesis. |
| Lee, Jiwon^74^ | 2024 | 72; 36//36 | Glycopyrrolate 0.1 mg + remimazolam 6-12 mg/kg/h remimazolam + remifentanil 3.0 ng/mL TCI IV intraoperatively | Sevoflurane, glycopyrrolate 0.1 mg + lidocaine 40 mg + propofol 1–2 mg/kg + remifentanil 3.0 ng/mL TCI IV intraoperatively | Elective ACDF surgery | Mean 49 (Range 30-68) | Pain, QoR | Quantitative synthesis. |
| Liang^75^ | 2024 | 88; 32//28/28 | Remimazolam 0.5 mg/kg IV + 5 g of lidocaine-prilocaine cream preoperatively | Normal saline 1 mg/mL IV and 5 g of lidocaine-prilocaine cream preoperatively; Normal saline 1 mg/mL IV and moisturizing cream preoperatively | Surgery with spinal anesthesia | Mean 40 (SD not reported) | Anxiety | Quantitative synthesis. |
| Liu^76^ | 2023 | 136; 30//30/30/30/16 | Midazolam 0.03 mg/kg IV intraoperatively | Normal saline 0.1 ml/kg 15 min pre-anaesthesia 0.125 ml/kg/h maintenance; Dexmedetomidine (DEX) 0.25 μg/kg 15 min pre-anaesthesia + 0.5 μg/kg/h (maintenance dose); DEX 0.5 μg/kg 15 min pre-anaesthesia + maintenance dose; DEX 0.75 μg/kg 15 min pre-anaesthesia + maintenance dose | Hip replacement surgery | Mean 75 (SD 7) | Pain | Qualitative synthesis. Dichotomous data presented. |
| Liu^77^ | 2024 | 100; 50//50 | Induction: Sufentanil 0.1-2 μg/kg + remimazolam 0.1-0.2 mg/kg + cisatracurium 0.2 mg/kg. Maintenance: remimazolam 0.4-1.2 mg/kg/h + remifentanil 0.1 to 0.2 μg/kg/min IV intraoperatively | Induction: Sufentanil 0.1-2 μg/kg + propofol 1-2 mg/kg + cisatracurium 0.2 mg/kg. Maintenance: propofol 4-10 mg/kg/h + remifentanil 0.1-0.2 μg/kg/min IV intraoperatively | Laparoscopic resection of colon cancer. | Mean 72 (SD 6) | Pain | Quantitative synthesis. |
| Ma^78^ | 2021 | 260; 130//130 | Midazolam 30 μg/kg intrathecal intraoperatively | Dexmedetomidine 1 μg/kg intrathecal intraoperatively | Hip surgery | Mean 58 (SD 10) | Pain | Quantitative synthesis. |
| Mao^79^ | 2022 | 128; 64//64 | Remimazolam 0.2-0.3 mg/kg + 1-2 mg/kg/h (maintenance) IV intraoperatively | Propofol 2-3 mg/kg + 4-10 mg/kg/h (maintenance) IV intraoperatively | Urologic surgery | Median 51 (IQR 40-62) | QoR | Quantitative synthesis. |
| Maurice-Szamburski^80^ | 2015 | 971; 330//319/322 | Lorazepam 2.5 mg PO postoperatively | No medication; Placebo PO preoperatively | N/A | Mean 50 (SD 13) | Pain; Anxiety; Satisfaction; QoR | Quantitative synthesis. |
| McAteer^81^ | 1984 | 150; 50/50//50 | Midazolam 5-7.5 mg based on weight range IV preoperatively; Midazolam 5-7.5 mg based on weight range + atropine 0.6 mg IV preoperatively | 1-2 mL of mixture of papaveretum 20 mg + hyoscine 0.4 mg IV preoperatively | Gynaecological surgery | Mean 35 (SD 8) | Anxiety; Satisfaction | Qualitative synthesis.  Anxiety data presented as ordinal. Satisfaction data presented as dichotomous. |
| Meena^82^ | 2021 | 60; 30//30 | Midazolam 0.05 mg/kg + 0.1-0.2 mg/kg/h (maintenance) IV intraoperatively | Dexmedetomidine 1 μg/kg + 0.2-0.7 ug/kg/h (maintenance) IV intraoperatively | Oral cancer surgery | Mean 43 (SD 10) | Satisfaction | Quantitative synthesis. |
| Modir^83^ | 2022 | 105; 35//35/35 | Midazolam 2 mg intrathecal intraoperatively | Fentanyl 50 μg intrathecal preoperatively; Magnesium sulfate 100 mg intrathecal intraoperatively | Knee arthroplasty under spinal | Mean 55 (SD 5) | Pain | Quantitative synthesis. |
| Mohammed^84^ | 2023 | 60; 30//30 | Bupivacaine (0.5%) 20 mL + lignocaine (2%) 10 mL + with adrenaline (1:2,00,000)  midazolam 50 μg/kg intraoperative block | Bupivacaine (0.5%) 20 mL + Lignocaine (2%) 10 mL with adrenaline  (1:2,00,000) intraoperative block | Upper limb surgeries | Not reported | Pain | Quantitative synthesis. |
| Mokhtar^85^ | 2016 | 75; 37//38 | Midazolam 0.035 mg/kg IV preoperatively | Normal saline 2 mL IV preoperatively | Elective c-section | Mean 26 (SD 6) | Satisfaction | Quantitative synthesis. |
| Moon^86^ | 2018 | 37; 19//18 | Midazolam 1-2 mg bolus, 1-2 maintenance 30 min IV intraoperatively | 30-min VR program ( underwater view of the ocean while listening to narrations designed to induce relaxation and meditation) intraoperatively | Urologic surgery | Mean 69 (IQR 64-71) | Satisfaction | Qualitative synthesis. Ordinal data presented. |
| Moslemi^87^ | 2023 | 70; 35//35 | Midazolam 0.03 mg/kg IV intraoperatively | Placebo 0.03 mg/kg + dexmedetomidine 1 µg/kg in 20 mL normal saline intraoperatively | C-section | Mean 30 (SD 5) | Pain | Quantitative synthesis. |
| Moutzouros^88^ | 2021 | 62; 34//28 | Diazepam 5 mg + ketorolac 10 mg + gabapentin 300 mg + acetaminophen 1000 mg PO postoperatively | Hydrocodone 5 mg + acetaminophen 325 mg PO postoperatively | Arthroscopic ACL surgery | Mean 27 (SD 13) | Pain | Quantitative synthesis. |
| Naguib^89^ | 1999 | 75; 25//25/25 | Midazolam 15 mg sublingual preoperatively | Melatonin 5 mg sublingual preoperatively; Saline placebo sublingual preoperatively | Gynaecological laparoscopic procedures | Mean 30 (Range 19–44) | Anxiety | Quantitative synthesis. |
| Naguib^90^ | 2000 | 84; 12/12/12//12/12/12/12 | Midazolam 0.05 mg/kg sublingual preoperatively; Midazolam 0.1 mg/kg sublingual preoperatively; Midazolam 0.2 mg/kg sublingual preoperatively | Melatonin 0.05 mg/kg sublingual preoperatively; Melatonin 0.1 mg/kg sublingual preoperatively; Melatonin 0.2 mg/kg sublingual preoperatively; Saline sublingual preoperatively | Gynecological laparoscopic procedures | Mean 30 (SD 6) | Anxiety | Qualitative synthesis. No standard deviation reported. |
| Nanjegowda^91^ | 2011 | 50; 25//25 | Midazolam 2 mg + 0.5% hyperbaric bupivacaine intrathecal intraoperatively | Saline + 0.5% hyperbaric bupivacaine intrathecal intraoperatively | Knee arthroscopy | Mean 34 (SD 11) | Pain | Quantitative synthesis. |
| Nayak^92^ | 2023 | 60; 30//30 | Midazolam 2.5 mg intrathecal intraoperatively | Fentanyl 25 μg intrathecal intraoperatively | Total abdominal hysterectomy | Mean 44 (SD 7) | Pain | Quantitative synthesis. |
| Park^93^ | 2015 | 48; 25//23 | Diazepam 4 mg PO 1 night preoperatively | Pregabalin 300 mg PO 1 night preoperatively | Tonsillectomy | Mean 33 (SD 2) | Pain; Satisfaction | Quantitative synthesis. |
| Parikh^94^ | 2013 | 90; 45//45 | Midazolam 0.06 mg/kg + fentanyl 1 μg/kg intraoperatively | Dexmedetomidine 1 ug/kg IV bolus + 0.2 ug/kg/h infusion intraoperatively | Tympanoplasty | Mean 29 (SD 11) | Satisfaction | Quantitative synthesis. |
| Prasad^95^ | 2023 | 225; 75//75/75 | Midazolam 1-2 mg IV intraoperatively | Binaural tone (Frequency 3.5 Hz) music intraoperatively; Own choice of music intraoperatively | Surgery under spinal anesthesia | Mean 36 (SD 12) | Anxiety | Quantitative synthesis. |
| Przesmycki^96^ | 2011 | 80; 21//17/20/22 | Midazolam 7.5 mg PO preoperatively | Pregabalin 75 mg PO preoperatively; Pregabalin 150 mg PO preoperatively; Pregabalin 300 mg PO preoperatively | Abdominal hysterectomy | Mean 51 (SD 1) | Pain | Quantitative synthesis. |
| Pyeon^97^ | 2017 | 76; 25/27//24 | Triazolam 0.25 mg PO preoperatively; Triazolam 0.375 mg PO preoperatively | No medication preoperatively | Breast, thyroid, and abdominal surgery | Mean 45 (SD 7) | Satisfaction | Qualitative synthesis. Ordinal data presented. |
| Rao^98^ | 2024 | 56; 28//28 | Midazolam (dose not specified) intraoperatively | Buprenorphine (dose not specified) intraoperatively | Abdominal surgery | Mean 35 (SD 9) | Pain | Quantitative synthesis. |
| Roberts^99^ | 1976 | 44; 17//27 | Diazepam 7.5 mg IM preoperatively | Morphine sulfate 10 mg + scopolamine 0.4 mg IM preoperatively | Not reported | Not reported | Satisfaction | Qualitative synthesis. Ordinal data presented. |
| Russel^100^ | 1983 | 42; 22//20 | Lorazepam 2.5-5 mg PO preoperatively with dose determined by weight category. | Placebo PO preoperatively | TURP | Mean 70 (SD 8) | Anxiety | Qualitative synthesis. Ordinal data presented. |
| Ryu^101^ | 2024 | 34; 17//17 | Remimazolam 12 mg/kg/h until achieving loss of consciousness + bolus of remifentanil 1 μg/kg intraoperatively | Sevoflurane with propofol  1.5- 2 mg/kg IV  intraoperatively | TURBT | Mean 69 (SD 11) | QoR | Quantitative synthesis. |
| Senses^102^ | 2013 | 80; 40//40 | Midazolam 0.05 mg/kg IV bolus + 0.05mg/kg/h infusion intraoperatively | Dexmedetomidine 0.5 μg/kg IV bolus + 0.5 μg/kg/h infusion intraoperatively | Not reported | Mean 39 (SD 14) | Anxiety | Quantitative synthesis. |
| Seow^103^ | 1985 | 21; 11//10 | Diazepam loading dose of 20 ( ± 15) mg at a rate of 1 mg/min intraoperatively | 0.8% Chlormethiazole 10 mL/min infusion over 16 (±6) min intraoperatively | Lower limb and lower abdominal surgery | Mean 31 (SD 7) | Satisfaction | Qualitative synthesis. Dichotomous data presented. |
| Sharan^104^ | 2016 | 60; 30//30 | Midazolam 0.05 mg/kg followed by 2 mg increments until an adequate level of sedation was reached intraoperatively | Propofol 1–2 mg/kg to a maximum of 2 mg/kg followed by 20 mg increments if needed intraoperatively | Elective surgery | Mean 38 (SD 10) | Satisfaction | Qualitative synthesis. Ordinal data presented. |
| Siddiqui^105^ | 2022 | 66; 34//32 | Midazolam 7.5 mg PO 1 night preoperatively | Control: no pain reliever | Laparoscopic cholecystectomy | Mean 42 (SD 10) | Pain | Quantitative synthesis. |
| Silva-Jr^106^ | 2019 | 120; 53//67 | Midazolam 0.02 mg/kg bolus dose + 0.5 fentanyl µg/kg bolus dose intraoperatively | Dexmedetomidine 1ug/kg bolus dose + 0.2-0.8ug/kg/h infusion intraoperatively | Mixed non-cardiac surgery | Mean 75 (SD 5) | Pain | Qualitative synthesis. Ordinal data presented. |
| Singhal^107^ | 2024 | 100; 50//50 | Midazolam 0.03–0.05 mg/kg intermittent boluses intraoperatively | Dexmedetomidine a loading dose of 1.0 µg/kg for 10 min + 0.3–0.6 µg/kg/h IV intraoperatively | Percutaneous transluminal angioplasty | Mean 64 (SD 12) | Pain, Satisfaction | Quantitative synthesis. |
| Sirmela^108^ | 2023 | 75; 25//25/25 | Midazolam 0.05 mg/kg IV preoperatively | Dexmedetomidine 0.5 mcg/kg IV preoperatively; Normal saline IV preoperatively | Elective procedures under  spinal/intrathecal anaesthesia | Mean 55 (SD 3) | Pain | Quantitative synthesis. |
| Song^109^ | 2022 | 165; 82//83 | Remimazolam 1-2 mg/kg/h IV infusion intraoperatively | Desflurane inhalation 0.7-0.9 MAC intraoperatively | Laparoscopic cholecystectomy, robotic gynecologic surgery | Mean 43 (SD 10) | QoR | Quantitative synthesis. |
| Song^110^ | 2022 | 134; 65//69 | Midazolam 0.05 mg/kg IM preoperatively | Normal saline placebo 0.01 mg/kg IM preoperatively | Mixed non-cardiac surgery | Mena 47 (SD 11) | Pain, Satisfaction | Qualitative synthesis. Data reported narratively. |
| Sun^111^ | 2024 | 184; 46/46/46//46 | Remimazolam 0.1 mg/kg IV preoperatively; Estazolam 1 mg PO preoperatively; Remimazolam 0.1 mg/kg + estazolam 1 mg PO preoperatively | Normal saline 0.1 mL/kg preoperatively | Laparoscopic gastrointestinal surgery | Mean 49 (SD1 10) | Pain | Quantitative synthesis. |
| van Beek^112^ | 2020 | 192; 96//96 | Midazolam 3 mg IV preoperatively | Normal saline placebo IV preoperatively | Laparotomy | Mean 57 (SD 14) | Anxiety; QoR | Quantitative synthesis. |
| van Wijhe^113^ | 1985 | 203; 67//69/67 | Midazolam 70 μg/kg IM preoperatively | Normal saline IM preoperatively; Fentanyl 1.4 μg/kg + droperidol 70 μg/kg IM preoperatively | Orthopedic surgery | Mean 42 (SD 16) | Anxiety | Qualitative synthesis. Results reported narratively. |
| Wallace^114^ | 1984 | 90; 30//30/29 | Lorazepam 0.05 mg/kg (maximum 4 mg) IM preoperatively | Hydroxyzine 1.5 mg/kg (maximum, 100 mg) IM preoperatively; 1 ml of normal saline IM preoperatively | Not reported | Mean 29 (range 18-64) | Anxiety | Qualitative synthesis. Results reported narratively. |
| Wang^115^ | 2023 | 177; 88//89 | Midazolam 50 μg/kg/h IV intraoperatively | Dexmedetomidine 1.5 µg/kg/h IV intraoperatively | Below knee orthopedic surgery | Mean 56 (SD 14) | Pain | Quantitative synthesis. |
| Wender^116^ | 1977 | 140; 35/35//35/35 | Diazepam 7.5 mg IV preoperatively; Diazepam 15 mg IV preoperatively | Hydroxyzine 75 mg IV preoperatively; Hydroxyzine 150 mg IV preoperatively | Not reported | Mean 39 (SD not reported) | Satisfaction, Anxiety | Qualitative synthesis. Ordinal data presented. |
| Xu^117^ | 2020 | 83; 40//43 | Midazolam 0.05 mg/kg then 0.02 mg/kg/h IV infusion intraoperatively | Dexmedetomidine 0.5 μg/kg IV bolus + 0.3 μg/kg/h infusion intraoperatively | Partial and total laryngectomy | Not reported | Pain | Quantitative synthesis. |
| Yamakage^118^ | 2002 | 48; 12//12/12/12 | Triazolam 0.25 mg PO preoperatively | Control- no premedication; Zopiclone 7.5 mg PO preoperatively; Clonidine 0.15 mg PO preoperatively; | Not reported | Mean 45 (SD not reported) | Satisfaction | Qualitative synthesis. Dichotomous data reported. |
| Yegin^119^ | 2004 | 44; 22//22 | 0.5% hyperbaric bupivacaine 2 mL + 1 mL of 2 mg midazolam intrathecally intraoperatively | 0.5% hyperbaric bupivacaine 2 mL + saline 0.9% 1 mL intrathecally intraoperatively | Perianal surgery | Mean 36 (SD 15) | Pain | Quantitative synthesis. |
| Yoo^120^ | 2024 | 40; 20//20 | Remimazolam 6 mg/kg/h + remifentanil 0.1–0.2 μg/kg/min (induction), remimazolam 1–2 mg/kg/h + remifentanil 0.1–0.2 μg/kg/min (maintenance) IV intraoperatively | Sevoflurane 5% + remifentanil 0.1–  6 0.2 μg/kg/min  (induction), sevoflurane 1.6–2% + remifentanil 0.1–0.2 μg/kg/min (maintenance) IV intraoperatively | Elective laparoscopic cholecystectomy or hemicolectomy | Mean 53 (SD 11) | QoR, Pain | Quantitative synthesis. |
| Yue^121^ | 2023 | 200; 100//100 | Remimazolam 0.3 mg/kg + esketamine 0.3 mg/kg IV intraoperatively | Propofol 2 mg/kg + esketamine 0.3 mg/kg IV intraoperatively | Hysteroscopy | Median 30 | Pain | Quantitative synthesis. |
| Zakeri^122^ | 2017 | 93; 31//31/31 | Bupivacaine 10 mg + midazolam 2 mg intrathecal intraoperatively | Bupivacaine 10 mg + 1 ml normal saline intrathecal intraoperatively; bupivacaine 10 mg + magnesium sulfate 100 mg intrathecal intraoperatively | C-section | Mean 28 (SD 5) | Pain | Quantitative synthesis. |
| Zhou^123^ | 2024 | 150; 30/30/30/30//30 | Remimazolam 0.05  mg/kg IV intraoperatively; Remimazolam 0.1  mg/kg IV intraoperatively; Remimazolam 0.15  mg/kg IV intraoperatively; Remimazolam 0.2  mg/kg IV intraoperatively; all followed by sufentanil 0.15 μg/kg and propofol infusion | Control (sufentanil 0.15 μg/kg and propofol infusion) IV intraoperatively | Hysteroscopy | Mean 34 (SD 7) | Satisfaction, Pain | Quantitative synthesis. |

SD: standard deviation; IM: intramuscular; D&C: dilation and curettage; IV: intravenously; PO: orally; TURP: transurethral resection of the prostate; MAC: minimum alveolar concentration; TCI: target-controlled infusion; ACDF: anterior cervical discectomy and fusion; TURBT: transurethral resection of bladder tumor

**References:**

1. Aantaa R, Jaakola ML, Kallio A, Kanto J, Scheinin M, Vuorinen J. A comparison of dexmedetomidine, and alpha 2-adrenoceptor agonist, and midazolam as i.m. premedication for minor gynaecological surgery. *Br J Anaesth*. 1991;67(4):402-409.

2. Abdelemam RM, Fares KM, Kamal SM. Effect of combined epidural morphine and midazolam on postoperative pain in patients undergoing major abdominal cancer surgery. *Clin J Pain*. 2022;38(11):693-699.

3. Abdellatif AA, Elkabarity RH, Hamdy TAE. Dexmedetomedine vs midazolam sedation in middle ear surgery under local anesthesia: Effect on surgical field and patient satisfaction. *Egypt J Anaesth*. 2012;28(2):117-123.

4. Abdelrady MM, Fathy GM, Abdallah MAM, Ali WN. Comparison of the effect of adding midazolam versus fentanyl to intrathecal levobupivacaine in patients undergoing cesarean section: double-blind, randomized clinical trial. *Braz J Anesthesiol*. 2024;74(1):744385.

5. Majumdar S Dr, Gangadhar SB Dr, Ramesh CN Dr, Abhishek MS Dr. A comparative study of the effects of intrathecal midazolam(1mg) and fentanyl(25 micrograms) as additives to intrathecal hyperbaric bupivacaine 0.5%(15mg) in spinalanaesthesia. *ijsr*. Published online December 1, 2022:70-73.

6. Aldrich ER, Tam TY, Saylor LM, Crisp CC, Yeung J, Pauls RN. Intrarectal diazepam following pelvic reconstructive surgery: a double-blind, randomized placebo-controlled trial. *Am J Obstet Gynecol*. 2022;227(2):302.e1-302.e9.

7. Aleniewski MI, Bulas BJ, Maderazo L, Mendoza C, Stoelting RK, Tornetta FJ PhD. Intramuscular Lo razepa m Versus Pen tobarbi tal Premedica tion. *Anesth Analg*. 1977;56(4):489???492.

8. Amin SR, Sakr TE, Amin SE. Impact of timing of midazolam administration on incidence of postoperative nausea and vomiting in patients undergoing laparoscopic gynecological surgery: A randomized, double-blinded, controlled study. *Egypt J Anaesth*. 2022;38(1):580-586.

9. Amin OAI, Ibrahem MAM, Salem DAE. Nalbuphine versus midazolam as an adjuvant to intrathecal bupivacaine for postoperative analgesia in patients undergoing cesarean section. *J Pain Res*. 2020;13:1369-1376.

10. Ammar A, Mahmoud K, Kasemy Z. Effect of adding midazolam to bupivacaine during rectus sheath block: a randomised controlled trial. *Acta Anaesthesiol Scand*. 2018;62(6):857-862.

11. Anand LK, Sandhu M, Singh J, Mitra S. Evaluation of analgesic efficacy of pregabalin for postoperative pain relief after laparoscopic cholecystectomy: a double blind study. *Anaesth pain intensive care*. 2017;21(2):174-180.

12. Arifin J, Mochamat M, Pramadika T, Paramita D, Nurcahyo WI. Effects of immersive virtual reality on patient anxiety during surgery under regional anesthesia: A randomized clinical trial. *Anesth Pain Med*. 2023;13(2):e130790.

13. Ashok DS, Dharma VN, Satyanarayan ZA, Mamta B. Effect of intravenous dexmedetomidine or midazolam in patients undergoing upper limb surgeries under supraclavicular block. *Int J Pharm Clin Res*. 16(4):780-787.

14. Bagchi D, Mandal MC, Basu SR. Arousal time from sedation during spinal anaesthesia for elective infraumbilical surgeries: Comparison between propofol and midazolam. *Indian J Anaesth*. 2014;58(4):403-409.

15. Basuni AS. Addition of low-dose ketamine to midazolam and low-dose bupivacaine improves hemodynamics and postoperative analgesia during spinal anesthesia for cesarean section. *J Anaesthesiol Clin Pharmacol*. 2016;32(1):44-48.

16. Anand LK, Bindra T, Gombar KK, Goel P. Effect of pre-operative anxiolysis on postoperative pain in patients undergoing total abdominal hysterectomy under general anaesthesia: A randomized double-blind placebo controlled study. *J Anaesthesiol Clin Pharmacol*. 2010;26(2):172.

17. Bishnoi V, Kumar B, Bhagat H, Salunke P, Bishnoi S. Comparison of dexmedetomidine versus midazolam-fentanyl combination for monitored anesthesia care during burr-hole surgery for chronic subdural hematoma. *J Neurosurg Anesthesiol*. 2016;28(2):141-146.

18. Bond AC, Thompson MA. Droperidol/fentanyl, diazepam/pentazocine: a comparison. *Anaesthesia*. 1976;31(5):686-690.

19. Borracci T, Cappellini I, Campiglia L, et al. Preoperative medication with oral morphine sulphate and postoperative pain. *Minerva Anestesiol*. 2013;79(5):525-533.

20. Caumo W, Hidalgo MPL, Schmidt AP, et al. Effect of pre-operative anxiolysis on postoperative pain response in patients undergoing total abdominal hysterectomy. *Anaesthesia*. 2002;57(8):740-746.

21. Evaluation of Pregabalin for Postoperative Analgesia in Infraumbilical Surgeries: A Prospective, Randomized Clinical Study. *Randomized Clinical Study*.

22. Chen Y, Cai Y, Yu G, Zhang X, Hu T, Xue R. Safety and effcacy of remimazolam tosilate for sedation during combined spinal-epidural anesthesia for orthopedic procedures: a randomized controlled trial. *BMC Anesthesiol*. 2024;24(1):75.

23. Choi EK, Park SJ, Park C, Lim JA. Comparison of palonosetron with combined palonosetron and midazolam for preventing postoperative nausea and vomiting after laparoscopic cholecystectomy. *Medicine (Baltimore)*. 2021;100(33):e26997.

24. Choi JY, Lee HS, Kim JY, et al. Comparison of remimazolam-based and propofol-based total intravenous anesthesia on postoperative quality of recovery: A randomized non-inferiority trial. *J Clin Anesth*. 2022;82(110955):110955.

25. Dash LN, Bhanjadeo D, Meher AK, Kumar MM. Effectiveness of preoperative anxiolytics in reducing patient anxiety levels: Insights from a tertiary care hospital in bhawanipatna, odisha. *SSR Institute of International Journal of Life Sciences*. 2024;10(4):5907-5912.

26. Dyck JB, Chung F. A comparison of propranolol and diazepam for preoperative anxiolysis. *Can J Anaesth*. 1991;38(6):704-709.

27. El-Baradey GF, Elshmaa NS. The efficacy of adding dexamethasone, midazolam, or epinephrine to 0.5% bupivacaine in supraclavicular brachial plexus block. *Saudi J Anaesth*. 2014;8(Suppl 1):S78-83.

28. El-Deeb A, Ali Y, Rashdy H. Evaluation of combination antiemetic prophylaxis in high risk emetogenic patients undergoing thyroid surgery: A randomized double-blind study. *Egypt J Anaesth*. 2011;27(4):203-206.

29. Ellingson A, Haram K, Sagen N. Ketamine and diazepam as anaesthesia for forceps delivery. A comparative study. *Acta Anaesthesiol Scand*. 1977;21(1):37-40.

30. Elvir Lazo OL, White PF, Tang J, et al. Propofol versus midazolam for premedication: a placebo‑controlled, randomized double‑blinded study. *Minerva Anestesiol*. 2016;82(11):1170-1179.

31. Ersoy A, Kara D, Ervatan Z, Çakırgöz M, Kıran Ö. Sedation in hypoalbuminemic geriatric patients under spinal anesthesia in hip surgery. Midazolam or Propofol? *Saudi Med J*. 2015;36(10):1191-1198.

32. Feng W, Li Y, Li X, Li L. Levobupivacaine infiltration with midazolam sedation vs. Propofol-based general anesthesia for minimal invasive parathyroidectomy. *Int J Pharmacol*. 2018;15(1):84-91.

33. Fragen RJ, Caldwell N. Lorazepam premedication. *Anesth Analg*. 1976;55(6):792???796.

34. Franssen C, Hans P, Brichant JF, Noirot D, Lamy M. Comparison between alprazolam and hydroxyzine for oral premedication. *Can J Anaesth*. 1993;40(1):13-17.

35. Fredman B, Lahav M, Zohar E, Golod M, Paruta I, Jedeikin R. The effect of midazolam premedication on mental and psychomotor recovery in geriatric patients undergoing brief surgical procedures. *Anesth Analg*. 1999;89(5):1161-1166.

36. Gale G, Galloon S. Lorazepam as a premedication. *Can Anaesth Soc J*. 1976;23(1):22-29.

37. Ganguly S, Chattopadhyay S, Ranjan Basu S. Arousal time from intraoperative sedation in adult patients during spinal anaesthesia for infraumbilical surgeries- a comparison between dexmedetomidine and midazolam. *J Evol Med Dent Sci*. 2017;6(01):46-51.

38. Gao Y, Yan F. Comparison of intra and post-operative sedation efficacy of dexmedetomidinemidazolam and dexmedetomidine-propofol for major abdominal surgery. *Curr Drug Metab*. 2022;23(1):45-56.

39. Gilliland HE, Prasad BK, Mirakhur RK, Fee JP. An investigation of the potential morphine sparing effect of midazolam. *Anaesthesia*. 1996;51(9):808-811.

40. Giordano F, Giglio M, Sorrentino I, et al. Effect of preoperative music therapy versus intravenous midazolam on anxiety, sedation and stress in stomatology surgery: A randomized controlled study. *J Clin Med*. 2023;12(9). doi:10.3390/jcm12093215

41. Ha JH, Kwak KH, Seo JW, Lee SH, Park SS, Baek WE. Effectiveness of ondansetron and midazolam in the prevention of PONV after thyroidectomy. *Daehan Macwi’gwa Haghoeji*. 2007;53(3):344.

42. Haram K, Lund T, Sagen N, Boe OE. Comparison of thiopentone and diazepam as induction agents of anaesthesia for Caesarean section. *Acta Anaesthesiol Scand*. 1981;25(6):470-476.

43. Hashemian M, Zandrahimi F, Mirafzal A, Pakmanesh H, Amirkhosravi L. Post-traumatic stress and awareness in patient with traumatic lower limb surgery by neuraxial anesthesia: A randomized clinical trial. *Arch Psyc Psychother*. 2023;25(2):30-36.

44. He J, Zhang X, Li C, Fu B, Huang Y, Li H. Dexmedetomidine nasal administration improves perioperative sleep quality and neurocognitive deficits in elderly patients undergoing general anesthesia. *BMC Anesthesiol*. 2024;24(1):42.

45. Honarmand A, Safavi M, Khalili G, Mohammadnejad F. Prophylactic administration of haloperidol plus midazolam reduces postoperative nausea and vomiting better than using each drug alone in patients undergoing middle ear surgery. *Saudi J Anaesth*. 2012;6(2):145-151.

46. Hong JM, Han YH, Lee D, et al. Comparison of efficacy between palonosetron-midazolam combination and palonosetron alone for prevention of postoperative nausea and vomiting in patients undergoing breast surgery and patient controlled analgesia: A prospective, randomized, double-blind study: A CONSORT-compliant study. *Medicine (Baltimore)*. 2021;100(26):e26438.

47. Hong SW, Park JY, Rhee KY, Kim SH. Comparison emergence of sedation, using dexmedetomidine and remimazolam, in spinal anaesthesia - double blinded randomized controlled trial. *Int J Med Sci*. 2024;21(8):1552-1558.

48. Hu B, Zhou H, Zou X, Shi J, Li X, Tan L. A comparison of dexmedetomidine and midazolam for the prevention of postoperative nausea and vomiting caused by hemabate in cesarean delivery: A randomized controlled trial. *Drug Des Devel Ther*. 2020;14:2127-2133.

49. Ibrahim AE, Ghoneim MM, Kharasch ED, et al. Speed of recovery and side-effect profile of sevoflurane sedation compared with midazolam. *Anesthesiology*. 2001;94(1):87-94.

50. Ionescu D, Bãdescu C, Ilie A, et al. Melatonin as premedication for laparoscopic cholecystectomy:a double-blind, placebo-controlled study. *South Afr J Anaesth Analg*. 2008;14(4):8-11.

51. Jabalameli M, Honarmand A, Safavi M, Chitsaz M. Treatment of postoperative nausea and vomiting after spinal anesthesia for cesarean delivery: A randomized, double-blinded comparison of midazolam, ondansetron, and a combination. *Adv Biomed Res*. 2012;1(1):2.

52. Kain ZN, Sevarino FB, Rinder C, et al. Preoperative anxiolysis and postoperative recovery in women undergoing abdominal hysterectomy. *Anesthesiology*. 2001;94(3):415-422.

53. Kang H, Lim T, Lee HJ, Kim TW, Kim W, Chang HW. Comparison of the effect of dexmedetomidine and midazolam under spinal anesthesia for cesarean delivery: a randomized controlled trial, single center study in South Korea. *Anesth Pain Med*. 2023;18(2):159-168.

54. Kapdi M, Desai S. Comparative study of intrathecal preservative-free midazolam versus nalbuphine as an adjuvant to intrathecal bupivacaine (0.5%) in patients undergoing elective lower-segment caesarean section. *Ain-Shams J Anaesthesiol*. 2021;13(1). doi:10.1186/s42077-021-00151-9

55. Karbasfrushan A, Farhadi K, Amini-Saman J, Bazargan-Hejazi S, Ahmadi A. Effect of intrathecal midazolam in the severity of pain in cesarean section: a randomized controlled trail. *Iran Red Crescent Med J*. 2012;14(5):276-282.

56. *A Comparative Study between Oral Melatonin and Oral Alprazolam as Premedication on Preoperative Anxiety, Sedation and Cognitive Functions in Patients Undergoing Surgeries Under General Anaesthesia*.

57. Kestin IG, Harvey PB, Nixon C. Psychomotor recovery after three methods of sedation during spinal anaesthesia. *Br J Anaesth*. 1990;64(6):675-681.

58. Kim MH, Lee YM. Intrathecal midazolam increases the analgesic effects of spinal blockade with bupivacaine in patients undergoing haemorrhoidectomy. *Br J Anaesth*. 2001;86(1):77-79.

59. Kim S, Seo J, Jeon Y. Antiemetic effects of midazolam added to fentanyl-ropivacaine patient-controlled epidural analgesia after subtotal gastrectomy: A prospective, randomized, double-blind, controlled trial. *Curr Ther Res Clin Exp*. 2010;71(5):298-308.

60. Kim YH, Yoon SZ, Lim HJ, Yoon SM. Prophylactic use of midazolam or propofol at the end of surgery may reduce the incidence of emergence agitation after sevoflurane anaesthesia. *Anaesth Intensive Care*. 2011;39(5):904-908.

61. Kim WJ, Kang H, Shin HY, et al. Ramosetron, midazolam, and combination of ramosetron and midazolam for prevention of postoperative nausea and vomiting: a prospective, randomized, double-blind study. *J Int Med Res*. 2013;41(4):1203-1213.

62. Kim MH, Kim MS, Lee JH, Seo JH, Lee JR. Can quality of recovery be enhanced by premedication with midazolam?: A prospective, randomized, double-blind study in females undergoing breast surgery. *Medicine (Baltimore)*. 2017;96(7):e6107.

63. Kim H, Kim Y, Bae J, Yoo S, Lim YJ, Kim JT. Comparison of remimazolam and dexmedetomidine for intraoperative sedation in patients undergoing lower extremity surgery under spinal anesthesia: a randomized clinical trial. *Reg Anesth Pain Med*. 2024;49(2):110-116.

64. Kotani T, Ida M, Naito Y, Kawaguchi M. Comparison of remimazolam-based and propofol-based total intravenous anesthesia on hemodynamics during anesthesia induction in patients undergoing transcatheter aortic valve replacement: a randomized controlled trial. *J Anesth*. 2024;38(3):330-338.

65. Kowark A, Keszei AP, Schneider G, et al. Preoperative midazolam and patient-centered outcomes of older patients: The I-PROMOTE randomized clinical trial. *JAMA Surg*. 2024;159(2):129-138.

66. Kulkarni AM, Gurav S, Shibupaulose L. Anaesthetic and Haemodynamic Effects of Dexmedetomidine vs Midazolam used as Premedication in Minor Obstetrics and Gynaecological ProceduresA Prospective Interventional Study. *J Clin Diagn Res*. Published online 2022. doi:10.7860/jcdr/2022/56374.16460

67. Kumar S, Hussain M, Arun N, Kumar A, Kumar M. The effect of midazolam and dexmedetomidine sedation on block characteristic following spinal bupivacaine: A randomized comparative study. *Anesth Essays Res*. 2020;14(3):497-503.

68. *Small Dose of Midazolam Added to Fentanyl-Ropivacaine for Patient Controlled Epidural Analgesia after Subtotal Gastrectomy*.

69. *Comparison of Handholding and Conversation Alone or with Midazolam Premedication on Preoperative Anxiety in Adult Patients Undergoing Lumbar Spine Surgeries: A Three Arm Parallel Randomized Controlled Trial*.

70. Lee Y, Wang JJ, Yang YL, Chen A, Lai HY. Midazolam vs ondansetron for preventing postoperative nausea and vomiting: a randomised controlled trial. *Anaesthesia*. 2007;62(1):18-22.

71. Lee HJ, Lee HB, Kim YJ, Cho HY, Kim WH, Seo JH. Comparison of the recovery profile of remimazolam with flumazenil and propofol anesthesia for open thyroidectomy. *BMC Anesthesiol*. 2023;23(1):147.

72. Lee C, Lim J, Hong H, Yu H, Lee H. Effect of remimazolam on pain perception and opioid-induced hyperalgesia in patients undergoing laparoscopic urologic surgery-A prospective, randomized, controlled study. *Medicina (Kaunas)*. 2024;60(1). doi:10.3390/medicina60010123

73. Lee J, Kim DH, Ju JW, et al. Comparison of recovery profiles between total intravenous anaesthesia with propofol or remimazolam reversed with flumazenil in patients undergoing breast surgery: A randomised controlled trial. *Eur J Anaesthesiol*. 2024;41(3):199-207.

74. Lee J, Han DW, Kim NY, et al. Comparison of remimazolam versus sevoflurane on the postoperative Quality of recovery in cervical spine surgery: A prospective randomized controlled double-blind trial. *Drug Des Devel Ther*. 2024;18:121-132.

75. Liang S, Li S, Zhong Z, et al. The effect of lidocaine-prilocaine cream combined with or without remimazolam on VAS and APAIS anxiety score in patient undergoing spinal anesthesia. *Drug Des Devel Ther*. 2024;18:3429-3441.

76. Liu H, Gao M, Zheng Y, Sun C, Lu Q, Shao D. Effects of dexmedetomidine at different dosages on perioperative haemodynamics and postoperative recovery quality in elderly patients undergoing hip replacement surgery under general anaesthesia: a randomized controlled trial. *Trials*. 2023;24(1):386.

77. Liu T, Zhao H, Zhao X, Qu M. Comparison of remimazolam and propofol on postoperative delirium in elderly patients undergoing radical resection of colon cancer: A single-center prospective randomized controlled study. *Med Sci Monit*. 2024;30:e943784.

78. Ma P, Zeng H. Effects of epidural anesthesia combined with dexmedetomidine on blood pressure, sedation, analgesia and serum β-endorphin levels in patients with hip fractures. *Am J Transl Res*. 2021;13(6):6457-6467.

79. Mao Y, Guo J, Yuan J, Zhao E, Yang J. Quality of recovery after general anesthesia with remimazolam in patients’ undergoing urologic surgery: A randomized controlled trial comparing remimazolam with propofol. *Drug Des Devel Ther*. 2022;16:1199-1209.

80. Maurice-Szamburski A, Auquier P, Viarre-Oreal V, et al. Effect of sedative premedication on patient experience after general anesthesia: a randomized clinical trial. *JAMA*. 2015;313(9):916-925.

81. McATEER EJ, Dixon J, Whitwam JG. Intramuscular midazolam. *Anaesthesia*. 1984;39(12):1177-1182.

82. Meena R, Joshi A, Sherbina KM, Roy PS. Comparison of dexmedetomidine vs midazolam for sedation during Awake Fiberoptic Intubation in oral cancer surgeries- A randomised clinical study. *J Clin Diagn Res*. Published online 2021. doi:10.7860/jcdr/2021/49206.15294

83. Modir H, Moshiri E, Azami M, Sayafi-Sharifi TS. Comparison of impact of adjuvant treatment of midazolam, fentanyl, and magnesium sulfate with intrathecal bupivacaine on block characteristics and postoperative analgesia in knee arthroplasty. *J Acute Dis*. 2022;11(3):94-100.

84. Mohammed NB, Amod KS, Devaraj IC, Faiziya T. Efficacy of supraclavicular brachial plexus block with midazolam, bupivacaine and lignocaine in upper limb surgeries: A randomized comparative clinical study. *J Cardiovasc Dis Res*. 2023;14(3):1129-1133.

85. Mokhtar AM, Elsakka AI, Ali HM. Premedication with midazolam prior to cesarean delivery in preeclamptic parturients: A randomized controlled trial. *Anesth Essays Res*. 2016;10(3):631-636.

86. Moon JY, Shin J, Chung J, Ji SH, Ro S, Kim WH. Virtual reality distraction during endoscopic urologic surgery under spinal anesthesia: A randomized controlled trial. *J Clin Med*. 2018;8(1):2.

87. Moslemi F, Abri R, Ghadamkheir E. Comparison of the sedative effects of intravenous midazolam with intravenous dexmedetomidine and duration of spinal anesthesia with bupivacaine during cesarean section: A randomized double-blinded trial. *Interv Pain Med Neuromod*. 2023;3(1). doi:10.5812/ipmn-141007

88. Moutzouros V, Jildeh TR, Tramer JS, et al. Can we eliminate opioids after anterior cruciate ligament reconstruction? A prospective, randomized controlled trial. *Am J Sports Med*. 2021;49(14):3794-3801.

89. Naguib M, Samarkandi AH. Premedication with melatonin: a double-blind, placebo-controlled comparison with midazolam. *Br J Anaesth*. 1999;82(6):875-880.

90. Naguib M, Samarkandi AH. The comparative dose-response effects of melatonin and midazolam for premedication of adult patients: a double-blinded, placebo-controlled study. *Anesth Analg*. 2000;91(2):473-479.

91. Nanjegowda N, Nataraj MS, Kavaraganahalli DM, Kini G. The effects of intrathecal midazolam on the duration of analgesia in patients undergoing knee arthroscopy. *South Afr J Anaesth Analg*. 2011;17(3):255-259.

92. Nayak A, Ninave S, Tayade S, Tayade H. Intrathecal adjuvant midazolam versus fentanyl with hyperbaric bupivacaine for post-operative analgesia in women undergoing total abdominal hysterectomy. *Cureus*. 2023;15(6):e40565.

93. Park SS, Kim DH, Nam IC, Lee IH, Hwang JW. The effectiveness of pregabalin for post-tonsillectomy pain control: a randomized controlled trial. *PLoS One*. 2015;10(2):e0117161.

94. Parikh DA, Kolli SN, Karnik HS, Lele SS, Tendolkar BA. A prospective randomized double-blind study comparing dexmedetomidine vs. combination of midazolam-fentanyl for tympanoplasty surgery under monitored anesthesia care. *J Anaesthesiol Clin Pharmacol*. 2013;29(2):173-178.

95. Prasad M, Sethi P, Kumari K, et al. Comparison of binaural tone music vs patient choice music vs midazolam on perioperative anxiety in patients posted for surgery under spinal anaesthesia: A randomized control trial. *Cureus*. 2023;15(2):e35091.

96. Przesmycki K, Wiater-Kozioł E, Kotarski J, et al. Effect of pre-emptive pregabalin on pain intensity and morphine requirement after hysterectomy. *Anestezjol Intens Ter*. 2011;43(1):14-17.

97. Pyeon T, Chung S, Kim I, Lee S, Jeong S. The effect of triazolam premedication on anxiety, sedation, and amnesia in general anesthesia. *Korean J Anesthesiol*. 2017;70(3):292-298.

98. *Comparison of Postoperative Pain and Side Effects Associated with the Buprenorphine Vs Midazolam among Patients Undergoing Abdominal Surgeries*.

99. Roberts PA. Amnesia in the oprating room: a controlled study with diazepam (Valium) and morphine-scopolamine. *AANA J*. 1976;44(2):180-183.

100. Russell WJ. Lorazepam as a premedicant for regional anaesthesia. *Anaesthesia*. 1983;38(11):1062-1065.

101. Ryu KH, Lee SH, Shim JG, et al. Comparative study on the impact of remimazolam and sevoflurane on quality of recovery after transurethral resection of bladder tumor: A randomized controlled noninferiority study. *Medicine (Baltimore)*. 2024;103(31):e38962.

102. Senses E, Apan A, Köse EA, Oz G, Rezaki H. The effects of midazolam and dexmedetomidine infusion on peri-operative anxiety in regional anesthesia. *Middle East J Anesthesiol*. 2013;22(1):35-40.

103. Seow LT, Mather LE, Cousins MJ. Comparison of the efficay of chlormethiazole and diazepam as i.V.Sedatives for supplementation of extradural anaesthesia. *Br J Anaesth*. 1985;57(8):747-752.

104. Sharan R, Mohan B, Kaur H, Bala A. Efficacy and safety of propofol versus midazolam in fiberoptic endotracheal intubation. *Anesth Essays Res*. 2016;10(3):437-445.

105. Siddiqui FB, Umer MF, Moazzam H, Sarosh, Soomro U, Shah SA. Randomized controlled clinical trial to examine the efficacy of oral midazolam in post operative pain reduction in patients undergoing laparoscopic cholecystectomy. *Pakistan Journal of Medical & Health Sciences*. 2022;16(10):430-432.

106. Silva-Jr JM, Katayama HT, Nogueira FAM, Moura TB, Alves TL, de Oliveira BW. Comparison of dexmedetomidine and benzodiazepine for intraoperative sedation in elderly patients: a randomized clinical trial. *Reg Anesth Pain Med*. 2019;44(3):319-324.

107. Singhal R, Mansuri Z, Aggarwal A. Comparison of Midazolam and Dexmedetomidine when Combined with Fentanyl for Percutaneous Transluminal Angioplasty in Patients with Peripheral Artery Disease. *J Cardiovasc Dis Res*. 2024;15(6):1739-1747.

108. Sirmela A, Arthi S, Raja Kantham G, Saravanan S. Comparative Study to Evaluate the Effect of Intravenous Dexmedetomidine and Intravenous Midazolam to Prolong Intrathecal Bupivacaine Anaesthesia. *Res J Med Sci*. 2023;17(12):55-60.

109. Song SW, Jang YN, Yoon MW, Jeon YG. Quality of recovery in patients administered remimazolam versus those administered an inhalant agent for the maintenance of general anesthesia: a randomized control trial. *BMC Anesthesiol*. 2022;22(1):226.

110. Song SW, Jin Y, Lim H, Lee J, Lee KH. Effect of intramuscular midazolam premedication on patient satisfaction in women undergoing general anaesthesia: a randomised control trial. *BMJ Open*. 2022;12(6):e059915.

111. Sun B, Sun X. The effects of remimazolam in combination with estazolam on postoperative hemodynamics and pain intensity in patients undergoing laparoscopic gastrointestinal surgery. *BMC Surg*. 2024;24(1):240.

112. van Beek S, Kroon J, Rijs K, Mijderwijk HJ, Klimek M, Stolker RJ. The effect of midazolam as premedication on the quality of postoperative recovery after laparotomy: a randomized clinical trial. *Can J Anaesth*. 2020;67(1):32-41.

113. van Wijhe M, de Voogt-Frenkel E, Stijnen T. Midazolam versus fentanyl/droperidol and placebo as intramuscular premedicant. *Acta Anaesthesiol Scand*. 1985;29(4):409-414.

114. Wallace G, Mindlin LJ. A controlled double-blind comparison of intramuscular lorazepam and hydroxyzine as surgical premedicants. *Anesth Analg*. 1984;63(6):571-576.

115. Wang X, Zhang S, Wang C, et al. Real-time evaluation of the independent analgesic efficacy of dexmedetomidine. *BMC Anesthesiol*. 2023;23(1):68.

116. Wender RH, Conner JT, Bellville JW, Schehl D, Dorey F, Katz RL. Comparison of i.V. Diazepam and hydroxyzine as surgical premedicants. *Br J Anaesth*. 1977;49(9):907-912.

117. Xu R, Zhu Y, Lu Y, Li W, Jia J. Dexmedetomidine versus midazolam on cough and recovery quality after partial and total laryngectomy - a randomized controlled trial. *BMC Anesthesiol*. 2020;20(1):249.

118. Yamakage M, Tsuchiya S, Ohtsuka N, Iwasaki S, Namiki A. Usefulness of oral hypnotic premedication for volatile induction of anesthesia in adults. *J Anesth*. 2002;16(3):194-197.

119. Yegin A, Sanli S, Dosemeci L, Kayacan N, Akbas M, Karsli B. The analgesic and sedative effects of intrathecal midazolam in perianal surgery. *Eur J Anaesthesiol*. 2004;21(8):658-662.

120. Yoo YM, Park JH, Lee KH, Yi AH, Kim TK. The incidences of nausea and vomiting after general anesthesia with remimazolam versus sevoflurane: a prospective randomized controlled trial. *Korean J Anesthesiol*. 2024;77(4):441-449.

121. Yue L, Ma X, Li N, et al. Remimazolam versus propofol in combination with esketamine for surgical abortion: A double-blind randomized controlled trial. *Clin Transl Sci*. 2023;16(9):1606-1616.

122. Zakeri H, Rajabi M, Badpa M, Abdollahi M, Salehi S, Hosseinipour A. Meperidine plus Bupivacaine as spinal anaesthesia in woman undergoing caesarean surgery: clinical trial study. *Biomed sci*. 2017;28(8):3783‐7.

123. Zhou YH, Li SX, Li L, et al. Effect of remimazolam supplementation on propofol requirements during hysteroscopy: A double-blind, dose-response study. *Anesth Analg*. 2024;139(6):1309-1316.
