# Supplementary material for: Effects of perioperative benzodiazepine administration on postoperative patient-reported outcomes: a systematic review and meta-analysis of randomised controlled trials
Source: Br J Anaesth. 2025 Sep 30;135(6):1741–52. doi: 10.1016/j.bja.2025.09.013 (PMC12799406; doi:10.1016/j.bja.2025.09.013)
Supplement: Multimedia component 4 [file mmc4.docx]

**Appendix 4: Summary overview of included studies**

| **Benzodiazepine Used** | |  |
| --- | --- | --- |
| Midazolam | Aanta (1991), Abdelemam (2022), Abdellatif (2012), Abdelrady (2024), Abhishek (2022), Amin (2019), Amin (2022), Ammar (2018), Arifin (2023), Ashok (2024), Bagchi (2014), Basuni (2016), Bishnoi (1998), Borracci (2013), Choi (2021), Dash (2024), El-Baradey (2014), El-Deeb (2011), Elvir Lazo (2016), Ersoy (2015), Feng (2019), Fredman (1999), Ganguly (2016), Gao (2022), Gilliland (1996), Giordano (2023), Ha (2007), Hashemian (2023), Honarmand (2012), Hong (2021), Hu (2020), Ibrahim (2001), Ionescu (2008), Jabalameli (2012), Karbasfrushan (2012), Kain (2001), Kang (2023), Kapdi (2021), Kestin (1990), Kim (2001), Kim (2010), Kim (2011), Kim (2013), Kim (2017), Kotani (2024), Kowark (2024), Kulkarni (2022), Kumar (2020), Kwak (2008), Lal (2023), Lee (2007), Liu (2023), Ma (2021), McAteer (1984), Meena (2021), Modir (2022), Mokhtar (2016), Moon (2018), Moslemi (2023), Naguib (1999), Naguib (2000), Nanjegowda (2011), Nayak (2023), Nizamuddeen (2023), Parikh (2013), Prasad (2023), Przesmycki (2011), Rao (2024), Senses (2013), Sharan (2016), Siddiqui (2022), Silva-Jr (2019), Singhal (2024), Sirmela (2023), Song (2022), van Beek (2020), van Wijhe (1985), Wang (2023), Xu (2020), Yegin (2004), Zakeri (2017) | 81 |
| Alprazolam | Anand (2017), Bindra (2010), Franssen (1993), He (2024), Keerthy (2022), | 5 |
| Triazolam | Pyeon (2017), Yamakage (2002), | 2 |
| Diazepam | Aldrich (2022), Bond (1976), Caumo (2002), Chandra (2024), Dyck (1991), Ellingson (1977), Haram (1981), Moutzouros (2021), Park (2015), Roberts (1976), Seow (1985), Wender (1977) | 12 |
| Lorazepam | Aleniewski (1977), Fragen (1976), Gale (1976), Maurice-Szamburski (2015), Russel (1983), Ryu (2024), Wallace (1984), | 7 |
| Remimazolam | Chen (2024), Choi (2022), Hong (2024), Kim (2024), Lee (2023), Lee, Cheol (2024), Lee, Jaemoon (2024), Lee, Jiwon (2024), Liang (2024), Liu (2024), Mao (2022), Song (2022), Yoo (2024), Yue (2023), Zhou (2024) | 15 |
| 2 or more agents used | Sun (2024) | 1 |
| **Outcome Assessed** | |  |
| Pain | Aanta (1991), Abdelemam (2022), Abdelrady (2024), Aldrich (2022), Amin (2022), Amin (2019), Ammar (2018), Anand (2017), Ashok (2024), Bindra (2010), Bond (1976), Borracci (2013), Caumo (2002), Chandra (2024), Choi (2021), Dash (2024), El-Baradey (2014), El-Deeb (2011), Feng (2019), Gao (2022), Gilliland (1996), Ha (2007), Honarmand (2012), Hong (2021), Hong (2024), Jabalameli (2012), Kain (2001), Kang (2023), Kapdi (2021), Karbasfrushan (2012), Kim (2001), Kim (2010), Kim (2013), Kumar (2020), Kwak (2008), Lee (2007), Lee, Cheol (2024), Lee, Jaemoon (2024), Lee, Jiwon (2024), Liu (2023), Liu (2024), Ma (2021), Maurice-Szamburski (2015), Modir (2022), Moslemi (2023), Moutzouros (2021), Nanjegowda (2011), Nayak (2023), Nizamuddeen (2023), Park (2015), Prasad (2023), Przesmycki (2011), Rao (2024), Siddiqui (2022), Silva-Jr (2019), Singhal (2024), Sirmela (2023), Song (2022), Sun (2024), Wang (2023), Xu (2020), Yegin (2004), Yoo (2024), Yue (2023), Zakeri (2017), Zhou (2024) | 66 |
| Anxiety | Arifin (2023), Dash (2024), Giordano (2023), Hashemian (2023), Lal (2023), Prasad (2023), Bindra (2010), Caumo (2002), Dyck (1991), Elvir Lazo (2016), Fredman (1999), Gale (1976), He (2024), Ionescu (2008), Kain (2001), Keerthy (2022), Kestin (1990), Kulkarni (2022), Liang (2024), Maurice-Szamburski (2015), McAteer (1984), Naguib (1999), Naguib (2000), Russel (1983), Senses (2013), van Beek (2020), van Wijhe (1985) | 27 |
| Satisfaction | Abdellatif (2012), Abdelrady (2024), Abhishek (2022), Aldrich (2022), Aleniewski (1977), Anand (2017), Arifin (2023), Bagchi (2014), Basuni (2016), Bindra (2010), Bishnoi (1998), Caumo (2002), Chen (2024), El-Baradey (2014), El-Deeb (2011), Ersoy (2015), Fragen (1976), Fredman (1999), Ganguly (2016), Giordano (2023), Hu (2020), Ibrahim (2001), Kain (2001), Kim (2010), Kim (2024), Kowark (2024), Maurice-Szamburski (2015), McAteer (1984), Meena (2021), Mokhtar (2016), Moon (2018), Park (2015), Parikh (2013), Pyeon (2017), Roberts (1976), Seow (1985), Sharan (2016), Singhal (2024), Song (2022), Wender (1977), Yamakage (2002), Zhou (2024) | 42 |
| Quality of Recovery | Choi (2022), Ellingson (1977), Haram (1981), Kim (2017), Kotani (2024), Lee (2023), Lee, Jaemoon (2024), Lee, Jiwon (2024), Mao (2022), Maurice-Szamburski (2015), Ryu (2024), Song (2022), van Beek (2020), Yoo (2024) | 14 |
| **Type of Surgery** | |  |
| Gynecologic surgery | Aanta (1991), Aldrich (2022), Amin (2022), Bindra (2010), Caumo (2002), Dyck (1991), Gale (1976), Gilliland (1996), Haram (1981), He (2024), Hu (2020), Jabalameli (2012), Kain (2001), Kang (2023), Kapdi (2021), McAteer (1984), Naguib (1999), Naguib (2000), Nayak (2023), Przesmycki (2011), Yue (2023), Zhou (2024) | 22 |
| Obstetric surgery | Abdelrady (2024), Amin (2019), Basuni (2016), Ellingson (1977), Karbasfrushan (2012), Mokhtar (2016), Moslemi (2023), Zakeri (2017) | 8 |
| General surgery | Abdelemam (2022), Ammar (2018), Anand (2017), Bagchi (2014), Borracci (2013), Chandra (2024), Choi (2021), Choi (2022), El-Deeb (2011), Feng (2019), Ganguly (2016), Gao (2022), Giordano (2023), Ha (2007), Hong (2021), Ionescu (2008), Kim (2013), Kim (2010), Kim (2017), Kim (2001), Kumar (2020), Kwak (2008), Lee (2023), Lee, Jaemoon (2024), Liu (2024), Pyeon (2017), Rao (2024), Ryu (2024), Siddiqui (2022), Sun (2024), Yegin (2004), | 32 |
| Orthopedic surgery | Ashok (2024), Bishnoi (1998),Chen (2024), El-Baradey (2014), Ellingson (2016), Ersoy (2015), Hashemian (2023), Hong (2024), Kim (2024), Lal (2023), Liu (2023), Lee, Jiwon (2024), Liang (2024), Ma (2021), Modir (2022), Moutzouros (2021), Nanjegowda (2011), Nizamuddeen (2023), Seow (1985), van Wijhe (1985), Wang (2023) | 21 |
| ENT surgery | Abdellatif (2012), Abhishek (2022), Honarmand (2012), Meena (2021), Park (2015), Parikh (2013), Xu (2020) | 7 |
| Urologic surgery | Fredman (1999), Kestin (1990), Lee, Cheol (2024), Mao (2022), Moon (2018), Russel (1983) | 6 |
| Cardiac surgery/Interventional cardiology | Kotani (2024), Singhal (2024) | 2 |
| Mixed non-cardiac surgeries | Arifin (2023), Bond (1976), Franssen (1993), Keerthy (2022), Kulkarni (2022), Lee (2007), Maurice-Szamburski (2015), Sharan (2016), Silva-Jr (2019), Song (2022), Song (2022), van Beek (2020) | 12 |
| Not reported | Aleniewski (1977), Dash (2024), Fragen (1976), Ibrahim (2001), Kim (2010), Kowark (2024), Prasad (2023), Roberts (1976), Senses (2013), Sirmela (2023), Wallace (1984), Wender (1977), Yamakage (2002) | 13 |
| **Comparators Used** | |  |
| Multiple | Abdelemam (2022), Abdelrady (2024), Amin (2019), Basuni (2016), Bond (1976), El-Baradey (2014), El-Deeb (2011), Gale (1976), Gao (2022), Hong (2021), Kapdi (2021), Kim (2010), Lee, Cheol (2024), Lee, Jaemoon (2024), Lee, Jiwon (2024), Liang (2024), Liu (2024), Kwak (2008), McAteer (1984), Moutzouros (2021), Nanjegowda (2011), Nizamuddeen (2023), Roberts (1976), Yue (2023), Yoo (2024), Zhou (2024) | 26 |
| None | Maurice-Szamburski (2015), Pyeon (2017), Siddiqui (2022), Yamakage (2002) | 4 |
| Placebo | Aldrich (2022), Ammar (2018), Bindra (2010), Caumo (2002), Dash (2024), Dyck (1991), Fragen (1976), Fredman (1999), Gilliland (1996), Ionescu (2008), Kain (2001), Kowark (2024), Maurice-Szamburski (2015), Russel (1983), van Beek (2020) | 15 |
| Saline | Aanta (1991), Amin (2022), Choi (2021), El-Deeb (2011), Elvir Lazo (2016), Ha (2007), He (2024), Honarmand (2012), Hu (2020), Kim (2017), Liu (2023), Mokhtar (2016), Naguib (1999), Naguib (2000), Sirmela (2023), Song (2022), Sun (2024), van Wijhe (1985), Wallace (1984) | 19 |
| Dexmedetomidine | Aanta (1991), Abdellatif (2012), Abhishek (2022), Ashok (2024), Bishnoi (1998), Chen (2024), Ganguly (2016), He (2024), Hong (2024), Hu (2020), Kang (2023), Kim (2024), Kulkarni (2022), Kumar (2020), Ma (2021), Meena (2021), Moslemi (2023), Parikh (2013), Senses (2013), Silva-Jr (2019), Singhal (2024), Sirmela (2023), Wang (2023), Xu (2020) | 24 |
| Propofol | Bagchi (2014), Choi (2022), Elvir Lazo (2016), Ersoy (2015), Feng (2019), Hashemian (2023), Kestin (1990), Kim (2011), Kotani (2024), Lee (2023), Mao (2022), Ryu (2024), Sharan (2016) | 13 |
| Ondansetron | Ha (2007), Jabalameli (2012), Lee (2007) | 3 |
| Morphine sulphate | Borracci (2013) | 1 |
| Thiopentone | Haram (1981) | 1 |
| Fentanyl | Modir (2022), Nayak (2023) | 2 |
| Hydroxyzine | Franssen (1993), Wallace (1984), Wender (1977) | 3 |
| Ketamine | Ellingson (1977) | 1 |
| Ramosetron | Kim (2013) | 1 |
| Sevofluorane | Ibrahim (2001) | 1 |
| Pregabalin | Anand (2017), Chandra (2024), Park (2015), Przesmycki (2011) | 4 |
| Desflurane | Song (2022) | 1 |
| Buprenorphine | Rao (2024) | 1 |
| Pentobarbital | Aleniewski (1977) | 1 |
| Virtual Reality | Arifin (2023), Moon (2018) | 2 |
| Propranolol | Dyck (1991) | 1 |
| Bupivacaine | Karbasfrushan (2012), Kim (2001), Yegin (2004), Zakeri (2017) | 4 |
| Music | Giordano (2023), Prasad (2023) | 2 |
| Haloperidol | Honarmand (2012) | 1 |
| Melatonin | Ionescu (2008), Keerthy (2022), Naguib (1999), Naguib (2000) | 4 |
| Hand-holding and conversation | Lal (2023) | 1 |
| Magnesium sulfate | Modir (2022) | 1 |
| Binaural tone | Prasad (2023) | 1 |
| Chlormethiazole | Seow (1985) | 1 |
| Zopiclone | Yamakage (2002) | 1 |
| Clonidine | Yamakage (2002) | 1 |
| **Time Interval Studied** | |  |
| </= 24 hours postoperatively | Aanta (1991), Abdelrady (2024), Aleniewski (1977), Amin (2022), Amin (2019), Anand (2017), Arifin (2023), Ashok (2024), Bagchi (2014), Basuni (2016), Bishnoi (1998), Caumo (2002), Chandra (2024), Chen (2024), Choi (2021), Dash (2024), Dyck (1991), Ellingson (1977), Elvir Lazo (2016), Ersoy (2015), Feng (2019), Fragen (1976), Franssen (1993), Fredman (1999), Gao (2022), Giordano (2023), Ha (2007), Honarmand (2012), Hong (2024), Hu (2020), Ionescu (2008), Jabalameli (2012), Karbasfrushan (2012), Kapdi (2021), Kestin (1990), Kim (2013), Kim (2001), Kim (2010), Kim (2024), Kulkarni (2022), Kumar (2020), Lal (2023), Lee (2007), Lee (2023), Lee, Jaemoon (2024), Liang (2024), Liu (2023), Ma (2021), Maurice-Szamburski (2015), McAteer (1984), Meena (2021), Modir (2022), Moon (2018), Moslemi (2023), Naguib (1999), Naguib (2000), Nanjegowda (2011), Nayak (2023), Nizamuddeen (2023), Prasad (2023), Przesmycki (2011), Rao (2024), Russel (1983), Seow (1985), Sharan (2016), Siddiqui (2022), Singhal (2024), Sirmela (2023), Song (2022), Xu (2020), Yegin (2004), Yoo (2024), Zakeri (2017), Zhou (2024) | 74 |
| >24 hours | Abhishek (2022), Bindra (2010), El-Baradey (2014), Gale (1976), Hashemian (2023), Keerthy (2022), Kim (2017), Kotani (2024), Mao (2022), Mokhtar (2016), Moutzouros (2021), Parikh (2013), Pyeon (2017), Roberts (1976), Ryu (2024), Silva-Jr (2019), Wallace (1984), Wender (1977), Yamakage (2002) | 19 |
| Both of above | Abdelemam (2022), Aldrich (2022), Ammar (2018), Bond (1976), Borracci (2013), Choi (2022), Gilliland (1996), He (2024), Hong (2021), Kain (2001), Kang (2023), Kim (2010), Kowark (2024), Kwak (2008), Lee, Cheol (2024), Lee, Jiwon (2024), Liu (2024), Park (2015), Senses (2013), Sun (2024), van Beek (2020), van Wijhe (1985), Wang (2023), Yue (2023) | 24 |
| At time of discharge | Abdellatif (2012), Ibrahim (2001) | 2 |
| Not reported | El-Deeb (2011), Ganguly (2016), Haram (1981), Song (2022) | 4 |
| **Used multiple benzodiazepine arms that were combined for analysis** | |  |
| Same benzodiazepine of different doses in separate arms | Fredman (1999), Kim (2001), Naguib (2000), Pyeon (2017), Wender (1977), Zhou (2024) | 6 |
| Same benzodiazepine mixed with different drugs/interventions in separate arms | Abdeleman (2022), Honarmand (2012), Jabalameli (2012), Kestin (1990), Kim (2013), Lal (2023), McAteer (1984), | 7 |
| Used 2 or more benzodiazepines | Fragen (1976), Sun (2024) | 2 |
| Same benzodiazepine of same dose at different time | Amin (2022), | 1 |
| **Time of Intervention** | |  |
| Preoperative | Aanta (1991), Aleniewski (1977), Anand (2017), Arifin (2023), Bindra (2010), Borracci (2013), Caumo (2002), Chandra (2024), Dash (2024), Dyck (1991), Elvir Lazo (2016), Fragen (1976), Franssen (1993), Fredman (1999), Gale (1976), Giordano (2023), He (2024), Ionescu (2008), Kain (2001), Keerthy (2022), Kim (2017), Kowark (2024), Kulkarni (2022), Kumar (2020), Lee, Jaemoon (2024),, Liang (2024), McAteer (1984), Mokhtar (2016), Naguib (1999), Naguib (2000), Park (2015), Przesmycki (2011), Pyeon (2017), Roberts (1976), Russel (1983), Siddiqui (2022), Sirmela (2023), Song (2022), Sun (2024), van Beek (2020), van Wijhe (1985), Wallace (1984), Wender (1977), Yamakage (2002) | 44 |
| Intraoperative | Abdelemam (2022), Abdellatif (2012), Abdelrady (2024), Abhishek (2022), Amin (2019), Ammar (2018), Ashok (2024), Bagchi (2014), Basuni (2016), Bishnoi (1998), Bond (1976), Chen (2024), Choi (2021), Choi (2022), El-Baradey (2014), El-Deeb (2011), Ellingson (1977), Ersoy (2015), Ganguly (2016), Gilliland (1996), Ha (2007), Haram (1981), Hashemian (2023), Honarmand (2012), Hong (2021), Hong (2024), Hu (2020), Ibrahim (2001), Kapdi (2021), Karbasfrushan (2012), Kestin (1990), Kim (2001), Kim (2011), Kim (2013), Kim (2024), Kotani (2024), Lal (2023), Lee (2007), Lee (2023), Lee, Cheol (2024), Lee, Jiwon (2024), Liu (2023), Liu (2024), Ma (2021), Mao (2022), Meena (2021), Modir (2022), Moon (2018), Moslemi (2023), Nanjegowda (2011), Nayak (2023), Nizamuddeen (2023), Parikh (2013), Prasad (2023), Rao (2024), Ryu (2024), Senses (2013), Seow (1985), Sharan (2016), Silva-Jr (2019), Singhal (2024), Song (2022), Wang (2023), Xu (2020), Yegin (2004), Yoo (2024), Yue (2023), Zakeri (2017), Zhou (2024) | 69 |
| Postoperative | Aldrich (2022), Jabalameli (2012), Kang (2023), Kim (2010), Kwak (2008), Maurice-Szamburski (2015), Moutzouros (2021) | 7 |
| Multiple time points of administration | Amin (2022), Feng (2019), Gao (2022) | 3 |
| **Route of Administration** | |  |
| IV | Abdellatif (2012), Abhishek (2022), Amin (2022), Arifin (2023), Ashok (2024), Bagchi (2014), Bishnoi (1998), Bond (1976), Chen (2024), Choi (2021), Choi (2022) Dash (2024), El-Deeb (2011), Ellingson (1977), Elvir Lazo (2016), Ersoy (2015), Feng (2019), Fredman (1999), Ganguly (2016), Gao (2022), Gilliland (1996), Giordano (2023), Ha (2007), Haram (1981), Hashemian (2023), Honarmand (2012), Hong (2021), Hong (2024), Hu (2020), Ibrahim (2001), Jabalameli (2012), Kang (2023), Kestin (1990), Kim (2013), Kim (2010), Kim (2011), Kim (2017), Kim (2024), Kotani (2024), Kulkarni (2022), Kumar (2020), Kwak (2008), Lal (2023), Lee (2007), Lee (2023), Lee, Cheol (2024), Lee, Jaemoon (2024), Lee, Jiwon (2024), Liang (2024), Liu (2023), Liu (2024), Mao (2022), McAteer (1984), Meena (2021), Mokhtar (2016), Moon (2018), Moslemi (2023), Parikh (2013), Prasad (2023), Ryu (2024), Senses (2013), Seow (1985), Sharan (2016), Silva-Jr (2019), Singhal (2024), Sirmela (2023), Song (2022), van Beek (2020), Wang (2023), Wender (1977), Xu (2020), Yoo (2024), Yue (2023), Zhou (2024) | 74 |
| Oral | Anand (2017), Bindra (2010), Borracci (2013), Caumo (2002), Chandra (2024), Dyck (1991), Franssen (1993), He (2024), Ionescu (2008), Kain (2001), Keerthy (2022), Kowark (2024), Maurice-Szamburski (2015), Moutzouros (2021), Park (2015), Przesmycki (2011), Pyeon (2017), Russel (1983), Siddiqui (2022), Sun (2024), Yamakage (2002) | 21 |
| Intrathecal | Abdelemam (2022), Abdelrady (2024), Amin (2019), Basuni (2016), Karbasfrushan (2012), Kapdi (2021), Kim (2001), Ma (2021), Modir (2022), Nanjegowda (2011), Nayak (2023), Yegin (2004), Zakeri (2017) | 13 |
| IM | Aanta (1991), Aleniewski (1977), Fragen (1976), Gale (1976), Roberts (1976), Song (2022), van Wijhe (1985), Wallace (1984) | 8 |
| Sublingual | Naguib (1999), Naguib (2000) | 2 |
| Intrarectal | Aldrich (2022) | 1 |
| Perineural | Ammar (2022), El-Baradey (2014), Nizamuddeen (2023) | 3 |
| Not reported | Rao (2024) | 1 |
| **Average Age of Study Population** | |  |
| Young (< 60) | Aanta (1991), Abdelemam (2022), Abdellatif (2012), Abdelrady (2024), Abhishek (2022), Aleniewski (1977), Amin (2022), Amin (2019), Ammar (2018), Anand (2017), Arifin (2023), Ashok (2024), Bagchi (2014), Basuni (2016), Bindra (2010), Bishnoi (1998), Bond (1976), Caumo (2002), Chandra (2024), Chen (2024), Choi (2021), Choi (2022), Dash (2024), Dyck (1991), El-Baradey (2014), El-Deeb (2011), Ellingson (1977), Elvir Lazo (2016), Fragen (1976), Franssen (1993), Gale (1976), Ganguly (2016), Gao (2022), Gilliland (1996), Ha (2007), Haram (1981), Hashemian (2023), Honarmand (2012), Hong (2021), Hong (2024), Hu (2020), Ibrahim (2001), Ionescu (2008), Jabalameli (2012), Karbasfrushan (2012), Kain (2001), Kang (2023), Kapdi (2021), Kim (2001), Kim (2010), Kim (2011), Kim (2013), Kim (2017), Kim (2024), Kulkarni (2022), Kumar (2020), Kwak (2008), Lal (2023), Lee (2007), Lee (2023), Lee, Cheol (2024), Lee, Jaemoon (2024), Lee, Jiwon (2024), Liang (2024), Ma (2021), Mao (2022), Maurice-Szamburski (2015), McAteer (1984), Meena (2021), Modir (2022), Mokhtar (2016), Moslemi (2023), Moutzouros (2021), Naguib (1999), Naguib (2000), Nanjegowda (2011), Nayak (2023), Park (2015), Parikh (2013), Prasad (2023), Przesmycki (2011), Pyeon (2017), Senses (2013), Seow (1985), Sharan (2016), Siddiqui (2022), Sirmela (2023), Song (2022), Song (2022), Sun (2024), van Beek (2020), van Wijhe (1985), Wallace (1984), Wang (2023), Wender (1977), Yamakage (2002), Yegin (2004), Yoo (2024), Yue (2023), Zhou (2024) | 100 |
| Elderly (> 60) | Aldrich (2022), Borracci (2013), Ersoy (2015), Feng (2019), Fredman (1999), Giordano (2023), He (2024), Kestin (1990), Kotani (2024), Kowark (2024), Liu (2023), Liu (2024), Moon (2018), Russel (1983), Ryu (2024), Silva-Jr (2019), Singhal (2024) | 17 |
| Not reported | Keerthy (2022), Nizamuddeen (2023), Rao (2024), Roberts (1976), Xu (2020), Zakeri (2017) | 6 |
| **Risk of Bias** | |  |
| High | Arifin (2023), Ashok (2024), Bagchi (2014), Bond (1976), Chen (2024), Dyck (1991), El-Baradey (2014), Ellingson (1977), Feng (2019), Franssen (1993), Giordano (2023), Haram (1981), He (2024), Hong (2021), Kain (2001), Kapdi (2021), Keerthy (2022), Kotani (2024), Kulkarni (2022), Lal (2023), Lee, Cheol (2024), Ma (2021), Moon (2018), Moslemi (2023), Moutzouros (2021), Naguib (1999), Naguib (2000), Nizamuddeen (2023), Prasad (2023), Pyeon (2017), Rao (2024), Roberts (1976), Seow (1985), Sharan (2016), Sun (2024), Yamakage (2002), Zakeri (2017) | 37 |
| Low | Aanta (1991), Abdelemam (2022), Abdellatif (2012), Abdelrady (2024), Abhishek (2022), Aldrich (2022), Aleniewski (1977), Amin (2019), Amin (2022), Ammar (2018), Anand (2017), Basuni (2016), Bindra (2010), Bishnoi (1998), Borracci (2013), Caumo (2002), Chandra (2024), Choi (2021), Choi (2022), Dash (2024), El-Deeb (2011), Elvir-Lazo (2016), Ersoy (2015), Fragen (1976), Fredman (1999), Gale (1976), Ganguly (2016), Gao (2022), Gilliland (1996), Ha (2007), Hashemian (2023), Honarmand (2012), Hong (2024), Hu (2020), Ibrahim (2001), Ionescu (2008), Jabalameli (2012), Kang (2023), Karbasfrushan (2012), Kestin (1990), Kim (2010), Kim (2001), Kim (2011), Kim (2017), Kim (2013), Kim (2024), Kowark (2024), Kumar (2020), Kwak (2008), Lee, Jaemoon (2024), Lee, Jiwon (2024), Lee (2023), Lee (2007), Liang (2024), Liu (2023), Liu (2024), Mao (2022), Maurice-Szamburski (2020), McAteer (1984), Meena (2021), Modir (2022), Mohktar (2016), Nanjegowda (2011), Nayak (2023), Parikh (2013), Park (2015), Przesmycki (2011), Russell (1983), Ryu (2024), Senses (2013), Siddiqui (2022), Silva-Jr (2019), Singhal (2024), Sirmela (2023), Song (2022), Song (2022), van Beek (2020), van Wijhe (1985), Wallace (1984), Wang (2023), Wender (1977), Xu (2020), Yegin (2004), Yoo (2024), Yue (2023), Zhou (2024) | 86 |
