# Supplementary material for: Effects of perioperative benzodiazepine administration on postoperative patient-reported outcomes: a systematic review and meta-analysis of randomised controlled trials
Source: Br J Anaesth. 2025 Sep 30;135(6):1741–52. doi: 10.1016/j.bja.2025.09.013 (PMC12799406; doi:10.1016/j.bja.2025.09.013)
Supplement: Multimedia component 5 [file mmc5.docx]

**Appendix 5: Details of studies included in qualitative synthesis**

| Author | Year | Study Size | Benzodiazepine Arm | Comparator Arm | Surgery | Outcomes | Format of Outcome | Summary of Data | |
| --- | --- | --- | --- | --- | --- | --- | --- | --- | --- |
|  |  |  |  |  |  |  |  | Benzodiazepine Arm | Comparator Arm |
| Aanta, 1991 | 1991 | 107; 36//36/35 | Midazolam 0.08 mg/kg IM preoperatively | Saline preoperatively; Dexmedetomidine 1 mg/kg IM preoperatively | D&C | Pain | VAS Data not reported  in paper | — | — |
| Abdellatif, 2012 | 2012 | 54; 27//27 | Midazolam 0.01–0.03 mg/kg IV bolus, followed by infusion at 0.04 mg/kg/h intraoperatively | Dexmedetomidine 1 µg/kg IV bolus, followed by infusion at 0.4 µg/kg/h (range: 0.1–1 µg/kg/h) intraoperatively | Simple middle ear surgeries | Satisfaction | Qualitative scale data  (excellent, good, fair,  poor) | Excellent 2 (7.4)%  Good 6 (22.2%)  Fair 12 (44.4%)  Poor 7 (25.9%) | Excellent 21 (77.8%)  Good 5 (18.5%)  Fair 1 (3.7%)  Poor 0 (0%) |
| Abdelrady, 2024 | 2024 | 85; 42//43 | Midazolam 2 mg + 0.5% isobaric levobupivacaine 10 mg intrathecal intraoperatively | Fentanyl 25 µg + and 0.5% levobupivacaine 10 mg intrathecal intraoperatively | Elective C-section | Pain; Satisfaction | Satisfaction: Likert scale  Pain: VAS Data not reported in paper | Satisfactory: 90% | Satisfactory: 82.5% |
| Bagchi, 2014 | 2014 | 103; 51//51 | Midazolam bolus 0.05 mg/kg, followed by infusion at 0.06 mg/kg/h intraoperatively | Propofol bolus 1 mg/kg, followed by infusion at 3 mg/kg/h IV intraoperatively | Elective infraumbilical surgeries | Satisfaction | Data uses 7-point likert  scale | Score of 5: 7 (13.7%)  Score of 6: 17 (33.3%)  Score of 7: 36 (69.2%) | Score of 5: 3 (5.8%)  Score of 6: 13 (25.0%)  Score of 7: 36 (69.2%) |
| Bond, 1976 | 1976 | 60; 30//30 | Diazepam 0.2 mg/kg and pentazocine 0.5 mg/kg IV intraoperatively | Droperidol 0.1 mg/kg and fentanyl 0.005 mg/kg IV intraoperatively | Mix of mainly general, gynecologic, and orthopedic procedures | Pain | Qualitative scale data (none, mild, moderate, severe) | None: 22  Mild: 4  Moderate: 3  Severe: 1 | None: 20  Mild: 4  Moderate: 3  Severe: 3 |
| Chen, 2024 | 2024 | 80; 40//40 | Remimazolam initial bolus of 0.03 mg/kg/min, then repeated doses of 0.2–0.5 mg/kg/h IV intraoperatively | Dexmedetomidine initial bolus of 0.3 µg/kg, then additional doses of 0.2–1.0 µg/kg/h IV intraoperatively | Orthopedic surgery | Satisfaction | Qualitative scale data  (not satisfied, general satisfaction, satisfied, very satisfied) | Not satisfied: 2.5%  General satisfaction: 20%  Satisfied: 65%  Very satisfied: 12.5% | Not satisfied: 7.5%  General satisfaction: 52.5%  Satisfied: 32.5%  Very satisfied: 7.5% |
| El-Deeb, 2011 | 2011 | 120; 40//40/40 | Midazolam 2 mg + ondansetron 4 mg IV intraoperatively | Saline placebo; Dexamethasone 8 mg + ondansetron 4 mg intraoperatively | Elective thyroid | Pain; Satisfaction (in MA) | VAS data not reported in paper | — | — |
| Ellingson, 1977 | 1977 | 26; 13//13 | 30 mg diazepam IV intraoperatively | Ketamine 2 mg/kg IV intraoperatively | Forceps delivery | QoR | Classified patients into two groups: unpleasant vs. pleasant | Pleasant: 13 (100%)  Unpleasant: 0 (0%) | Pleasant: 6 (46.2%)  Unpleasant: 7 (53.8%) |
| Fragen, 1976 | 1976 | 95; 32/32//31 | Lorazepam 4 mg IM preoperatively; Diazepam 10 mg IM preoperatively | Placebo | NR | Satisfaction | Qualitative scale data (excellent, good, fair, poor) | Lorazepam:  Excellent: 15 (51.7%)  Good: 11 (37.9%)  Satisfactory: 2 (6.9%)  Unsatisfactory: 1 (3.4%)  Diazepam:  Excellent: 8 (27.6%)  Good: 15 (51.7%)  Satisfactory: 2 (69.0%)  Unsatisfactory: 4 (13.8%) | Excellent: 5 (17.2%)  Good: 10 (34.4%)  Satisfactory: 1 (3.4%)  Unsatisfactory: 9 (31.0%) |
| Franssen, 1993 | 1993 | 68; 34//34 | Alprazolam 1 mg PO preoperatively | Hydroxyzine 75 mg PO preoperatively | Plastic or orthopaedic surgery | Anxiety (in MA)  Satisfaction | Qualitative scale data (satisfaction) | Satisfactory: 25 (73.5%) | Satisfactory: 20 (64.5) |
| Fredman, 1999 | 1999 | 89; 29/30//30 | Midazolam 0.5 mg IV preoperatively; Midazolam 2 mg IV preoperatively | Placebo | Transurethral procedures | Anxiety (in MA); Satisfaction | Qualitative scale data (satisfaction); not reported in paper | “Patient satisfaction with their anesthetic experience was unaffected by the premedication.” | |
| Ganguly, 2016 | 2016 | 64; 32//32 | Midazolam 0.05 mg/kg loading dose followed by maintenance with 0.05 mg/kg/h IV intraoperatively | Dexmedetomidine 0.5 μg/kg loading dose followed by maintenance with 0.5 μg/kg/h IV intraoperatively | Infraumbilical surgeries | Satisfaction | Data uses 7-point likert scale | Score of 5: 2 (6.25%)  Score of 6: 9 (28.12%)  Score of 7: 21 (65.62%) | Score of 5: 3 (9.37%)  Score of 6: 10 (31.25%)  Score of 7: 19 (59.37%) |
| Gao, 2022 | 2022 | 50; 25//25 | Midazolam 0.1 mg/kg bolus preoperatively + dexmedetomidine 1 µg/kg over a 10 min and 0.5 µg/kg/h IV intraoperatively | Dexmedetomidine 1 µg/kg over 15 min preoperatively + dexmedetomidine 0.2-1 µg/kg/h IV + propofol 250 μg/kg/min for 15 min then a basal infusion dose of 50 μg/kg/min intraoperatively | Major abdominal surgery (gastric surgery, colectomy, pancreatic surgery, hepatic resection, esophageal resection) | Pain | VAS Data presented as dichotomous data | 50 mm < 16 (64%)  50 mm ≥ 9 (36%) | 50 mm < 14 (56%)  50 mm ≥ 11 (44%) |
| Gilliland, 1996 | 1996 | 45; 23//22 | Induction dose of midazolam 5 mg/70 kg followed after 60 minutes by an IV loading dose infusion of midazolam at 1 mg/70 kg/h over the next 48h | Placebo | Elective abdominal hysterectomy | Pain | Reported pain on a four-point scale (0 = no pain. 1 = mild pain, 2 = moderate pain and 3 = severe pain) | Data not reported  “The pain scores in patients receiving the midazolam infusion were lower than those in the control group over the first 24 h” | |
| Haram, 1981 | 1981 | 82; 39//43 | Diazepam 0.3 mg/kg IV intraoperatively | Thiopentone 3 mg/kg IV intraoperatively | C-section | QoR | QoR data reported as “Interview (satisfactory, unpleasant, uncertain); better, worse, uncertain, Y/N” | Satisfactory: 39 (100%)  Unpleasant: 0 (0%)  Uncertain: 0 (0%) | Satisfactory: 21 (52.5%)  Unpleasant: 13 (32.5%)  Uncertain: 6 (15%) |
| Hu, 2020 | 2020 | 105; 35//35/35 | Midazolam 0.02 mg/kg IV intraoperatively | Dexmedetomidine 1 µg/kg IV intraoperatively; Saline IV intraoperatively | C-section | Satisfaction | Qualitative scale data (excellent, good, fair, poor) | Very satisfied: 13 (37%)  Satisfied: 11 (32%)  Not satisfied: 11 (32%) | Dexmedetomidine:  Very satisfied: 23 (66%)  Satisfied: 10 (31%)  Not satisfied: 2 (6%)  Placebo:  Very satisfied: 5 (14%)  Satisfied: 11 (32%)  Not satisfied: 19 (54%) |
| Ibrahim, 2001 | 2001 | 141; 48//93 | Midazolam infusion | Sevoflurane titrated to a MAC of 1.0 | NR | Satisfaction | Satisfaction outcome dichotomous (pleased/not pleased). | Satisfaction:  Pleased: 100% | Satisfaction:  Pleased: 99% |
| Kapdi, 2021 | 2021 | 100; 50//50 | Midazolam 1 mg + 0.5% bupivacaine 10 mg intrathecal intraoperatively | Nalbuphine 0.75 mg + 0.5% bupivacaine 10 mg intrathecal intraoperatively | C-section | Pain | VAS data not reported in paper | – | – |
| Kim, 2013 | 2013 | 94; 32/32//30 | Midazolam 75 µg/kg IV bolus intraoperatively; Midazolam 75 µg/kg + ramosetron 0.3 mg IV bolus intraoperatively | Ramosetron 0.3 mg IV bolus intraoperatively | Thyroidectomy | Pain | Data described in paper but not reported | “There were no significant differences in the severity of postoperative pain between the groups” | |
| Liu, 2023 | 2023 | 136; 30//30/30/30/16 | Midazolam 0.03 mg/kg IV intraoperatively | Normal saline 0.1 ml/kg 15 min pre-anaesthesia 0.125 ml/kg/h maintenance; Dexmedetomidine (DEX) 0.25 μg/kg 15 min pre-anaesthesia + 0.5 μg/kg/h (maintenance dose); DEX 0.5 μg/kg 15 min pre-anaesthesia + maintenance dose; DEX 0.75 μg/kg 15 min pre-anaesthesia + maintenance dose | Hip replacement surgery | Pain | VAS pain data presented as dichotomous data (e.g., VAS 4-6: n%) | Midazolam:  ≤ 3  Baseline: 30 (100%)  Awakening: 30 (100%)  1 hr: 28 (93.3%)  2 hr: 26 (86.7%)  6 hr: 23 (76.7%)  4–6  Baseline: 0 (0%)  Awakening: 0 (0%)  1 hr: 2 (6.7%)  2 hr: 4 (13.3%)  6 hr: 7 (23.3%) | Normal Saline:  ≤ 3  Baseline: 30 (100%)  Awakening: 30 (100%)  1 hr: 25 (83.3%)  2 hr: 21 (70%)  6 hr: 17 (56.7%)  4–6  Baseline: 0 (0%)  Awakening: 0 (0%)  1 hr: 5 (126.7%)  2 hr: 9 (30%)  6 hr: 13 (43.3%)  Dexmedetomidine 0.25:  ≤ 3  Baseline: 30 (100%)  Awakening: 30 (100%)  1 hr: 29 (96.7%)  2 hr: 28 (93.3%)  6 hr: 28 (93.3%)  4–6  Baseline: 0 (0%)  Awakening: 0 (0%)  1 hr: 1 (3.3%)  2 hr: 2 (6.7%)  6 hr: 2 (6.7%)  Dexmedetomidine 0.5:  ≤ 3  Baseline: 30 (100%)  Awakening: 30 (100%)  1 hr: 29 (96.7%)  2 hr: 28 (93.3%)  6 hr: 28 (93.3%)  4–6  Baseline: 0 (0%)  Awakening: 0 (0%)  1 hr: 1 (3.3%)  2 hr: 2 (6.7%)  6 hr: 2 (6.7%)  Dexmedetomidine 0.75:  ≤ 3  Baseline: 15 (93.8%)  Awakening: 16 (100%)  1 hr: 16 (100%)  2 hr: 16 (100%)  6 hr: 16 (100%)  4–6  Baseline: 1 (6.3%)  Awakening: 0 (0%)  1 hr: 0 (0%)  2 hr: 0 (0%)  6 hr: 0 (0%) |
| McAteer, 1984 | 1984 | 150; 50/50//50 | Midazolam 5-7.5 mg based on weight range IV preoperatively; Midazolam 5-7.5 mg based on weight range + atropine 0.6 mg IV preoperatively | 1-2 mL of mixture of papaveretum 20 mg + hyoscine 0.4 mg IV preoperatively | Gynaecological surgery | Anxiety; Satisfaction | Anxiety data presented as ordinal data. Satisfaction data presented as dichotomous data. | Anxiety: VAS  Midazolam:  Pre- premedication: 4.1 (2.5)  Pre- induction of anesthesia: 2.3 (1.8)  24 hr: 1.6 (2.2)  Midazolam + atropine:  Pre- premedication: 4.1 (2.5)  Pre- induction of anesthesia: 2.3 (1.8)  24 hr: 1.6 (2.2) | Anxiety: VAS  Pre- premedication: 4.6 (2.8)  Pre- induction of anesthesia: 2.3 (2.1)  24 hr: 2.4 (2.7) |
|  |  |  |  |  |  |  |  | Satisfaction:  Premedication “very good”  Group A (midazolam): 62%  Group C (comparator): 58%  Premedication “unsatisfactory”  Group A (midazolam): 8%  Group C (comparator): 18%  These differences were not significant. | |
| Moon, 2018 | 2018 | 37; 19//18 | Midazolam 1-2 mg bolus, 1-2 maintenance 30 min IV intraoperatively | 30-min VR program ( underwater view of the ocean while listening to narrations designed to induce relaxation and meditation) intraoperatively | Urologic surgery | Satisfaction | Median (IQR) of ordinal data e.g., (1= extremely dissatisfied, 2=dissatisfied, 3=undecided, 4=satisfied, 5=extremely satisfied) | 5 (4–5) | 5 (5–5) |
| Naguib, 2000 | 2000 | 84; 12/12/12//12/12/12/12 | Midazolam 0.05 mg/kg sublingual preoperatively; Midazolam 0.1 mg/kg sublingual preoperatively; Midazolam 0.2 mg/kg sublingual preoperatively | Melatonin 0.05 mg/kg sublingual preoperatively; Melatonin 0.1 mg/kg sublingual preoperatively; Melatonin 0.2 mg/kg sublingual preoperatively; Saline sublingual preoperatively | Gynecological laparoscopic procedures | Anxiety | Data available but missing SD. | “There were no differences in anxiety VAS between the groups after surgery” | |
| Pyeon, 2017 | 2017 | 76; 25/27//24 | Triazolam 0.25 mg PO preoperatively; Triazolam 0.375 mg PO preoperatively | No medication preoperatively | Breast, thyroid, abdomen | Satisfaction | Ordinal data (0=unsatisfactory, 3=excellent) | Triazolam 0.25:  Baseline: 0.8 ± 0.6  In OR: 0.8 ± 0.6  Triazolam 0.375:  Baseline: 0.9 ± 1.0  In OR: 1.1 ± 0.8 | Control:  Baseline: 0.8 ± 0.8  In OR: 1.5 ± 1.0 |
| Roberts, 1976 | 1976 | 44; 17//27 | Diazepam 7.5 mg IM preoperatively | Morphine sulfate 10 mg + scopolamine 0.4 mg IM preoperatively | NR | Satisfaction | Ordinal data | 24 hour:  Liked it real well: 11 (25%)  Did not like it very well: 3 (7%)  Would like to have it again if necessary 11 (25%)  48 hour:  Liked it real well: 14 (32%)  Did not like it very well: 4 (9%)  Would like to have it again if necessary 10 (23%) | 24 hour:  Liked it real well: 6 (14%)  Did not like it very well: 2 (5%)  Would like to have it again if necessary 11 (25%)  48 hour:  Liked it real well: 5 (11%)  Did not like it very well: 1 (2%)  Would like to have it again if necessary 9 (20%) |
| Russell, 1983 | 1983 | 42; 22//20 | Lorazepam 2.5-5 mg PO preoperatively with dose determined by weight category. | Placebo PO preoperatively | TURP | Anxiety | Categorical data (change in VAS scores, timepoint: preop + intraop) | Anxiety greater at operation: 12  Same: 2  Less at operation: 8 | Anxiety greater at operation: 11  Same: 1  Less at operation: 8 |
| Seow, 1985 | 1985 | 21; 11//10 | Diazepam loading dose of 20 ( ± 15) mg at a rate of 1 mg/min intraoperatively | 0.8% Chlormethiazole 10 mL/min infusion over 16 (±6) min intraoperatively | Lower limb and lower abdominal surgery | Satisfaction | Dichotomous data | “All patients expressed satisfaction with the  regional blockade plus sedation.” | |
| Sharan, 2016 | 2016 | 60; 30//30 | Midazolam 0.05 mg/kg followed by 2 mg increments until an adequate level of sedation was reached intraoperatively | Propofol 1–2 mg/kg to a maximum of 2 mg/kg followed by 20 mg increments if needed intraoperatively | Elective surgery | Satisfaction | Ordinal data | Patient satisfaction score:  1: 18 (60%)  2: 11 (36.7%)  3: 30 (100%) | Patient satisfaction score:  1: 27 (90%)  2: 3 (10%)  3: 0 (0%) |
| Silva-Jr, 2019 | 2019 | 120; 53//67 | Midazolam 0.02 mg/kg bolus dose + 0.5 fentanyl µg/kg bolus dose intraoperatively | Dexmedetomidine 1μg/kg bolus dose + 0.2-0.8μg/kg/h infusion intraoperatively | Urologic, orthopedics, vascular, GI, gynecologic | Pain | Measured VAS but presented in paper as ordinal data | Moderate/Severe pain:  24 hr: 18 (34%)  48 hr: 18 (33%) | Moderate/Severe pain:  24 hr: 17 (25%)  48 hr: 12 (18%) |
| Song, 2022 | 2022 | 130; 65//69 | Midazolam 0.05 mg/kg IM preoperatively | Normal saline placebo 0.01 mg/kg IM preoperatively | Gynecologic, GI, orthopedic, ENT, other | Pain (in meta-analysis); Satisfaction | Qualitative data (satisfaction) | Overall Satisfaction:  57 (87.7%) | Overall Satisfaction:  46 (66.7%) |
| van Wijhe, 1985 | 1985 | 203; 67//69/67 | Midazolam 70 μg/kg IM preoperatively | Normal saline IM preoperatively; Fentanyl 1.4 μg/kg + droperidol 70 μg/kg IM preoperatively | Orthopedic surgery | Anxiety | VAS 0-5 at POD2 reported in figure, unable to combine. | The decrease in anxiety level was greater in the midazolam group than in the fentanyl/droperidol and placebo groups (P<0.00l), according to the patient. There was no difference between the fentanyl/droperidol and placebo groups. | |
| Wallace, 1984 | 1984 | 90; 30//30/29 | Lorazepam 0.05 mg/kg (maximum 4 mg) IM preoperatively | Hydroxyzine 1.5 mg/kg (maximum, 100 mg) IM preoperatively; 1 ml of normal saline IM preoperatively | NR | Anxiety | Anxiety was evaluated at each evaluation time with  a Clinical Global Impression Scale rating of none,  borderline, mild, moderate, marked, severe, or extreme. | At least 85% of the patients experienced no  anxiety, borderline, or mild anxiety at 24h. Of the remaining patients, one lorazepam-treated patient reported marked anxiety at 24h and the others reported moderate anxiety. | |
| Wender, 1977 | 1977 | 140; 35/35//35/35 | Diazepam 7.5 mg IV preoperatively; Diazepam 15 mg IV preoperatively | Hydroxyzine 75 mg IV preoperatively; Hydroxyzine 150 mg IV preoperatively | NR | Satisfaction | Ordinal data reported (e.g., 0=poor, 3=excellent). | Satisfaction:  Diazepam 7.5:  Poor- 2 (6%)  Fair- 10 (29%)  Good- 14 (41%)  Excellent- 8 (24%)  Diazepam 15:  Poor- 2 (6%)  Fair- 4 (12%)  Good- 19 (58%)  Excellent- 8 (24%) | Satisfaction:  Hydroxyzine 75 mg:  Poor- 7 (21%)  Fair- 7 (21%)  Good- 14 (42%)  Excellent- 5 (15%)  Hydroxyzine 10 mg:  Poor- 7 (22%)  Fair- 3 (9%)  Good- 16 (50%)  Excellent- 6 (19%) |
| Yamakage, 2002 | 2002 | 48; 12//12/12/12 | Triazolam 0.25 mg PO preoperatively | Control- no premedication; Zopiclone 7.5 mg PO preoperatively; Clonidine 0.15 mg PO preoperatively; | NR | Patient Acceptance | Qualitative scale (Y/N) data: acceptability of possible repeat anesthesia, acceptability of smell | Acceptability of the smell: 8 (67%)  Acceptability of possible repeat anesthesia: 9 (75%) | Control:  Acceptability of the smell: 5 (42%)  Acceptability of possible repeat anesthesia: 7 (58%)  Zopiclone:  Acceptability of the smell: 10 (83%)  Acceptability of possible repeat anesthesia: 10 (83%)  Clonidine:  Acceptability of the smell: 9 (75%)  Acceptability of possible repeat anesthesia: 11 (92%) |

SD: standard deviation; IM: intramuscular; D&C: dilation and curettage; VAS: visual analogue scale; IV: intravenously; PO: orally; MA: meta-analysis; QoR: quality of recovery; NR: not reported; TURP: transurethral resection of the prostate; MAC: minimum alveolar concentration; GI: gastrointestinal; ENT: ear nose and throat
