# Supplementary material for: Effects of perioperative benzodiazepine administration on postoperative patient-reported outcomes: a systematic review and meta-analysis of randomised controlled trials
Source: Br J Anaesth. 2025 Sep 30;135(6):1741–52. doi: 10.1016/j.bja.2025.09.013 (PMC12799406; doi:10.1016/j.bja.2025.09.013)
Supplement: Multimedia component 6 [file mmc6.pdf]

Appendix 6: Detailed risk of bias of included trials

Study	D1	D2	D3	D4	D5	D6	D7
Aanta 1991	Probably low	Low	Low	Low	Low	Low	Probably low
Abdelemam 2022	Low	Low	Low	Low	Low	Low	Low
Abdellatif 2012	Low	Low	Low	Low	Low	Low	Low
Abdelrady 2022	Low	Low	Low	Low	Low	Low	Low
Abhishek 2022	Low	Probably high	Low	Low	Low	Low	Low
Aldrich 2022	Low	Probably high	Low	Low	Low	Low	Low
Aleniewski 1977	Probably low	Low	Low	Low	Probably high	Low	Low
Amin 2022	Low	Low	Low	Low	Low	Low	Low
Amin 2019	Low	Low	Low	Low	Low	Low	Low
Ammar 2018	Low	Low	Low	Low	Low	Low	Low
Anand 2015	Low	Low	Low	Low	Low	Low	Low
Arifin 2023	Low	Low	High	High	Low	Low	Low
Ashok 2023	High	High	High	High	Low	Low	Low
Bagchi 2014	Low	Probably high	High	Probably low	Low	Low	Low
Basuni 2016	Low	Low	Probably low	Low	Low	Low	Low
Bindra 2010	Low	Low	Low	Low	Low	Low	Low
Bishnoi 1998	Low	Probably high	Low	Low	Low	Low	Low
Bond 1976	High	Probably high	High	Probably low	Low	High	Low
Borracci 2013	Low	Low	Low	Low	Low	Low	Low
Caumo 2002	Low	Probably high	Low	Low	Low	Low	Low
Chandra 2024	Low	Probably low	Low	Low	Low	Low	Low
Chen 2024	Probably low	High	High	High	Low	Low	Low
Choi 2021	Low	Low	Probably low	Probably low	Low	Low	Low
Choi 2022	Low	Low	Probably low	Low	Low	Low	Low
Dash 2024	Low	Low	Low	Low	Low	Low	Low
Dyck 1991	Low	Probably high	Probably high	High	Low	Low	Low
El-Baradey 2014	Probably low	Low	High	High	Low	Low	Low
El-Deeb 2011	Low	Low	Probably high	Low	Low	Probably low	Low
Ellingson 1977	Probably low	Probably high	High	Probably low	Low	Low	Low
Elvir Lazo 2016	Low	Low	Low	Low	Low	Low	Low
Ersoy 2015	Low	Probably high	Probably low	Low	Low	Low	Low
Feng 2019	Probably low	High	High	High	Low	Low	Low
Fragen 1976	Probably low	Probably high	Probably low	Low	Low	Low	Low
Franssen 1993	Probably low	Probably high	High	Low	Low	Low	Low
Fredman 1999	Low	Low	Low	Low	Low	Low	Low
Gale 1976	Low	Probably high	Probably low	Low	Low	Low	Low
Ganguly 2016	Low	Low	Probably low	Low	Low	Low	Low
Gao 2022	Low	Probably low	Probably low	Low	Low	Low	Low
Gilliland 1996	Low	Low	Low	Low	Low	Low	Low
Giordano 2023	Low	Probably high	High	Low	High	Low	Low
Ha 2007	Probably low	Probably high	Probably low	Low	Probably low	Probably low	Probably low
Haram 1981	Probably low	High	High	Probably low	High	Low	Low
Hashemian 2023	Probably low	Probably high	Probably low	Low	Low	Low	Low
He 2024	Low	High	High	High	Low	Low	Low
Honarmand 2012	Low	Low	Low	Low	Low	Low	Low
Hong 2021	Low	Probably high	Low	Low	Low	Low	High
Hong 2024	Low	Probably low	Low	Low	Low	Low	Low
Hu 2020	Low	Probably high	Low	Low	Low	Low	Low
Ibrahim 2001	Probably low	Probably low	Low	Low	Probably high	Low	Low
Ionescu 2008	Probably low	Low	Low	Low	Low	Low	Low
Jabalameli 2012	Low	Low	Low	Low	Low	Low	Low
Kain 2001	Low	Low	Probably high	High	High	Low	Low
Kang 2023	Low	Low	Low	Low	Low	Low	Low
Kapdi 2021	High	Low	Probably low	Low	Low	Low	Low
Karbasfrushan 2012	Probably low	Low	Low	Low	Low	Low	Low
Keerthy 2022	Probably low	Probably high	High	Low	Low	Low	Low
Kestin 1999	Probably low	Low	Low	Low	Low	Low	Low
Kim 2001	Probably low	Probably high	Low	Low	Probably low	Low	Low
Kim 2010	Probably low	Low	Low	Low	Low	Low	Low
Kim 2011	Low	Probably low	Low	Low	Low	Low	Low
Kim 2013	Low	Low	Low	Low	Low	Low	Low
Kim 2017	Low	Low	Low	Low	Low	Low	Low
Kim 2024	Low	Low	Low	Low	Low	Low	Low
Kotani 2024	Low	High	Low	Low	Low	Low	Low
Kowark 2024	Low	Low	Low	Low	Low	Low	Low
Kulkarni 2022	Probably low	Probably high	High	Low	Low	Low	Low
Kumar 2020	Probably low	Low	Low	Low	Low	Low	Low
Kwak 2008	Low	Low	Low	Low	Low	Low	Low
Lal 2023	Low	High	High	Low	Low	Low	Low
Lee 2007	Low	Probably high	Low	Low	Low	Low	Low
Lee 2023	Low	Probably high	Probably low	Low	Low	Low	Low
Lee 2024 (1)	Low	High	Low	Low	Low	Low	Low
Lee 2024 (2)	Low	Low	Low	Low	Low	Low	Low
Lee 2024 (3)	Low	Low	Low	Low	Low	Low	Low
Liang 2024	Low	Low	Low	Low	Low	Low	Low
Liu 2023	Low	Low	Probably low	Low	Low	Low	Low
Liu 2024	Low	Low	Low	Low	Low	Low	Low
Ma 2021	Low	Probably high	High	Low	Probably low	Low	Low
Mao 2022	Low	Probably high	Low	Low	Low	Low	Low
Maurice-Szamburski 2015	Low	Low	Low	Low	Probably low	Low	Low
McAteer 1984	Probably low	Probably high	Low	Low	Low	Low	Low
Meena 2021	Low	Low	Low	Low	Low	Low	Low
Modir 2022	Low	Probably high	Low	Low	Low	Low	Low
Mokhtar 2016	Low	Low	Low	Low	Low	Low	Low
Moon 2018	Low	Low	High	High	Low	Low	Low
Moslemi 2023	Low	High	Low	Low	Low	Low	Low
Moutzouros 2021	Low	Probably high	High	High	Low	Low	Low
Naguib 1999	Probably low	Probably high	Low	Low	High	High	Low
Naguib 2000	Probably low	Probably high	Low	Low	High	High	Low
Nanjegowda 2011	Low	Probably high	Low	Probably low	Low	Low	Low
Nayak 2023	Low	Probably high	Probably low	Low	Low	Low	Low
Nizamuddeen 2023	Low	Low	High	High	Low	Low	Low
Park 2015	Low	Probably high	Probably low	Probably low	Low	Low	Low
Parkih 2013	Low	Probably low	Low	Low	Low	Low	Low
Prasad 2023	Low	Low	High	High	Low	Low	Low
Przesmycki 2011	Low	Probably high	Probably low	Probably low	Low	Low	Low
Pyeon 2017	Low	Probably high	High	High	High	Low	Low
Rao 2024	Probably low	Probably high	Probably high	Probably high	Low	Low	Low
Roberts 1976	Low	Probably high	High	High	Low	Low	High
Russel 1983	Probably low	Probably high	Low	Low	Low	Low	Low
Ryu 2024	Low	Probably high	Low	Low	Low	Low	Low
Senses 2013	Low	Low	Low	Probably low	Low	Low	Low
Seow 1985	Probably low	Probably high	High	High	Low	Low	Low
Sharan 2016	Probably low	Probably high	High	High	Low	Low	Low
Siddiqui 2022	Low	Probably high	Probably low	Probably low	Low	Low	Low
Silva-JR 2019	Low	Probably low	Low	Low	Low	Low	Low
Singhal 2024	Low	Low	Low	Low	Low	Low	Low
Sirmela 2023	Low	Low	Low	Low	Low	Low	Low
Song 2022 (1)	Low	Low	Probably high	Low	Low	Low	Low
Song 2022 (2)	Low	Low	Low	Low	Low	Low	Low
Sun 2024	Probably low	Probably high	High	High	Low	Low	Low
vanBeek 2020	Low	Low	Low	Low	Low	Low	Low
vanWijhe 1985	Probably low	Low	Low	Low	Low	Low	Low
Wallace 1884	Probably low	Probably high	Probably low	Probably low	Low	Low	Low
Wang 2023	Low	Low	Low	Low	Low	Low	Low
Wender 1977	Probably low	Probably high	Probably low	Probably low	Low	Low	Low
Xu 2020	Low	Low	Low	Low	Low	Low	Low
Yamakage 2002	Low	Probably high	High	High	Low	Low	Probably low
Yegin 2024	Probably low	Low	Low	Low	Low	Low	Low
Yoo 2024	Low	Probably high	Low	Low	Low	Low	Low
Yue 2023	Low	Probably high	Probably low	Probably low	Low	Low	Low
Zakeri 2017	Low	Probably high	High	High	Low	Low	Low
Zhou 2024	Low	Low	Low	Low	Low	Low	Low

= Low
= Probably low
= Probably high
= High

**D1**	Random sequence generation
**D2**	Allocation concealment
**D3**	Blinding of participants
**D4**	Blinding of outcome assessment
**D5**	Incomplete outcome data
**D6**	Selective reporting
**D7**	Other bias
