# Supplementary material for: Effects of perioperative benzodiazepine administration on postoperative patient-reported outcomes: a systematic review and meta-analysis of randomised controlled trials
Source: Br J Anaesth. 2025 Sep 30;135(6):1741–52. doi: 10.1016/j.bja.2025.09.013 (PMC12799406; doi:10.1016/j.bja.2025.09.013)
Supplement: Multimedia component 7 [file mmc7.pdf]

Appendix 7(a): The effect of perioperative benzodiazepines on immediate postoperative patient-reported pain

| | Benzodiazepine | | Comparison | | | |
|---|---|---|---|---|---|---|
| Study | N | Mean (SD) | N | Mean (SD) | MD (95% CI) | %Weight |
| Kain 2001 | 25 | 3.77 (0.63) | 29 | 3.52 (0.49) | 0.25 (-0.06, 0.55) | 2.02 |
| Kim 2001 | 30 | 3.50 (0.66) | 15 | 3.50 (0.92) | 0.00 (-0.52, 0.52) | 1.85 |
| Caumo 2002 | 56 | 2.66 (0.90) | 56 | 1.41 (1.05) | 1.25 (0.89, 1.61) | 1.98 |
| Yegin 2004 | 22 | 1.09 (0.61) | 22 | 1.04 (0.72) | 0.05 (-0.34, 0.44) | 1.96 |
| Lee 2007 | 44 | 1.50 (0.60) | 44 | 1.60 (0.60) | -0.10 (-0.35, 0.15) | 2.05 |
| Ha 2007 | 41 | 2.30 (1.90) | 78 | 2.30 (1.90) | 0.00 (-0.72, 0.72) | 1.66 |
| Kwak 2008 | 21 | 2.43 (1.18) | 21 | 2.96 (1.05) | -0.53 (-1.20, 0.15) | 1.70 |
| Kim 2010 | 30 | 2.58 (1.51) | 30 | 2.35 (1.11) | 0.23 (-0.44, 0.90) | 1.71 |
| Bindra 2010 | 40 | 2.52 (0.90) | 40 | 2.69 (1.05) | -0.17 (-0.60, 0.26) | 1.93 |
| Ma 2011 | 130 | 1.45 (0.36) | 130 | 2.10 (0.44) | -0.65 (-0.75, -0.55) | 2.11 |
| Nanjegowda 2011 | 25 | 3.36 (0.47) | 25 | 5.66 (0.86) | -2.30 (-2.69, -1.91) | 1.96 |
| Honarmand 2012 | 40 | 0.50 (0.76) | 40 | 0.90 (0.90) | -0.40 (-0.77, -0.03) | 1.98 |
| Jabalameli 2012 | 88 | 0.80 (1.83) | 44 | 1.10 (2.20) | -0.30 (-1.05, 0.45) | 1.62 |
| Karbasfrushan 2012 | 62 | 3.24 (1.90) | 62 | 7.03 (2.29) | -3.79 (-4.53, -3.05) | 1.64 |
| Borracci 2013 | 28 | 4.04 (1.92) | 32 | 2.12 (1.24) | 1.91 (1.08, 2.74) | 1.54 |
| El-Baradey 2014 | 20 | 4.14 (1.30) | 40 | 4.00 (2.02) | 0.14 (-0.71, 0.99) | 1.53 |
| Park 2015 | 25 | 3.27 (2.40) | 23 | 2.85 (3.26) | 0.42 (-1.21, 2.05) | 0.87 |
| Anand 2015 | 30 | 4.05 (1.54) | 30 | 3.59 (1.30) | 0.46 (-0.26, 1.18) | 1.66 |
| Zakeri 2017 | 31 | 3.99 (0.20) | 62 | 3.85 (0.20) | 0.14 (0.05, 0.23) | 2.11 |
| Ammar 2018 | 30 | 2.23 (1.15) | 30 | 2.41 (1.30) | -0.18 (-0.80, 0.44) | 1.76 |
| Feng 2019 | 124 | 3.11 (0.41) | 124 | 5.46 (1.12) | -2.35 (-2.56, -2.14) | 2.07 |
| Amin 2019 | 30 | 2.64 (0.80) | 60 | 3.70 (2.29) | -1.06 (-1.71, -0.41) | 1.73 |
| Xu 2020 | 43 | 2.00 (2.00) | 43 | 1.20 (0.90) | 0.80 (0.14, 1.46) | 1.72 |
| Maurice-Szamburski 2020 | 330 | 2.90 (2.32) | 641 | 3.60 (2.98) | -0.70 (-1.04, -0.36) | 2.00 |
| Kumar 2020 | 50 | 5.12 (0.90) | 50 | 4.30 (1.05) | 0.82 (0.44, 1.20) | 1.97 |
| Choi 2021 | 44 | 2.79 (0.76) | 44 | 2.04 (1.01) | 0.75 (0.38, 1.12) | 1.97 |
| Hong 2021 | 52 | 2.10 (1.20) | 52 | 2.01 (0.85) | 0.09 (-0.31, 0.49) | 1.95 |
| Przesmycki 2021 | 19 | 1.00 (0.40) | 54 | 1.00 (0.29) | 0.00 (-0.20, 0.20) | 2.08 |
| Aldrich 2022 | 60 | 3.00 (7.41) | 63 | 2.40 (7.41) | 0.60 (-2.02, 3.22) | 0.45 |
| Song 2022 | 65 | 3.80 (2.10) | 69 | 3.80 (2.10) | 0.00 (-0.71, 0.71) | 1.67 |
| Abdelemam 2022 | 56 | 1.50 (0.71) | 28 | 1.00 (0.50) | 0.50 (0.24, 0.76) | 2.05 |
| Modir 2022 | 35 | 3.45 (0.51) | 70 | 3.45 (0.51) | 0.00 (-0.21, 0.21) | 2.07 |
| Siddiqui 2022 | 34 | 2.87 (2.27) | 32 | 2.49 (2.32) | 0.38 (-0.73, 1.49) | 1.27 |
| Amin 2022 | 80 | 1.00 (1.30) | 40 | 1.00 (1.48) | 0.00 (-0.54, 0.54) | 1.83 |
| Nayak 2023 | 30 | 1.97 (0.75) | 30 | 0.93 (0.77) | 1.04 (0.66, 1.42) | 1.97 |
| Kang 2023 | 30 | 2.80 (1.40) | 30 | 3.00 (1.60) | -0.20 (-0.96, 0.56) | 1.62 |
| Prasad 2023 | 75 | 1.08 (0.90) | 150 | 0.70 (1.15) | 0.38 (0.11, 0.65) | 2.04 |
| Yue 2023 | 100 | 1.00 (0.33) | 100 | 1.00 (0.33) | 0.00 (-0.09, 0.09) | 2.11 |
| Wang 2023 | 88 | 2.26 (2.59) | 89 | 0.28 (0.86) | 1.98 (1.41, 2.55) | 1.81 |
| Moslemi 2023 | 35 | 2.00 (0.69) | 35 | 1.20 (1.30) | 0.80 (0.31, 1.29) | 1.88 |
| Sirmela 2023 | 25 | 2.72 (0.68) | 50 | 2.50 (0.58) | 0.22 (-0.09, 0.53) | 2.02 |
| Nizamuddeen 2023 | 30 | 3.63 (0.77) | 30 | 5.60 (0.97) | -1.97 (-2.41, -1.53) | 1.92 |
| Zhou 2024 | 120 | 0.00 (0.74) | 30 | 0.00 (0.63) | 0.00 (-0.26, 0.26) | 2.05 |
| Sun 2024 | 138 | 2.45 (0.38) | 46 | 3.04 (0.45) | -0.59 (-0.73, -0.45) | 2.10 |
| Yoo 2024 | 20 | 3.20 (1.60) | 20 | 3.00 (1.30) | 0.20 (-0.70, 1.10) | 1.47 |
| Hong 2024 | 35 | 1.10 (3.20) | 35 | 0.90 (2.80) | 0.20 (-1.21, 1.61) | 1.02 |
| Liu 2024 | 50 | 1.92 (0.73) | 50 | 2.08 (0.72) | -0.16 (-0.44, 0.12) | 2.03 |
| Lee 2024 | 36 | 2.00 (0.80) | 36 | 2.10 (0.70) | -0.10 (-0.45, 0.25) | 1.99 |
| Lee 2024 | 33 | 3.61 (0.70) | 65 | 3.75 (0.75) | -0.14 (-0.44, 0.16) | 2.02 |
| Singhal 2024 | 50 | 0.00 (2.22) | 50 | 0.00 (0.74) | 0.00 (-0.65, 0.65) | 1.73 |
| Rao 2024 | 28 | 0.00 (0.80) | 28 | 0.11 (0.32) | -0.11 (-0.43, 0.21) | 2.01 |
| Dash 2024 | 150 | 3.20 (1.40) | 150 | 4.10 (1.70) | -0.90 (-1.25, -0.55) | 1.99 |
| Chandra 2024 | 30 | 3.45 (0.73) | 30 | 2.03 (0.76) | 1.42 (1.04, 1.80) | 1.97 |
| Ashok 2024 | 40 | 1.55 (0.80) | 40 | 1.27 (0.91) | 0.28 (-0.10, 0.66) | 1.97 |
| Lee 2024 | 31 | 4.00 (1.48) | 32 | 4.00 (0.74) | 0.00 (-0.58, 0.58) | 1.79 |
| Overall, DL ($I^2$ = 95.9%, p < 0.001) | 3014 | | 3349 | | -0.05 (-0.25, 0.15) | 100.00 |

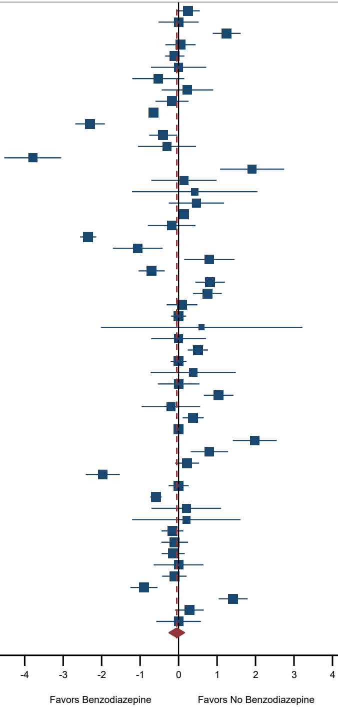


NOTE: Weights are from random-effects model

Appendix 7(b): Post hoc sensitivity analysis considering only comparisons to placebo or nothing

| Study | Benzodiazepine N | Benzodiazepine Mean (SD) | Comparison N | Comparison Mean (SD) | MD (95% CI) | %Weight |
|---|---|---|---|---|---|---|
| Aldrich 2022 | 60 | 3.00 (7.41) | 63 | 2.40 (7.41) | 0.60 (-2.02, 3.22) | 1.50 |
| Amin 2019 | 30 | 2.64 (0.80) | 60 | 3.70 (2.29) | -1.06 (-1.71, -0.41) | 4.10 |
| Amin 2022 | 80 | 1.00 (1.30) | 40 | 1.00 (1.48) | 0.00 (-0.54, 0.54) | 4.25 |
| Ammar 2018 | 30 | 2.23 (1.15) | 30 | 2.41 (1.30) | -0.18 (-0.80, 0.44) | 4.14 |
| Bindra 2010 | 40 | 2.52 (0.90) | 40 | 2.69 (1.05) | -0.17 (-0.60, 0.26) | 4.38 |
| Caumo 2002 | 56 | 2.66 (0.90) | 56 | 1.41 (1.05) | 1.25 (0.89, 1.61) | 4.45 |
| Choi 2021 | 44 | 2.79 (0.76) | 44 | 2.04 (1.01) | 0.75 (0.38, 1.12) | 4.44 |
| Dash 2024 | 150 | 3.20 (1.40) | 150 | 4.10 (1.70) | -0.90 (-1.25, -0.55) | 4.46 |
| Honarmand 2012 | 40 | 0.50 (0.76) | 20 | 0.70 (1.30) | -0.20 (-0.82, 0.42) | 4.15 |
| Hong 2021 | 52 | 2.10 (1.20) | 52 | 2.01 (0.85) | 0.09 (-0.31, 0.49) | 4.41 |
| Kain 2001 | 25 | 3.77 (0.63) | 29 | 3.52 (0.49) | 0.25 (-0.06, 0.55) | 4.50 |
| Karbasfrushan 2012 | 62 | 3.24 (1.90) | 62 | 7.03 (2.29) | -3.79 (-4.53, -3.05) | 3.97 |
| Kim 2001 | 30 | 3.50 (0.66) | 15 | 3.50 (0.92) | 0.00 (-0.52, 0.52) | 4.27 |
| Kim 2010 | 30 | 2.58 (1.51) | 30 | 2.35 (1.11) | 0.23 (-0.44, 0.90) | 4.07 |
| Kwak 2008 | 21 | 2.43 (1.18) | 21 | 2.96 (1.05) | -0.53 (-1.20, 0.15) | 4.06 |
| Maurice-Szamburski 2020 | 330 | 2.90 (2.32) | 641 | 3.60 (2.98) | -0.70 (-1.04, -0.36) | 4.47 |
| Nanjegowda 2011 | 25 | 3.36 (0.47) | 25 | 5.66 (0.86) | -2.30 (-2.69, -1.91) | 4.42 |
| Siddiqui 2022 | 34 | 2.87 (2.27) | 32 | 2.49 (2.32) | 0.38 (-0.73, 1.49) | 3.37 |
| Sirmela 2023 | 25 | 2.72 (0.68) | 25 | 2.72 (0.61) | 0.00 (-0.36, 0.36) | 4.45 |
| Song 2022 | 65 | 3.80 (2.10) | 69 | 3.80 (2.10) | 0.00 (-0.71, 0.71) | 4.01 |
| Sun 2024 | 138 | 2.45 (0.38) | 46 | 3.04 (0.45) | -0.59 (-0.73, -0.45) | 4.59 |
| Yegin 2004 | 22 | 1.09 (0.61) | 22 | 1.04 (0.72) | 0.05 (-0.34, 0.44) | 4.42 |
| Zakeri 2017 | 31 | 3.99 (0.20) | 31 | 2.99 (0.30) | 1.00 (0.87, 1.13) | 4.60 |
| Zhou 2024 | 120 | 0.00 (0.74) | 30 | 0.00 (0.63) | 0.00 (-0.26, 0.26) | 4.53 |
| Overall, DL | 1540 | | 1633 | | -0.26 (-0.65, 0.14) | 100.00 |
| ($I^2$ = 96.5%, $p < 0.001$) | | | | | | |

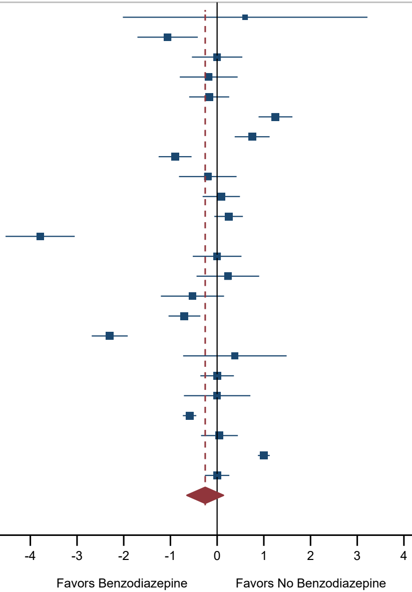


NOTE: Weights are from random-effects model
