# Supplementary material for: Effects of perioperative benzodiazepine administration on postoperative patient-reported outcomes: a systematic review and meta-analysis of randomised controlled trials
Source: Br J Anaesth. 2025 Sep 30;135(6):1741–52. doi: 10.1016/j.bja.2025.09.013 (PMC12799406; doi:10.1016/j.bja.2025.09.013)
Supplement: Multimedia component 8 [file mmc8.pdf]

Author(s):
Question: Benzodiazepines compared to other medications or placebo for perioperative quality of recovery
Setting:
Bibliography:

Certainty assessment							№ of patients		Effect		Certainty	Importance
**№ of studies**	**Study design**	**Risk of bias**	**Inconsistency**	**Indirectness**	**Imprecision**	**Other considerations**	**benzodiazepines**	**other medications or placebo**	**Relative (95% CI)**	**Absolute (95% CI)**		
**Postoperative pain (immediate postoperative)**												
56	randomised trials	not serious[a]	serious[b]	not serious	not serious[c]	none	3114	3449	-	MD **0.05 lower** (0.24 lower to 0.15 higher)	⨁⨁⨁◯ Moderate[a,b,c]	
**Postoperative pain (in hospital)**												
13	randomised trials	not serious[a]	serious[b]	not serious	not serious[c]	none	536	451	-	MD **0.1 lower** (0.38 lower to 0.19 higher)	⨁⨁⨁◯ Moderate[a,b,c]	
**Quality of recovery (immediate postoperative)**												
9	randomised trials	not serious[d]	serious[b]	not serious	not serious[c]	none	394	396	-	MD **1.05 lower** (5.83 lower to 3.73 higher)	⨁⨁⨁◯ Moderate[b,c,d]	
**Quality of recovery (in hospital)**												
5	randomised trials	not serious[d]	serious[b]	not serious	not serious[c]	none	288	286	-	MD **0.12 higher** (5.17 lower to 5.41 higher)	⨁⨁⨁◯ Moderate[b,c,d]	
**Satisfaction (immediate postoperative)**												
16	randomised trials	not serious[a]	very serious[e]	not serious	serious[f]	none	1209	1584	-	MD **3.37 lower** (7.81 lower to 1.08 higher)	⨁◯◯◯ Very low[a,e,f]	
**Postoperative anxiety (immediate postoperative)**												
22	randomised trials	not serious[a]	very serious[e]	not serious	not serious	none	996	1169	-	MD **2.18 higher** (1.05 higher to 3.3 higher)	⨁⨁◯◯ Low[a,e]	

CI: confidence interval; **MD:** mean difference

Explanations

a. Even though we identified concerns about risk of bias in the subgroup analysis did not support the presence of an interaction effect.
b. Important statistical heterogeneity but centred around the line of no effect.
c. The 95% confidence interval crossed the line of no effect but was narrower than the MCID for the outcome.
d. Only low risk of bias studies were included.
e. Important statistical heterogeneity and variability in how the intervention was delivered.
f. The 95% confidence interval did not exclude a clinically important different in postoperative patient satisfaction.
