# Supplementary figures and images for: Effects of perioperative benzodiazepine administration on postoperative patient-reported outcomes: a systematic review and meta-analysis of randomised controlled trials
Source: Br J Anaesth. 2025 Sep 30;135(6):1741–52. doi: 10.1016/j.bja.2025.09.013 (PMC12799406; doi:10.1016/j.bja.2025.09.013)

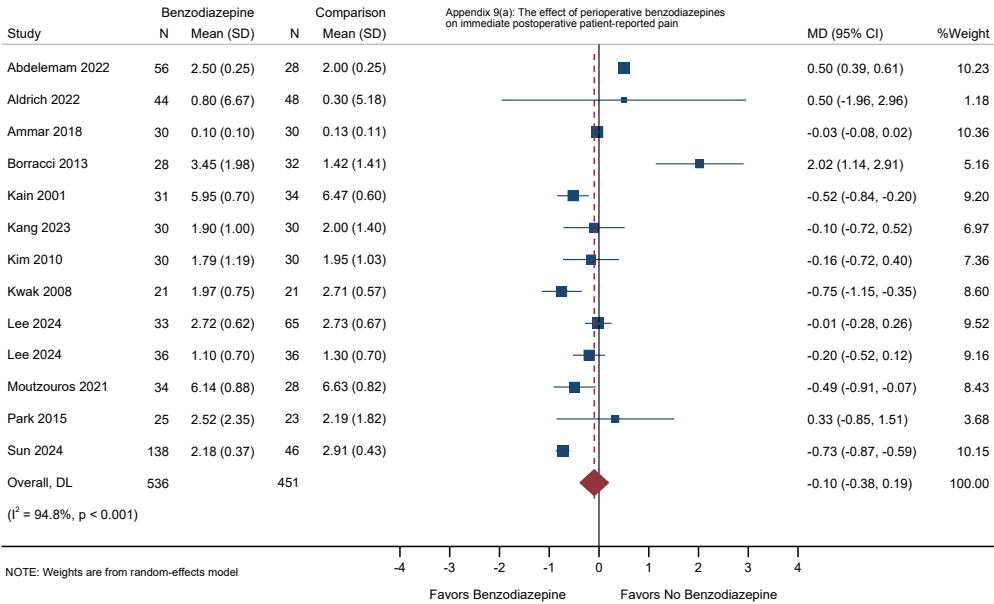

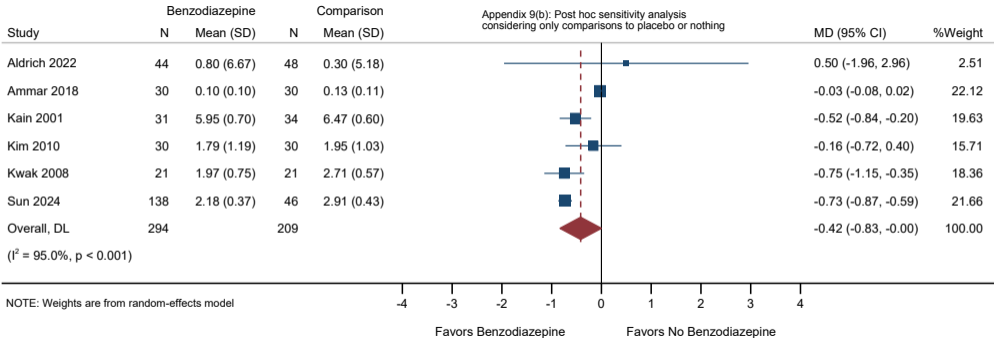

Supplement: Multimedia component 9 [file mmc9.pdf]

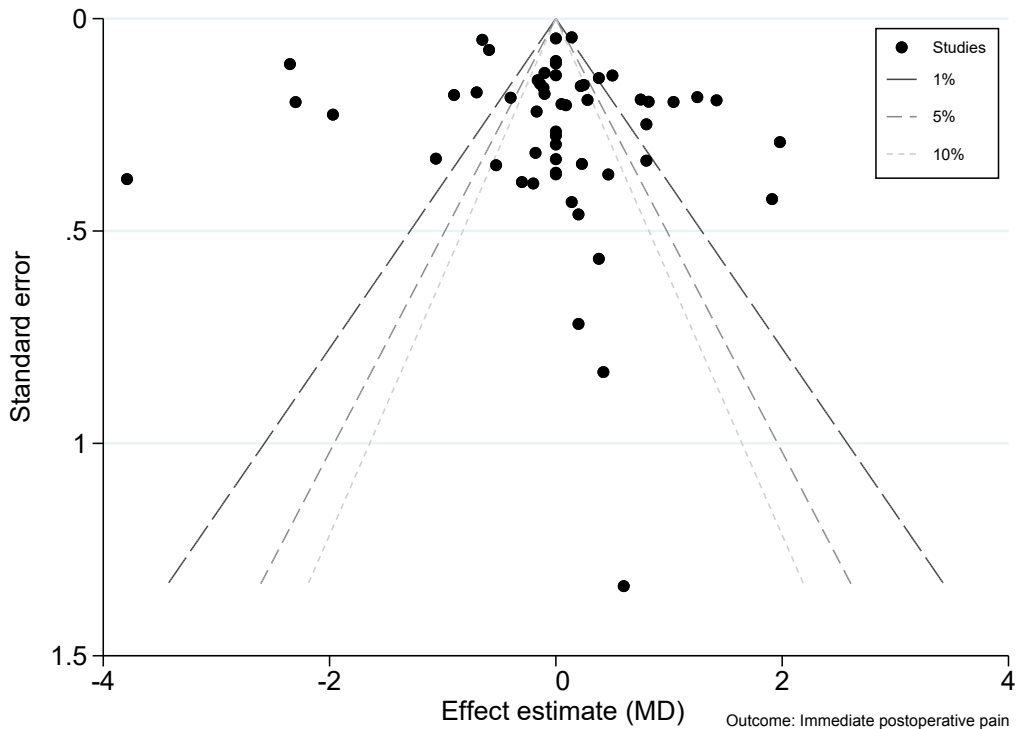

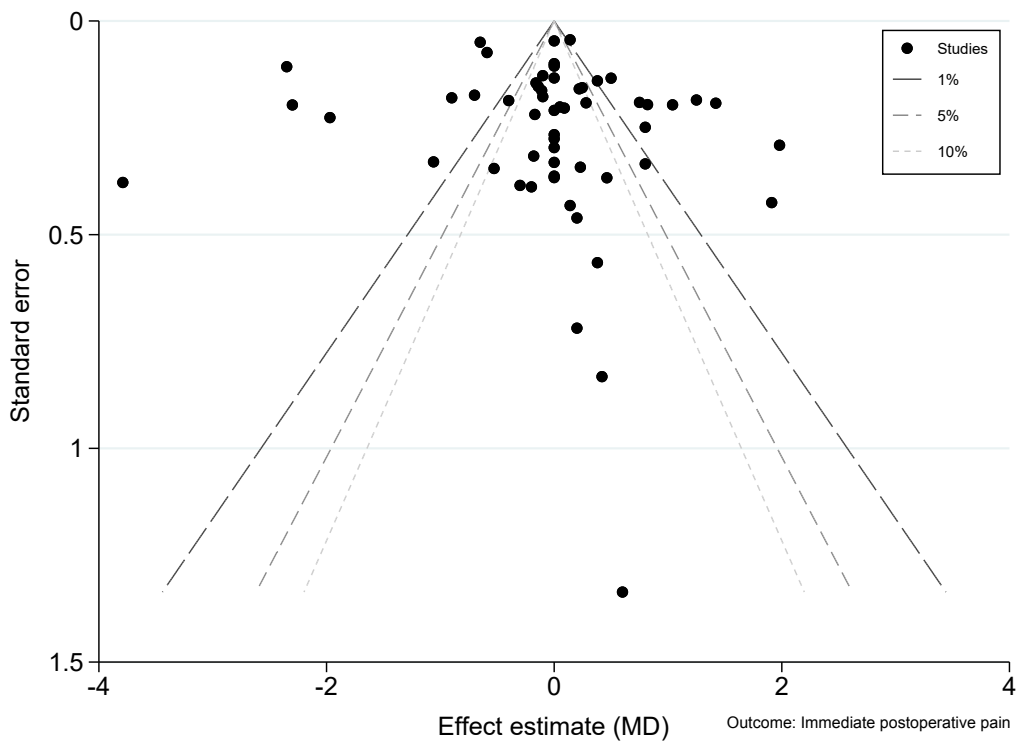

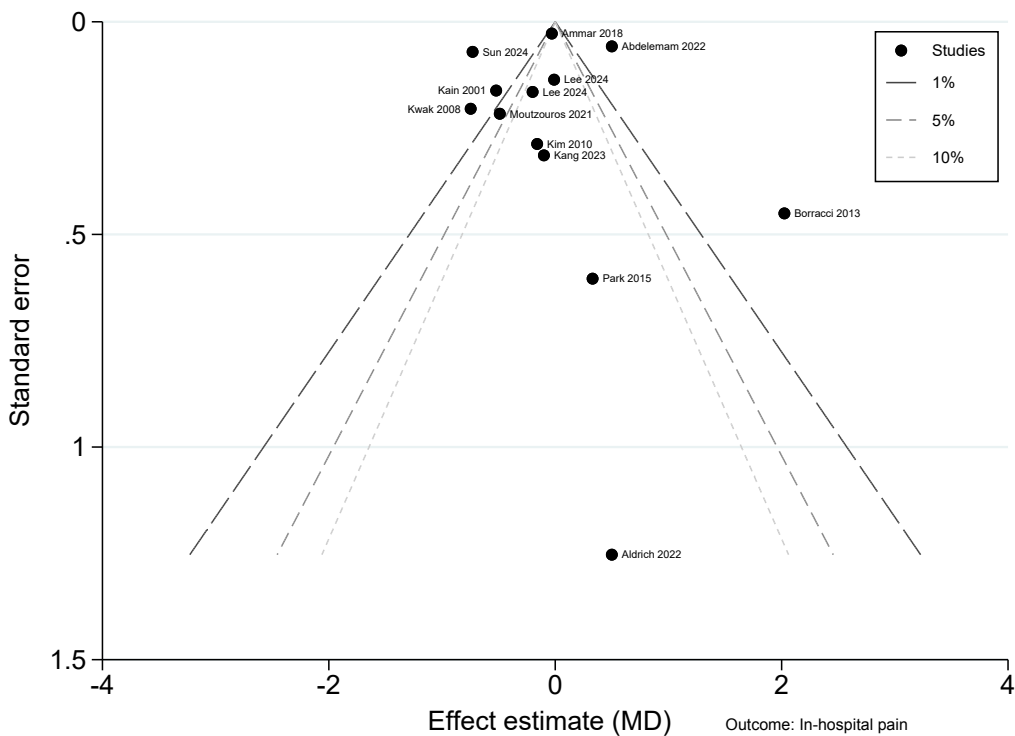

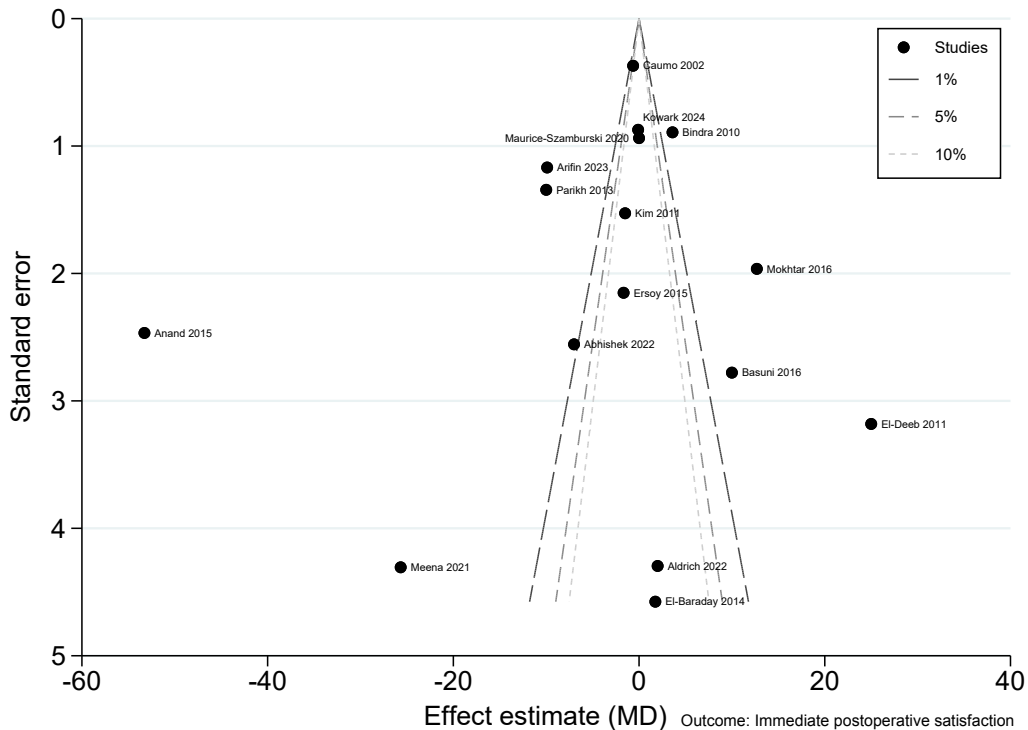

Supplement: Multimedia component 12 [file mmc12.pdf]
