# Supplementary material for: Effects of perioperative benzodiazepine administration on postoperative patient-reported outcomes: a systematic review and meta-analysis of randomised controlled trials
Source: Br J Anaesth. 2025 Sep 30;135(6):1741–52. doi: 10.1016/j.bja.2025.09.013 (PMC12799406; doi:10.1016/j.bja.2025.09.013)
Supplement: Multimedia component 10 [file mmc10.docx]

**Appendix 9: Subgroup analyses**

| **Outcome/subgroup** | | **# of trials** | **WMD (95% CI)** | **N analyzed** | | **I^2^** | **P value for test of interaction*** |
| --- | --- | --- | --- | --- | --- | --- | --- |
|  |  |  |  | **BNZ** | **Comparator** |  |  |
| **Pain (immediate postoperative)**  0 to 10 cm VAS; lower is better | Female only | 21 | 0.00 (-0.21, 0.21) | 1039 | 1006 | 90.8% | 0.923 |
|  | Mixed | 35 | -0.04 (-0.34, 0.26) | 2075 | 2443 | 96.4% |  |
|  | Age ≥ 65 years | 3 | 0.78 (-0.91, 2.47) | 138 | 145 | 90.7% | 0.196 |
|  | Age < 65 years | 53 | -0.08 (-0.28, 0.12) | 2976 | 3304 | 96.0% |  |
|  | Active comparator | 35 | 0.13 (-0.12, 0.38) | 1670 | 1816 | 96.5% | 0.080 |
|  | Placebo | 24 | -0.26 (-0.65, 0.14) | 1540 | 1633 | 96.5% |  |
|  | Remimazolam | 10 | -0.02 (-0.10, 0.05) | 590 | 537 | 0.0% | 0.944 |
|  | Non-remimazolam | 46 | -0.03 (-0.25, 0.19) | 2716 | 3064 | 96.9% |  |
|  | High risk of bias | 13 | -0.30 (-0.72, 0.12) | 761 | 831 | 98.2% | 0.259 |
|  | Low risk of bias | 43 | 0.04 (-0.18, 0.26) | 2353 | 2618 | 92.6% |  |
|  | ***Total*** | 56 | -0.05 (-0.24, 0.15) | 3114 | 3449 | 95.8% | **-** |
| **Pain (in-hospital)**  0 to 10 cm VAS; lower is better | Female only | 4 | -0.51 (-0.79, -0.24) | 126 | 133 | 18.4% | 0.291 |
|  | Mixed | 9 | 0.03 (-0.31, 0.36) | 410 | 318 | 96.2% |  |
|  | Age ≥ 65 years | 2 | 1.71 (0.50, 2.92) | 72 | 80 | 23.6% | **0.004** |
|  | Age < 65 years | 11 | -0.22 (-0.50, 0.06) | 464 | 371 | 95.2% |  |
|  | Active comparator | 7 | 0.20 (-0.22, 0.61) | 242 | 242 | 89.2% | 0.096 |
|  | Placebo | 6 | -0.42 (-0.83, -0.00) | 294 | 209 | 95.0% |  |
|  | Remimazolam | 2 | -2.13 (-7.11, 2.86) | 174 | 173 | 68.6% | 0.924 |
|  | Non-remimazolam | 11 | 3.98 (-2.65, 10.62) | 114 | 113 | 43.4% |  |
|  | High risk of bias | 4 | -0.73 (-0.87, -0.59) | 236 | 173 | 86.5% | 0.147 |
|  | Low risk of bias | 9 | 0.10 (-0.23, 0.42) | 300 | 278 | 92.5% |  |
|  | ***Total*** | 13 | -0.10 (-0.38, 0.19) | 536 | 451 | 94.8% | **-** |
| **Quality of recovery (immediate postoperative)**  0 to 150-point; higher is better | Female only | 3 | -2.27 (-7.95, 3.40) | 141 | 142 | 67.3% | 0.680 |
|  | Mixed | 6 | -0.10 (-7.75, 7.55) | 253 | 254 | 82.5% |  |
|  | Age ≥ 65 years | 1 | -3.00 (-12.73, 6.73) | 17 | 17 | 0.0% | - |
|  | Age < 65 years | 8 | ​​-0.83 (-6.05, 4.38) | 377 | 379 | 79.8% |  |
|  | Active comparator | 8 | -1.31 (-6.92, 4.30) | 354 | 355 | 79.1% | - |
|  | Placebo | 1 | 0.94 (-3.94, 5.81) | 40 | 41 | 0.0% |  |
|  | Remimazolam | 6 | -2.35 (-9.01, 4.32) | 253 | 254 | 79.9% | 0.462 |
|  | Non-remimazolam | 3 | 2.05 (-2.12, 6.22) | 141 | 142 | 25.7% |  |
|  | High risk of bias | - | - | - | - | - | - |
|  | Low risk of bias | - | - | - | - | - |  |
|  | ***Total*** | 9 | -1.05 (-5.83, 3.73) | 394 | 396 | 77.0% | **-** |
| **Quality of recovery (in-hospital)**  0 to 150-point; higher is better | Female only | - | - | - | - | - | - |
|  | Mixed | - | - | - | - | - |  |
|  | Age ≥ 65 years | 2 | 1.48 (-4.55, 7.50) | 53 | 54 | 0.0% | 0.366 |
|  | Age < 65 years | 3 | -0.33 (-7.58, 6.92) | 235 | 232 | 89.6% |  |
|  | Active comparator | 3 | -2.23 (-8.27, 3.81) | 121 | 122 | 60.2% | 0.801 |
|  | Placebo | 2 | 2.70 (-4.34, 9.74) | 167 | 164 | 79.4% |  |
|  | Remimazolam | 3 | -2.13 (-7.11, 2.86) | 174 | 173 | 68.6% | 0.252 |
|  | Non-remimazolam | 2 | 3.98 (-2.65, 10.62) | 114 | 113 | 43.4% |  |
|  | High risk of bias | - | - | - | - | - | - |
|  | Low risk of bias | - | - | - | - | - |  |
|  | ***Total*** | 5 | 0.12 (-5.17, 5.41) | 288 | 286 | 80.5% | **-** |
| **Patient satisfaction (immediate postoperative)**  0 to 100-point; higher is better | Female only | 6 | 8.49 (2.99, 13.98) | 258 | 302 | 96.2% | **0.023** |
|  | Mixed | 10 | -10.60 (-17.74, -3.46) | 951 | 1282 | 96.2% |  |
|  | Age ≥ 65 years | 3 | -0.24 (-1.80, 1.32) | 394 | 396 | 0.0% | 0.714 |
|  | Age < 65 years | 13 | -4.14 (-9.63, 1.34) | 815 | 1188 | 98.4% |  |
|  | Active comparator | 10 | -9.68 (-18.19, -1.18) | 382 | 403 | 98.1% | 0.105 |
|  | Placebo | 7 | 9.62 (1.98, 17.26) | 867 | 1181 | 99.1% |  |
|  | Remimazolam | - | - | - | - | - | - |
|  | Non-remimazolam | - | - | - | - | - |  |
|  | High risk of bias | 2 | -4.92 (-16.21, 6.38) | 35 | 55 | 83.6% | 0.944 |
|  | Low risk of bias | 14 | -3.19 (-7.99, 1.62) | 1174 | 1529 | 98.1% |  |
|  | ***Total*** | 16 | -3.37 (-7.81, 1.08) | 1209 | 1584 | 98.0% | - |
| **Anxiety (immediate postoperative)**  0 to 100-point; lower is better | Female only | 7 | 2.14 (0.29, 3.98) | 255 | 311 | 95.3% | 0.986 |
|  | Mixed | 15 | 2.19 (0.81, 3.57) | 741 | 858 | 74.0% |  |
|  | Age ≥ 65 years | 19 | 2.19 (0.99, 3.39) | 871 | 1046 | 91.6% | 0.925 |
|  | Age < 65 years | 3 | 2.13 (-0.02, 4.28) | 125 | 123 | 0.0% |  |
|  | Active comparator | 16 | 2.95 (1.34, 4.56) | 671 | 693 | 90.6% | 0.520 |
|  | Placebo | 12 | 0.97 (-0.31, 2.25) | 510 | 476 | 81.3% |  |
|  | Remimazolam | - | - | - | - | - | - |
|  | Non-remimazolam | - | - | - | - | - |  |
|  | Low risk of bias | 11 | 1.98 (-0.25, 4.20) | 554 | 594 | 82.3% | 0.697 |
|  | High risk of bias | 11 | 2.47 (1.00, 3.94) | 442 | 575 | 93.7% |  |
|  | ***Total*** | 22 | 2.18 (1.05, 3.30) | 996 | 1169 | 90.2% | - |
