# Supplementary material for: Effects of perioperative benzodiazepine administration on postoperative patient-reported outcomes: a systematic review and meta-analysis of randomised controlled trials
Source: Br J Anaesth. 2025 Sep 30;135(6):1741–52. doi: 10.1016/j.bja.2025.09.013 (PMC12799406; doi:10.1016/j.bja.2025.09.013)
Supplement: Multimedia component 11 [file mmc11.docx]

**Appendix X: Assessment of credibility of subgroup effects using ICEMAN criteria.**

(i) Postoperative Pain (Short-term)

| Subgroup examined | Comparison within vs between RCTs | If within-trial comparisons available, is effect modification similar? | Is the number of studies large? | Direction of effect hypothesized a priori? | Test for interaction magnitude | Small number of subgroups examined | Random effects model | Overall credibility |
| --- | --- | --- | --- | --- | --- | --- | --- | --- |
| Females only vs mixed sex | Completely between | n/a | Very small | Definitely yes | Chance very likely | Probably no | Definitely yes | Very low credibility |
| Elderly (≥65 years) vs younger | Completely between | n/a | Very small | Definitely yes | Chance very likely | Probably no | Definitely yes | Very low credibility |
| Active comparator vs placebo or nothing | Completely between | n/a | Rather large | Definitely yes | Chance very likely | Probably no | Definitely yes | Low credibility |
| Remimazolam vs all other benzodiazepines | Completely between | n/a | Large | Definitely yes | Chance very likely | Probably no | Definitely yes | Low credibility |
| High compared to low ROB | Completely between | n/a | Large | Definitely yes | Chance very likely | Probably no | Definitely yes | Low credibility |

(ii) Postoperative Pain (Longer-Term)

| Subgroup examined | Comparison within vs between RCTs | If within-trial comparisons available, is effect modification similar? | Is the number of studies large? | Direction of effect hypothesized a priori? | Test for interaction magnitude | Small number of subgroups examined | Random effects model | Overall credibility |
| --- | --- | --- | --- | --- | --- | --- | --- | --- |
| Females only vs mixed sex | Completely between | n/a | Very small | Definitely yes | Chance very likely | Probably no | Definitely yes | Very low credibility |
| Elderly (≥65 years) vs younger | Completely between | n/a | Very small | Definitely yes | Chance an unlikely explanation | Probably no | Definitely yes | Low credibility |
| Active comparator vs placebo or nothing | Completely between | n/a | Large | Definitely yes | Chance very likely | Probably no | Definitely yes | Low credibility |
| Remimazolam vs all other benzodiazepines | Completely between | n/a | Rather large | Definitely yes | Chance very likely | Probably no | Definitely yes | Low credibility |
| High compared to low ROB | Completely between | n/a | Rather small | Definitely yes | Chance very likely | Probably no | Definitely yes | Low credibility |

(iii) Quality of Recovery (Short-term)

| Subgroup examined | Comparison within vs between RCTs | If within-trial comparisons available, is effect modification similar? | Is the number of studies large? | Direction of effect hypothesized a priori? | Test for interaction magnitude | Small number of subgroups examined | Random effects model | Overall credibility |
| --- | --- | --- | --- | --- | --- | --- | --- | --- |
| Females only vs mixed sex | Completely between | n/a | Rather small | Definitely yes | Chance very likely | Probably no | Definitely yes | Low credibility |
| Elderly (≥65 years) vs younger^*^ | Completely between | n/a | Very small | Definitely yes | - | Probably no | Definitely yes | Very low credibility |
| Active comparator vs placebo or nothing^*^ | Completely between | n/a | Very small | Definitely yes | - | Probably no | Definitely yes | Very low credibility |
| Remimazolam vs all other benzodiazepines | Completely between | n/a | Rather small | Definitely yes | Chance very likely | Probably no | Definitely yes | Low credibility |
| High compared to low ROB^**^ | - | - | - | - | - | - | - | - |

^*^Unable to assess test of interaction due to <2 studies per subgroup

^**^Unable to conduct subgroup analysis due to no studies in one of the two subgroups

(iii) Quality of Recovery (Longer-term)

| Subgroup examined | Comparison within vs between RCTs | If within-trial comparisons available, is effect modification similar? | Is the number of studies large? | Direction of effect hypothesized a priori? | Test for interaction magnitude | Small number of subgroups examined | Random effects model | Overall credibility |
| --- | --- | --- | --- | --- | --- | --- | --- | --- |
| Females only vs mixed sex^**^ | - | - | - | - | - | - | - | - |
| Elderly (≥65 years) vs younger | Completely between | n/a | Very small | Definitely yes | Chance very likely | Probably no | Definitely yes | Very low credibility |
| Active comparator vs placebo or nothing | Completely between | n/a | Very small | Definitely yes | Chance very likely | Probably no | Definitely yes | Very low credibility |
| Remimazolam vs all other benzodiazepines | Completely between | n/a | Very small | Definitely yes | Chance very likely | Probably no | Definitely yes | Very low credibility |
| High compared to low ROB^**^ | - | - | - | - | - | - | - | - |

^*^Unable to assess test of interaction due to <2 studies per subgroup

^**^Unable to conduct subgroup analysis due to no studies in one of the two subgroups

iv) Patient Satisfaction

| Subgroup examined | Comparison within vs between RCTs | If within-trial comparisons available, is effect modification similar? | Is the number of studies large? | Direction of effect hypothesized a priori? | Test for interaction magnitude | Small number of subgroups examined | Random effects model | Overall credibility |
| --- | --- | --- | --- | --- | --- | --- | --- | --- |
| Females only vs mixed sex | Completely between | n/a | Rather small | Definitely yes | Chance a likely explanation | Probably no | Definitely yes | Low credibility |
| Elderly (≥65 years) vs younger | Completely between | n/a | Very small | Definitely yes | Chance very likely | Probably no | Definitely yes | Very low credibility |
| Active comparator vs placebo or nothing | Completely between | n/a | Rather small | Definitely yes | Chance very likely | Probably no | Definitely yes | Low credibility |
| Remimazolam vs all other benzodiazepines^**^ | - | - | - | - | - | - | - | - |
| High compared to low ROB | Completely between | n/a | Very small | Definitely yes | Chance very likely | Probably no | Definitely yes | Very low credibility |

^**^Unable to conduct subgroup analysis due to no studies in one of the two subgroups

v) Postoperative Anxiety:

| Subgroup examined | Comparison within vs between RCTs | If within-trial comparisons available, is effect modification similar? | Is the number of studies large? | Direction of effect hypothesized a priori? | Test for interaction magnitude | Small number of subgroups examined | Random effects model | Overall credibility |
| --- | --- | --- | --- | --- | --- | --- | --- | --- |
| Females only vs mixed sex | Completely between | n/a | Rather small | Definitely yes | Chance very likely | Probably no | Definitely yes | Low credibility |
| Elderly (≥65 years) vs younger | Completely between | n/a | Very small | Definitely yes | Chance very likely | Probably no | Definitely yes | Very low credibility |
| Active comparator vs placebo or nothing | Completely between | n/a | Large | Definitely yes | Chance very likely | Probably no | Definitely yes | Low credibility |
| Remimazolam vs all other benzodiazepines^**^ | - | - | - | - | - | - | - | - |
| High compared to low ROB | Completely between | n/a | Large | Definitely yes | Chance very likely | Probably no | Definitely yes | Low credibility |

^**^Unable to conduct subgroup analysis due to no studies in one of the two subgroups
